# Taxonomic revision of Australian *Copelatus* Erichson, 1832 (Coleoptera, Dytiscidae, Copelatinae)

**DOI:** 10.3897/zookeys.889.39090

**Published:** 2019-11-14

**Authors:** Lars Hendrich, Helena Shaverdo, Jiří Hájek, Michael Balke

**Affiliations:** 1 SNSB-Zoologische Staatssammlung, Münchhausenstrasse 21, D-81247 München, Germany SNSB-Zoologische Staatssammlung München Germany; 2 Naturhistorisches Museum, Burgring 7, 1010 Vienna, Austria Naturhistorisches Museum Vienna Austria; 3 Department of Entomology, National Museum, Cirkusová 1740, CZ-193 00 Praha 9, Horní Počernice, Czech Republic Department of Entomology, National Museum Praha Czech Republic

**Keywords:** Australia, DNA taxonomy, new species, re-descriptions, synonym

## Abstract

The genus *Copelatus* in Australia is revised and nine species are recognised. One new species, *Copelatus martinbaehri***sp. nov.**, is described from Papua New Guinea (Central Province) and Cape York Peninsula (Iron Range NP and Mt Tozer). *Copelatus divisus* Watts, 1978, **syn. nov.**, is considered a junior synonym of *C. portior* Guignot, 1956, described from New Guinea. Species delimitation is based on the morphological characters and Cox1 data. All species are (re)described, and their important species characters (median lobes, parameres, habitus and colour patterns) are illustrated. A key to all nine species is provided. The known distribution and habitat preferences of each species are outlined briefly. In Australia, all nine species are distributed in the northern half of the continent. Four species are also reported from New Guinea: in addition to *C. martinbaehri***sp. nov.**, we record *C. clarki* Sharp, 1882 for the first time from southern New Guinea, and consider literature records of *C. irregularis* W.J. Macleay, 1871 and *C. marginatus* Sharp, 1882 from New Guinea as doubtful. *Copelatus portior* is widely distributed in Australasia, while *C. tenebrosus* is widely distributed in the Indomalayan and Australasian realms. All Australian *Copelatus* are confirmed to be lentic, found in a large variety of stagnant water, mainly in lowland areas up to 250 m.

## Introduction

The genus *Copelatus* Erichson, 1832 has a worldwide distribution, with highest diversity in the tropics (Nilsson and Hájek 2019). With more than 400 described species, it represents the most speciose genus of the family Dytiscidae (Nilsson and Hájek 2019, Ranarilalatiana et al. 2019). *Copelatus* species inhabit a large variety of both running and stagnant water, mainly in forested areas of the tropics, and have been recorded in South America also from water tanks inside bromeliad plants (Balke et al. 2008) and on Madagascar from wet leaf litter on tropical forest floors (Ranarilalatiana and Bergsten 2019). Recently one species from Brazil was described from a cave (Caetano et al. 2013).

Most species of *Copelatus* are characterised by longitudinal elytral striae, which number has been used to group the species into species groups (Sharp 1882; Guignot 1961; Guéorguiev 1968), although this character does not always delineate monophyletic groups (Balke et al. 2004; Hájek et al. 2010, 2018). In fact, elytral striation can vary even within species and, thus, the use of this character contributes to confusion in the current classification of *Copelatus* (Bilardo and Rocchi 2004, 2015; Megna and Epler 2012; Hájek et al. 2018; Manuel et al. 2018).

The Australian Copelatini species have last been revised by Watts (1978), who has recognised 24 *Copelatus* species. In his key, he divided them into species with “elytron with 4 or more sharply incised longitudinal striae” and species with “elytron without longitudinal striae or with numerous very short striae”. Later, the representatives without longitudinal striae were transferred into the genus *Exocelina* Broun, 1886 (Nilsson and Fery 2006; Nilsson 2007). The remaining eight species comprise the Australian *Copelatus* and occur from the tropical north along the east coast south to southern Queensland and along the west coast south to the Pilbara. Most of them are endemic to Australia (Hendrich et al. 2019). Three species, *C. clarki* Sharp, 1882 (by the present record), *C. irregularis* W.J. Macleay, 1871 and *C. marginatus* Sharp, 1882 (old literature records, doubtful) are considered to have wider areas including New Guinea. *Copelatus portior* Guignot, 1956 is widely distributed in Australasia, while *C. tenebrosus* Régimbart, 1880 is known from many parts of Indomalayan and Australasian realms.

All species of Australian *Copelatus* are found in small rock, residual and side pools of streams and intermittent creeks, in forest pools, and at flooded lake and river margins, often rich in submerged and emerged vegetation, and with decaying plant material. They occur mainly in lowland areas up to 250 m.

During our recent work on Australasian Copelatini, we have recognised one *Copelatus* species hitherto unknown to science; in addition, one new synonym has been discovered. The (re)description of all nine taxa and clarification of their identity, distribution, and habitat preferences are the aims of the present paper.

## Materials and methods

This study is based on the examination of 1,682 specimens. Type material of most species, except for *C. daemeli* and *C. irregularis* (see under those species) was examined. Furthermore, the first author has studied all available specimens stored in relevant Australian museums and private collections.

The material used for this study is deposited in the following institutional and private collections:

**AMS**Australian Museum, Sydney, New South Wales, Australia;

**ANIC**Australian National Insect Collection, Canberra, Australia;

**NHMUK**The Natural History Museum [formerly British Museum (Natural History)], London, England, BMNH;

**CGW** Collection G. Wewalka, Vienna, Austria;

**CLH** Collection L. Hendrich, Berlin, Germany; property of the NMW;

**HNHM**Magyar Természettudományi Múzeum [Hungarian Natural History Museum], Budapest, Hungary;

**MAGNT**Museum and Art Gallery of the Northern Territory, Darwin, Northern Territory, Australia;

**MZB**Museum Zoologicum Bogoriense, Cibinong, Indonesia;

**NMPC** Národní Muzeum, Praha, Czech Republic;

**NMW**Naturhistorisches Museum Wien, Vienna, Austria;

**QDPIB**Queensland Department of Primary Industries, Brisbane, Queensland, Australia;

**QM**Queensland Museum, Brisbane, Queensland, Australia;

**SAMA** South Australian Museum, Adelaide, South Australia, Australia;

**ZSM**Zoologische Staatssammlung, München, Munich, Germany.

Beetles were studied with a Leica MZ 12.5 dissecting scope at 10–100×. Habitus photos were made by František Slamka (Bratislava, Slovakia) using a digital photo imaging system and incident light, composed of a Leica DM 2500 M microscope and a Tucsen 5.0 MP camera. The microscope was fitted with Leica HCX PL “Fluotar” 5× and 10× metallurgical grade lenses (Buffington and Gates 2008). Image stacks were aligned and assembled with the computer software Helicon Focus 4.77^TM^. Male genitalia were studied and figured in wet condition. Aedeagus images were captured by Harald Schillhammer (Vienna, Austria) with a Nikon D4 (in combination with a Novoflex bellows and a Mitutoyo 10/0.25 Apo ELWD) tethered to a PC and controlled with Nikon Camera Control Pro. Resulting image stacks were treated with Zerene Stacker and then post-processed in Adobe Photoshop CS 5.

The descriptive style follows Watts (1978) and Hájek et al. (2018). Label data of all the material are cited in quotation marks. Comments in square brackets are ours. The terminology to denote the orientation of the genitalia follows Miller and Nilsson (2003).

Abbreviations used in the text are:

**hw** handwritten,

**MW** maximum width,

**TL** total length,

**TL-H** total length without head.

Coordinates are given in decimal notation unless cited verbatim from labels. Beside various Australian road maps, we also used Google Earth (http://earth.google.com) to locate several localities. The distribution map is based on the records cited here and the results from “Atlas of living Australia” [https://www.ala.org.au/].

We delineate the species using traditionally employed morphological characters such as shape, structure and setation of the male genitalia; size, shape and colour pattern of the body, shape of the male protibia, as well as features of the dorsal surface sculpture.

### DNA sequencing and data analysis

The sequence data partly originate from Hendrich et al. (2010). We preserved a part of our collections in 96% ethanol and later extracted DNA for sequencing. The laboratory methods employed are detailed on our DNA laboratory wiki: https://zsm-entomology.de/wiki/The_Beetle_D_N_A_Lab. PCR conditions with Mango Taq (Bioline) were 1'94 °C – 35× (30 s 94 °C – 30 s 47 °C – 1'72 °C) – 10'72 °C – (hold at 14 °C) with primers Jerry and Pat to amplify and sequence the 3' of the gene encoding for cytochrome c oxidase 1 (Simon et al. 1994). All 46 individual vouchers bear a green cardboard label that indicates the DNA extraction number of M. Balke (e.g. “DNA M. Balke 2291”). This number links the DNA sample, the dried mounted voucher specimen, deposited in ZSM. We used a simple approach to calculate a neighbour joining tree (*p*-distances) in Geneious (11.0.4.) software (Fig. 29) and visual inspection to learn if there was any hidden diversity or haplotype sharing. The sequence data have been deposited at DRYAD (datadryad.org) as a Geneious project and in FASTA file format: https://datadryad.org/stash/dataset/doi: 10.5061/dryad.w0vt4b8m7.

GenBank accession numbers are AY368215–AY368217, EF670049, FR732611, FR732615, FR732620, FR732636, FR732654, FR732730, FR732732, FR732748, FR732802, FR732803, FR732807, FR732808, FR732809, FR732999, FR733000, FR733016, FR733023–FR733026, FR733033–FR733037, FR733047, FR733137, FR733213, FR733285, FR733287, FR733303, FR733425–FR733427, FR733505, FR733558, FR733559, LN717083, LT615908, LT615914.

## Taxonomy

The morphologically delineated species were all retrieved as monophyletic groups in our cox1 DNA sequence tree (Fig. 29). We observe geographic structure for the Western Australian and Queensland individuals of *C. irregularis*, but these are samples from most distant localities only, and the divergence amounts to only c. 1%.

### Genus *Copelatus* Erichson, 1832

Small to medium sized (4.2–8.0 mm in Australia), elongate or oblong-oval and more flattened diving beetles, narrowing towards apex. Scutellum visible. Elytra of all Australian species with longitudinal striae being numbered from innermost to outermost (except submarginal stria). Prosternum and its process in same plane or process slightly deflected upwards behind procoxae. Prosternal process short. Metepisternum extended to mesocoxal cavity. Lateral parts of metaventrite (“metasternal wings”) relatively broad at level of mesocoxa and very narrow laterally. Metacoxal processes with an apical cleft; metacoxal lines present. Metafemur ventrally without setigerous row at outer posterior angle. Tarsi with five distinct tarsomeres; male with protarsomeres 1–3 broadly dilated but not forming a round palette, and with four rows of larger adhesive discs bordered by long and thick setae. Metatarsal claws of same length. Median lobe asymmetrical.

#### Copelatus bakewelli

Taxon classificationAnimaliaColeopteraDytiscidae

J. Balfour-Browne, 1939

C9F8FF17-8AC7-5B21-A3CA-9EEF8A6AFD13

**Figure 1.**
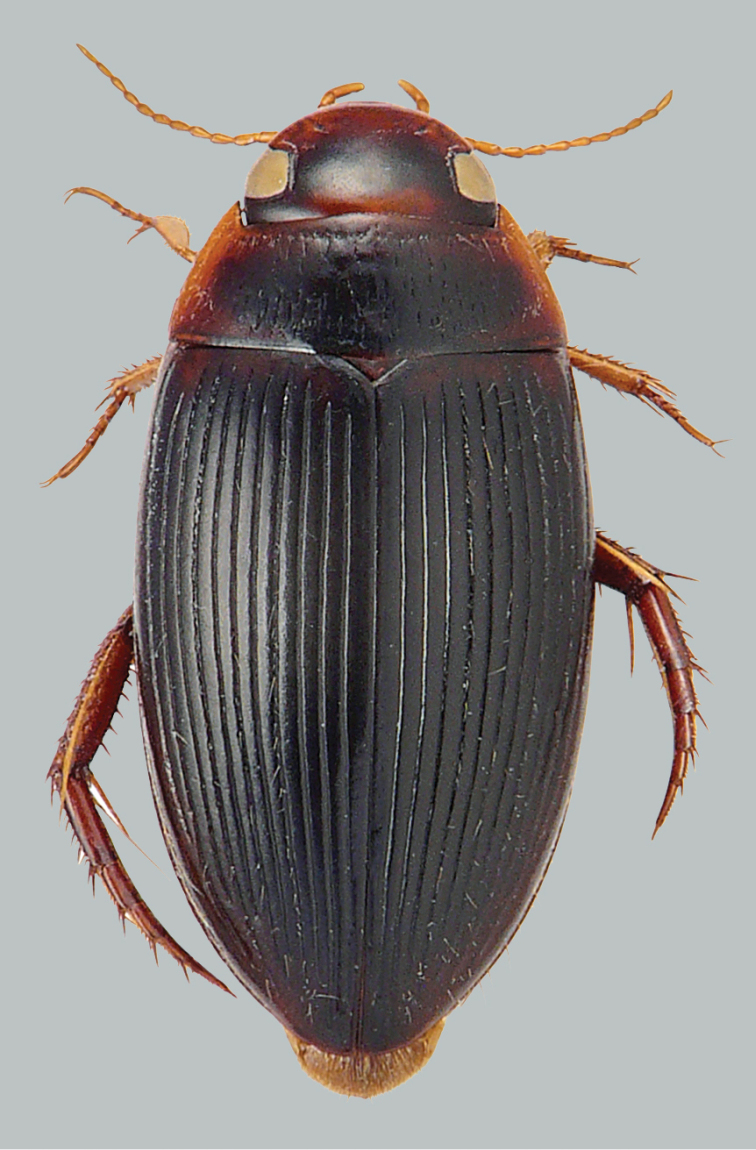
Habitus and colouration of *Copelatus bakewelli*, female. Total length 5.2 mm.

Figures 1 , 11 , 20 , 30

Copelatus bakewelli J. Balfour-Browne 1939: 86 (original description); Watts 1978: 127 (description); Watts 1985: 26 (general distribution); Watts 2002: 42 (checklist); Nilsson and Hájek 2019: 62 (catalogue).

##### Type locality.

“North Australia”.

##### Type material.

***Holotype:*** Female, “Holotype” [round label with red frame], “Na” [hw label], “6756” [printed label], “Copelatus bakewelli B-B. Type” [hw label] (NHMUK).

##### Additional material studied (285 specimens).

***Western Australia:*** 2 exs., “AUSTRALIA WA, Synnot Range 1/9/1969, 16°25'S, 125°28'E, Giuliani D.D. leg.” (WAM); 1 ex., “AUSTRALIA/WA/Shire of Wyndham – East Kimberley, Gibb River Road, Drysdale River Crossing, 380 m, 14.6.1999, Hendrich leg. Loc. 9/109” (CLH); 4 exs., “AUSTRALIA/WA/ Shire of Wyndham-East Kimberley, Gibb River Road, King Edward River Crossing, 280 m, 15.6.1999, Hendrich leg./coll. Loc. 10/110” (CLH); 77 exs., “AUSTRALIA/WA/Shire of Wyndham-East Kimberley, Mitchell Plateau, Little Mertens Falls, 300 m, 15.6.1999, Hendrich leg./coll. Loc. 11/111b” (CLH, ZSM); 8 exs., “AUSTRALIA/WA/ East Kimberley, Mitchell Plateau, Little Mertens Falls, 300 m, 15.6.1999, Hendrich leg./coll. Loc. 11a/111a” (NMW); 2 exs., “AUSTRALIA/WA/Shire of Wyndham – East Kimberley, Mitchell Plateau, Port Warrender Road/Kalumburu Road, Lowya Creek, 290 m, 18.6.1999, Hendrich leg. Loc. 13/113” (CLH); 1 ex., “AUSTRALIA/WA/Shire of Wyndham - East Kimberley, Mitchell Plateau, Kalumburu Road, 25 km NW King Edward Homestead, N.N. Creek, 370 m, 18.6.1999, Hendrich leg. Loc. 114” (CLH); 4 exs., “AUSTRALIA/WA/Shire of Wyndham - East Kimberley, Gibb River Road, 10 km E Hann River, Snake Creek, 470 m, 19.6.1999, Hendrich leg. Loc. 16/116” (CLH). ***Northern Territory***: 1 ex., “Australia: NT, Cullen River n. Cullen, at Stuart Hwy, 102 m, 23.VIII.2006, 14.02.052S 131.56.561E, L. & E. Hendrich leg. (NT 13)”, “DNA Balke 2213” [green printed label] (ZSM); 1 ex., “Australia: NT, Kakadu Hwy, Harriet Creek at Hwy Cross., 153 m, 24.VIII.2006, 13.44.512S 131.54.012E, L. & E. Hendrich leg. (NT 14)”, “DNA Balke 1614” [green printed label] (ZSM); 2 exs., “Australia: NT, Litchfield NP, Creek near Wanggi Falls, 191 m, 20.VIII.2006, 13.11.221S 130.43.327E, L. & E. Hendrich leg. (NT 4)”, “DNA Balke 2190”, “DNA Balke 2191” [green printed label] (ZSM); 2 exs., “AUSTRALIA NT Kakadu NP Alligator R. Gungaree Rainforest Dec. 22/93 S&J Peck” (ZSM); 1 ex., “NT, Horse Creek fossil site, Camfield Station [-17.1166 131.5166] 2/8/1992”, “1004435” (MAGNT); 1 ex., “NT, Top Humbert Yard, Humbert River [-16.4333, 130.4666] 7/1986”, “1004443” (MAGNT); 2 exs., “Bathurst Is. + Melville Is. N.T. XII. 1975 D. Curry”, “QDPC 0-172712”, “QDPC 0-172713” (QDPC); 2 exs., “Australia, NT, Litchfield NP, Florence Falls, 12°51'15"S, 132°45'16"E, 63 m, at light, 12.V.2006, leg. Berger & Dostal (5/06)” (CGW); 8 exs., “Australia, NT, 80 km W Roper Bar, 14°54'19"S, 133°57'28"E, 78 m, at light, 14.V.2006, leg. Berger & Dostal (7/06)” (CGW); 1 ex., “Australia, N.T./Katherine Gorge, Butterfly Gorge Walk, 150 m, 4.7.1999, Hendrich leg. Loc. 33/133” (CLH); 3 exs., “Australia, N.T. Kakadu N.P., Mary River District, 3 km ESE Gunlom Camping Area. South Alligator River, 50 m, 2.11.1996, 13°27.276'S, 132°26.268'E L. Hendrich leg./Lok. 14” (CLH); 3 exs., “Australia, N.T./Kakadu N.P., Jim Jim District, Gungurul Lookout, 50 m, 1.11.1996, 13°59.359'S, 132°19.904'E L. Hendrich leg./Lok. 11” (CLH); 19 exs., “Australia, N.T./Kakadu N.P., Mary River District, Gunlom Camping Area, 50 m, 3.11.1996, 13°26.082'S, 132°24.929'E L. Hendrich leg./Lok. 15” (CLH); 2 exs., “Australia, N.T./Kakadu N.P., Jim Jim District, Barramundie Gorge, Maguk, 50 m, 31.10.1996, 13°18.823'S, 132°26.198'E. L. Hendrich leg./ Lok. 9” (CLH); 12 exs., “Australia, NT/Kakadu N.P., Mary River District, Gunlom Waterfall Creek, 150 m, 2.11.1996, 13°26.082'S, 132°24.929'E L. Hendrich leg./Lok. 13” (CLH); 51 exs., “Australia, N.T./Kakadu N.P., Jim Jim District, 1 km S Jim Jim Falls, 70 m, 26.10.1996, 13°16.718'S, 132°49.490'E L. Hendrich leg./Lok. 3” (CLH); 39 exs., “Australia, N.T./Kakadu N.P., Jim Jim District, Jim Jim Falls Camping Area, Jim Jim Creek, 60 m, 26. & 27.10.1996 13°16.218'S, 132°49.276'E L. Hendrich leg./ Lok. 2.” (CLH); 13 exs., “Australia N.T./Kakadu N.P., Nourlangie District, Gubara, 50 m, 25.10.1996, 12°50.101'S, 132°52.501'E L. Hendrich leg./Lok. 1” (CLH); 1 ex., “Australia, NT, Pine Creek, Roper Gulf (S)Waterfall Ck., 60 mi. E of Pine Ck. [-13.81667, 132.7], 7.08.1964, Carne, P.B. leg. (ANIC); 1 ex., “Australia, NT, Daly Basin, Katherine (T) Tindal, -14.51667, 132.3667, 1.12.1967, Vestjens, W.J.M. leg. (ANIC); 10 exs., “Australia: NT, Litchfield NP, TJAYNERA FALLS, 13°15'S, 130°44'E, 63 m, S. Jákl leg., 20–27.XI.2008” (NMPC). ***Queensland***: 2 exs., “QLD Cook, 2 km NNW Jowalbinna [-15.75, 144.25] 17/01/1994 Zborowski P. & Edwards, E.D.”, “25-023624-470” (ANIC); 1 ex., “QLD Cook, 14 km ENE Heathlands [-11.68, 142.7], rainforest, 21/10/1993 Zborowski P. & Rentz D.C.F.”, “25-022999-353” (ANIC); 1 ex., “QLD, Townsville [-19.4833, 146.8167], 25.3.1996, CH Watts”, “25-001942” (SAMA); 1 ex., “QLD, Townsville [-19.2, 146.68], 6–11/2/1998, AJ Watts”, “25-001940” (SAMA); 1 ex., “Bamaga N. Qld. March 1984 J.W. Turner”, “QDPC 0-172711” (QDPC); 3 exs., “Bamaca [=Bamaga] xii.1983”, “Australian QLD J. Sedlacek” (CLH); 1 ex., “Australia, Queensland, Bald Hills Station 4 km N of Isabella Falls, 15°15' 145°00', 29. December 1984, mv lamp, G. & A. Daniels” (QM).

##### Description of male.

***Body shape*.** In dorsal view, oblong oval, broadest at midlength of elytra, moderately convex. Body outline continuous, without discontinuity between pronotum and elytra. Head relatively broad; anterior margin of clypeus not bordered. Pronotum broadest between posterior angles, lateral margins moderately curved. Base of elytra as broad as pronotal base; lateral margins of elytra moderately curved (Fig. 1).

***Colouration:*** Body black, clypeus anteriorly, sides of pronotum, base of elytra, and humeral angle of elytra paler, ferruginous; ventral side reddish, appendages testaceous (Fig. 1).

***Dorsal surface sculpture:*** Whole surface shiny (Fig. 1). Head uniformly microreticulated, reticulation composed of moderately deeply impressed isodiametric meshes. Punctation composed of very small punctures, sparsely spread on surface; rows of deep and coarse punctures present around inner margin of eyes and in small depression anterolaterally of eyes. Pronotum with some moderately strong, short striae, more dense and weak laterally; lateral beading of pronotum very thin and indistinct. Microreticulation and punctation similar to that of head; row of coarse setigerous punctures present along anterior margin, basal margin (except for basomedially), and laterally close to sides. Elytra with microreticulation similar to that of head and pronotum, but less impressed. Punctation consisting of very fine sparse punctures. Each elytron with 11 strongly impressed discal and one submarginal longitudinal striae, intervals subequal, alternate striae tending to be shorter apically; subbasal stria reaching from middle of elytron almost to end of stria 10.

***Antennae and legs:*** Antenna with antennomeres long and slender. Protibia straight, not modified. Pro- and mesotarsomeres 1–3 distinctly broadened, with adhesive discs on their ventral side; claws simple.

***Ventral part:*** Finely microreticulated, with intermixed, sparsely distributed, very small punctures. Prosternum obtusely keeled medially. Prosternal process shortly lanceolate, apex obtuse; distinctly bordered laterally. Lateral parts of metaventrite tongue-shaped, very slender. Metacoxal lines almost complete and absent only close to metaventrite. Metacoxae with some short and deep striae, and abdominal ventrites 1–3 with numerous longitudinal striae.

***Male genitalia:*** Median lobe consisting of a few sclerites, closely attached together; apex in lateral view thin and elongate (Fig. 11A, B). Shape of paramere narrowly triangular, with thin, elongate subdistal part and weak but evident long setae along dorsal margin (Fig. 11D).

##### Female.

Similar to male in habitus. Pro- and mesotarsomeres not broadened, without adhesive setae. Pronotal striae finer and denser.

##### Measurements.

TL = 5.1–5.25 mm; TL-H = 4.5–4.65 mm; MW = 2.45–2.50 mm.

##### Variability.

All specimens studied are rather uniform in shape and colour, and vary only little in body length.

##### Differential diagnosis.

*Copelatus bakewelli* is close to the form of *C. daemeli* with 11 elytral striae and *C. irregularis* but smaller and without testaceous basal and apical markings on the elytra. Pronotal striae are weak (absent in *C. daemeli*) and the male protibia is not bent as in *C. irregularis*. Furthermore, all three species can easily be separated by the form of their median lobes.

##### Distribution.

Endemic. The species occurs from the Kimberley region in north-western Australia and in the Arnhemland in the Northern Territory to northern Queensland. In that area, the species is mainly distributed in rainforest pockets of the stone country (Fig. 20).

##### Habitat.

A lentic species, which occurs in isolated pools of different size in otherwise dry riverbeds of seasonal streams, creeks and rivers. The bottom is consisted of gravel, sand and a layer of rotten plant debris. Occasionally, it occurs in protected bays, at the edge of large (25–50 m width), slow flowing and shallow (up to 20 cm depth) rivers, shaded by old riverine Melaleuca trees. There, the adults can be found among floating roots and organic debris of the paperbark trees. In northern Australia, at Gunlom waterfall, a large series was collected in a small (ca. 50 cm², 10 cm depth), water filled and shaded pothole (Fig. 30), filled up with rotten leaves. In Kakadu and the Kimberley region, *C. bakewelli* is more or less restricted to the stone country and the monsoon rainforest pockets. The species is also attracted to light.

#### Copelatus clarki

Taxon classificationAnimaliaColeopteraDytiscidae

Sharp, 1882

F69DA1A6-0056-5C0A-886D-C85B5CCDBEC0

**Figure 2.**
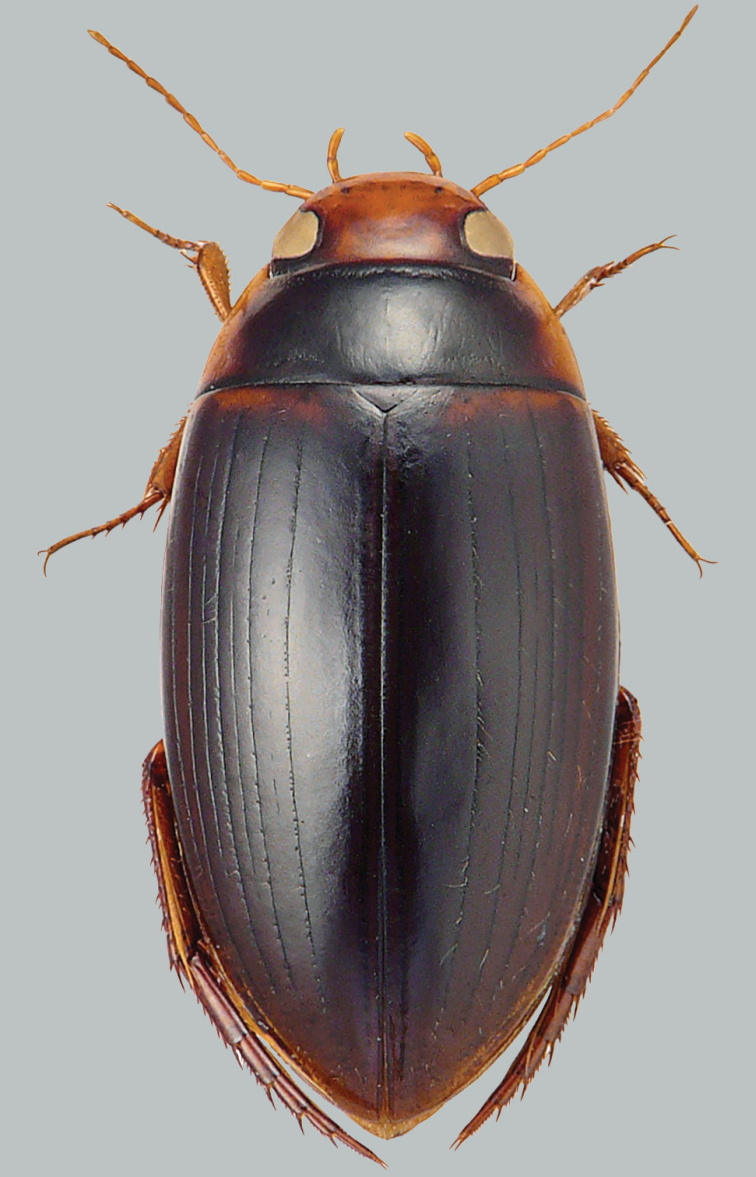
Habitus and colouration of *Copelatus clarki*, female. Total length 8.0 mm.

Figures 2 , 12 , 22 , 31

Copelatus clarki Sharp 1882: 585 (original description); Larson 1993: 52 (habitat information); Larson 1997: 276 (habitat information); Watts 1978: 125 (description); Watts 1985: 26 (general distribution); Watts 2002: 42 (checklist); Nilsson and Hájek 2019: 47 (catalogue).

##### Type locality.

“Australia [Queensland] (Cape York)”.

##### Type material.

***Lectotype*** (designated by Watts 1978: 125): Male, “Lectotype” [round label with violet frame], “Type” [round printed label with red frame], “Cape York. 676” [hw label], “Sharp Coll. 1905-313.” [printed label], “Type to 76 Copelatus clarki n. sp. Cape Yorke” [hw label], “Copelatus clarki Sharp Det. C. Watts det. 1971” [hw label] (NHMUK). ***Paralectotype***: Female, “Paralectotype” [round label with blue frame], “Cotype” [roundish label with yellow frame], “Cape York. 676” [hw label], “Sharp Coll. 1905-313.” [printed label], “Copelatus clarki Cap York” [hw label], “Copelatus clarki Sharp Det. C. Watts det. 1971” [hw label] (NHMUK).

##### Additional material studied from Australia (304 specimens).

***Western Australia:*** 4 exs., “AUSTRALIA/WA/ Shire of Wyndham-East Kimberley, Gibb River Road, King Edward River Crossing, 280 m, 15.6.1999, Hendrich leg./Coll./Loc. 10/110” (CLH); 1 ex., “Australia, WA/Shire of Wyndham-East Kimberley, Kalumburu Road, Meelarie Creek, 5 km N Drysdale Crossing, 350 m, 18.–19.6.1999, Hendrich leg. Loc. 15/115” (CLH). ***Northern Territory***: 1 ex., “Northern Territory, Magela Ck, nr. rum pipe [Mudginberri Homestead], Pine Creek [-12.7, 132.95], 7.1.1987, Dostine, P.” (ANIC); 2 exs., “Australia07, NT 112, Mary River NP, Mary River Billabong 5 km nnw. Mary River Creek 12.53.49S, 131.38.33E, 28 m, 4.–5.12.2007, M. Baehr” (ZSM); 2 exs., “Australis07, NT 109, Kakadu NP Jim Jim Billabong, 12 km SSE Cooinda 12.56.26S, 132.33.10E, 16 m, 2.–3.12.2007, M. Baehr” (ZSM); 1 ex., “Australia07, NT3 Litchfield NP Tabletop Swamp, 212 m, 13.10.65S 130.44.82E 30.–31.10.2007, M. Baehr” (ZSM); 1 ex., “Australia07, NT13, Kakadu NP, South Alligator R. Cr., Old Jim Jim Rd., 43 m, 13.02.95S 132.19.13E, 3.–4.11.2007 M. Baehr” (ZSM); 1 ex., “Australia: NT, Manton Dam Recr. Area, 46 km S Darwin, 35 m, 19.VIII.2006, 12.50.270S 131.08.050E, L. & E. Hendrich leg. (NT 1)”, “DNA Balke 1658” [green printed label] (ZSM); 2 exs., “Australia: NT, Anniversary Creek, 12 km S Adelaide River Scenic Route, 43 m, 22.VIII.2006, 13.19.252S 131.08.271E, L. & E. Hendrich leg. (NT 7)”, “DNA Balke 1639”, “DNA Balke 2176” [green printed label] (ZSM); 1 ex., “Australia NT / Litchfield N.P., Florence Falls Rainforest Walk, 120 m, 4.11.1996 13°06.705'S, 130°47.220'E L. Hendrich leg. /Lok.17” (CLH); 2 exs., “Australia NT/ Kakadu N.P., Mary River District, Gunlom Waterfall Creek, 150 m, 2.11.1996 13°26.082'S, 132°24.929'E L. Hendrich leg./Lok.13” (CLH); 1 male, “Australia NT/ Litchfield N.P., Florence Falls Camping Area, 120 m, 4.11.1996 13°06.705'S, 130°47.220'E L. Hendrich leg./Lok. 16” (CLH); 1 ex., “Australia NT/ Kakadu N.P., Jim Jim District, Barramundie Gorge, Maguk, 50 m, 31.10.1996 13°18.823'S, 132°26.198'E L. Hendrich/Lok. 9” (CLH); 7 exs., “Australia NT/ Kakadu N.P., Jim Jim District, Jim Jim Falls Camping Area, Jim Jim Creek, 60 m, 26. & 27.10.1996 13°16.218'S, 132°49.276'E L. Hendrich leg./Lok. 2” (CLH); 9 exs., “Australia NT/ Kakadu N.P., Jim Jim District, Gungurul Lookout, 50 m, 1.11.1996 13°59.359'S, 132°19.904'E, L. Hendrich leg./Lok. 11” (CLH); 1 ex., Australia, NT, Pine Creek, Brocks Ck. [-13.46667, 131.4167], 25.4.1932, Campbell, T.G. (ANIC); 1 ex., “Australia: NT, Georgetown Billabong, 750 m E Jabiru East, 30 m, 29.VIII.2006, 12.40.716S 132.55.861E, L. & E. Hendrich leg. (NT 20)” (ZSM); 1 ex., “Australia: NT, Kakadu NP, Sandy Billabong 6 km S Muriella Park Camp. Area, 20 m, 31.VIII.2006, 12.54.077S 132.46.411E, L. & E. Hendrich leg. (NT 25)” (ZSM); 5 exs., “Australia, NT, Darwin, 12°51'24"S, 131°46'48"E, 52 m, at light, leg. Berger & Dostal (2/06)” (CGW); 1 ex., “Australia, NT, Litchfield NP, Florence Falls, 12°51'15"S, 132°45'16"E, 63 m, at light, 12.V.2006, leg. Berger & Dostal (5/06)” (CGW); 2 exs., “Australia, NT, Kakadu NP, Muirella, 40 m, at light, 10.V.2006, leg. Berger & Dostal (3/06)” (CGW). 46 exs., “Australia: NT, Litchfield NP, TJAYNERA FALLS, 13°15'S, 130°44'E, 63 m, S. Jákl leg., 20–27.XI.2008” (NMPC); 1 ex., “Australia: NT, Nitmiluk NP, Edit Falls, 14°10'S, 132°06'E, 37 m, S. Jákl leg., 3.XII.2008” (NMPC); 5 exs., “Austr., NT, Kakadu NP, Jim Jim Billabong, 12°56'S, 132°33'E, 5 m, 5.–8.12.[20]08, Sv. Bílý leg.” (NMPC); 1 ex., “Austr., NT, Kakadu NP, Gunlom, 11.12.08, 13°26'S, 132°34'E, 64 m, Sv. Bílý leg.” (NMPC); 2 exs., “Australia, NT, Douglas, Hot Springs, 12.12.08, 13°45'S, 131°26'E, 35 m, Sv. Bílý leg.” (NMPC); 1 ex., “Australia NT, 25 km S of Katherine, 168 m, 14°31'S, 132°25'E, 16.–17.1.2009, Sv. Bílý leg.” (NMPC). ***Queensland***: 3 exs., “AUSTRALIA Qld Mareeba S-edge Res. Stn. Sept 30/90 D. Larson” (ZSM); 1 ex., “AUSTRALIA Qld Ellis Beach 30 km N Cairns Jan 11 1991 D. Larson” (ZSM); 1 ex., “AUSTRALIA Qld 15 km N Cairns Jan 11, 1991 D.J. Larson” (ZSM); 3 exs., “QLD, Barkly (S) [-19.5000, 132.5000] Tepper JP”, “25-0017652” (SAMA); 1 ex., “Australia, QL, Marbeeba, 700 m, 22.I.1993, leg. G. Wewalka (19)” (CGW); 1 ex., “Australia, QL, Dalrymple, 30 km N Charters Towers, 300 m, 18.I.1993, leg. G. Wewalka (10, 11)” (CGW); 1 ex., “Australia, QL (16) Ravenshoe, 900 m, 90 km W Innisfail, 20.I.1993, leg. Wewalka” (NMW); 5 exs., “Australia, QL, 15–20 km S Innisfail, 20 m, 24.I.1993, leg. G. Wewalka (24)” (CGW); 5 exs., “Australia, QL, 10 km S Tully, S Innisfail, 30 m, 25.I.1993, leg. G. Wewalka (26)” (CGW); 2 exs., “Australia QLD/30 km NNW Mareeba, near Mitchell lake, 9.11.1996 Hendrich leg./Lok. 20” (CLH); 1 ex., “Australia: N QLD, 30 km NW Mareeba, Lake Mitchell at Developmental R., 381 m, 12.IX.2006, 16.47.499S 145.21.444E, L. & E. Hendrich leg. (QLD 30)”, “DNA Balke 1780” (ZSM); 1 ex., “Australia: N QLD, W Mossman Syndicate Road at Harlows Bridge, 12 m, 14.IX.2006, 16.16.289S 145.23.401E, L. & E. Hendrich leg. (QLD 34)” (CLH); 8 exs., “Australia: N QLD, Cape Tribulation Road S of ferry station, forest swamp, 12 m, 15.IX.2006, 16.17.469S 145.19.122E, L. & E. Hendrich leg. (QLD 35)” (CLH, ZSM); 1 ex., “Australia: C QLD, 18 km S Calen, Mt. Charlton-Calen Road, Boulder Creek, 42 m, 23.IX.2006, 21.00.365S 148.43.231E, L. & E. Hendrich leg. (QLD 45)” (CLH); 3 exs., “Australia: C QLD, 10 km S Mizani, 500 m S Lake Kinchant Camping Area, 46 m, 24.IX.2006, 21.12.599S 148.54.153E, L. & E. Hendrich leg. (QLD 47)” (CLH); 1 ex., “Australia: C QLD, Mackay, 33 km S Sarina, Bolingbroke Road at Railway Cross., 221 m, 24.IX.2006, 21.38.378S 149.08.183E, L. & E. Hendrich leg. (QLD 48)” (ZSM); 3 exs., “Australia: S QLD, 15 km S Agnes Water, entrance Errimbula NP, 15 m, 25.IX.2006, 24.15.193S 151.49.222E, L. & E. Hendrich leg. (QLD 51)” (CLH); 4 exs., “Australia: S QLD, Winfield, Winfield Road, forest pool, 21 m, 26.IX.2006, 24.34.084S 152.00.513E, L. & E. Hendrich leg. (QLD 54)” (CLH); 6 exs., “Australia: S QLD, Bundaberg reg., 2 km W Woodgate, swamp, 33 m, 27.IX.2006, 25.07.325S 152.30.270E, L. & E. Hendrich leg. (QLD 57)” (ZSM; CLH); 4 exs., “Australia: N QLD, 4 km NW Cardwell, Ellerbeck Road, 13 m, 19.IX.2006, 18.14.520S 145.58.458E, L. & E. Hendrich leg. (QLD 38)” (CLH); 4 exs., “Australia: N QLD, 20 km NE Mareeba, Hodzic Road, 361 m, 12.IX.2006, 16.49.556S 145.27.211E, L. & E. Hendrich leg. (QLD 28)”, 2 specimens with “DNA Balke 2504” and “DNA Balke 2506” [green printed labels] (CLH); 3 exs., “Australia: S QLD, 40 km E Bundaberg, Tusky Creek, 9 m, 26.IX.2006, 24.39.139S 152.01.477E, L. & E. Hendrich leg. (QLD 52)” (CLH); 8 exs., “Australia S QLD, N Stradbroke Island, Brown Lake, 60 m, Baumea swamp, 27°29'37.09"S, 153°25'50.08"E, 18.X.2014 Lars Hendrich leg. (QLD1/14)” (ZSM); 1 ex., “Australia, Queensland, Hinchinbrook (S) Ingham [-18.65, 146.1666667], 30.3.1960, Harley, K.L. leg.” (ANIC); 1 ex., “Australia, Queensland, Brigalow Belt North, Townsville [-19.26667023, 146.8166656], 22.12.1967, Ferrar, P.” (ANIC); 1 ex., “Australia, Queensland, Cassowary Coast (R), Forest Marsh Innisfail [-17.53333, 146.0167], 7.9.1965” (ANIC); 1 ex., “Australia, Queensland, Cairns, Green Hill, 5 mi. E of Kamma [between Edmonton and Gordonvale, -17.05, 145.8], 7.12.1968, Brooks, J.G.” (ANIC); 1 ex., “Queensland, 4.5 mi. NE Innisfail, Jubilee Rd. [-17.53333, 146.0167], 4.11.1966, Britton, E.B. leg. (ANIC); 1 ex., “Queensland, Goondi Hill Swamp, Innisfail, 3.11.1966, [-17.51666667, 146.0166667], Britton, E.B.” (ANIC); 1 ex., “Queensland, Cairns (R) Edge Hill, Cairns [-16.9, 145.7333333], 23.2.1965 Brooks, J.G.” (ANIC); 1 ex., “North Queensland, Townsville [-19.26667023, 146.8166656], 26.4.1968 Ferrar, P.” (ANIC); 1 ex., “North Queensland, 6 1/2 miles S of Townsville [-19.36667, 146.8167], 20.03.1967, Upton, M.S. leg.”(ANIC); 1 ex., “North Queensland Townsville [-19.26667023, 146.8166656], 26.3.1968, Ferrar, P.” (ANIC); 1 ex., “Cape York Pen. Claudie Riv Xing Iron Range, 11.IX.1974 Walford-Huggins” (CLH); 3 exs., “Cape York Pen. Claudie Riv Xing Iron Range, 7.VI.1975 Walford-Huggins” (CLH); 1 ex., “Gum Leaf Lagoon Edward Riv. Mission 21 Nov. 1983 Walford-Huggins” (CLH); 1 ex., “Sarina, NQ Half Tide Beach, 3.ii.73 J. Frost” (CLH); 2 exs., “Australia: S QLD, Tuan State Forest near Poona Crk., water point 9, Scrubby Crk. upstream, 20 m, 29.IX.2006, 25.44.449S 152.51.316E, L. & E. Hendrich leg. (QLD 60)” (CLH); 1 ex., “Australia: C QLD, 10 km S Mizani, Lake Kinchant, seapage, 48 m, 24.IX.2006, 21.11.580S 148.53.522E, L. & E. Hendrich leg. (QLD 46)”, “DNA Balke 1754” [green printed label] (ZSM); 1 ex., “Australia: S QLD, N Brisbane, Caboolture/Beerburrum road, near King John Creek, 29 m, 9.X.2006, 27.03.014S 152.57.021E, L. & E. Hendrich leg. (QLD 62)”, “DNA Balke 2316” [green printed label] (ZSM); 1 ex., “Australia QLD01/19 Mt. Eliott NP 200 m, 4.–6.4.2001, M. Baehr” (ZSM); 5 exs., “Australia, Queensland, Archer River Crossing, 13°25'S, 142°56'E, 7. April 1989, mv lamp, G. & A. Daniels” (QM); 1 ex., “Australia, Queensland, West Claudie River 4 km SW road junction, 27. November 1987, mv lamp, G. Daniels, M.A. Schneider” (QM); 1 ex., “Australia, Queensland, Gordon Creek area, Claudie River Distr., 30. June 1982, M.A. Schneider, G. Daniels” (QM); 1 ex., “Australia, Queensland, 10 km NW Archer River Crossing 13°22'S, 142°54'E, 30. April 1989, mv lamp, G. & A. Daniels” (QM); 2 exs., “Australia, Queensland, Saibai Island, 6. February 1986, 09.23S, 142.40E, at light, Houston, Hamacek” (QDPIB); 1 ex., “Australia, Queensland, St. Pauls Moa Island, 10.–16. Febr. 1986, at light, K. Houston, E. Hamacek” (QDPIB); 1 ex., “Australia, Queensland, CYP Batavia Downs, 12.40S 142.40E, 3. March 1993, 10.III.1993, at light, I. Cunningham” (QDPIB); 2 exs., “Australia, Queensland, Cow Bay N of Daintree, 25.1.–7.2.1984, I. C. Cunningham” (QDPIB); 1 ex., “Australia, Queensland, 15 km WNW of South Johnstone, 7. Jan. 1986, light trap, Halfpapp (QDPIB); 1 ex., “Australia, Queensland, 15 km WNW of South Johnstone, 1.XI.1985, light trap, Fay, Halfpapp” (QDPIB); 2 exs., “Australia, Queensland, Mossman River, 5. January 1984, light trap, J.D. Brown” (QDPIB); 2 exs., “Australia, Queensland, 23 km E of Mareeba, 29. Jan. 1989, at light, R.I. Storey” (QDPIB); 1 ex., “Australia, Queensland, Walkamin, 20. March 1984, light trap, J.D. Brown” (QDPIB); 1 ex., “Australia, Queensland, Tolga, 14. Feb. 1986, light trap, J.D. Brown” (QDPIB); 2 exs., “Australia Qld., Helenvale, 3. 12. 1988 (UV light), Vr. R. Bejšák lgt.” (NMPC); 1 ex., “N Queensland, 14.1.2000, Kuranda, Sv. Bílý leg.” (NMPC); 2 exs., “N Queensland, 17.1.2000, Laura, Sv. Bílý leg.” (NMPC); 3 exs., “N Queensland, 22.1.2000, Undara, Sv. Bílý leg.” (NMPC).

##### Additional material studied from New Guinea (80 specimens).

***Indonesia*: *Papua***: 36 males, 43 females, “Indonesia: Papua, Merauke, Wasur, pools, 20 m, 15–16.x.2011 UNCEN (PAP02)” (MZB, NMW, ZSM). ***Papua New Guinea***: 1 male, “Papua New Guinea: Western Province, Balimo, 0 m, 9.–11.xi.2008, 08.01.823S 142.57.458E, Posman (PNG185)”, “DNA M.Balke 3806” [green printed label] (ZSM).

##### Description of male.

***Body shape:*** In dorsal view oblong oval, broadest at midlength of elytra, moderately convex. Body outline, without discontinuity between pronotum and elytra. Head relatively broad; anterior margin of clypeus not bordered. Pronotum broadest between posterior angles, lateral margins moderately curved. Base of elytra as broad as pronotal base; lateral margins of elytra moderately curved (Fig. 2).

***Colouration:*** Body black, except around eyes clypeus ferruginous; sides of pronotum and elytra basally and laterally (including epipleura) ferruginous; appendages testaceous (Fig. 2).

***Dorsal surface sculpture:*** Whole surface shiny (Fig. 2). Head uniformly microreticulated, reticulation weakly impressed with very small meshes. Densely, weakly and minutely punctate; rows of coarse punctures present around inner margin of eyes and in small depression anterolaterally of eyes. Pronotum with lateral beading very thin and indistinct. Microreticulation and punctation similar to that of head; row of coarse punctures present along anterior margin, basal margin (except basomedially), and laterally close to sides. Elytra with microreticulation similar to that of head and pronotum, but less impressed. Punctation consisting of very fine sparse punctures. Apex of elytra with some large punctures. Each elytron with eight impressed discal and one submarginal longitudinal striae; stria 2 weakly impressed, reduced to a few elongated short striae; stria 1 well separated from suture and in position of innermost row of serial punctures, striae 4 and 6 shorter than striae 5, 7, and 8.

***Antennae and legs:*** Antenna with antennomeres long and slender. Protibia modified, angled near base, slightly broadened anteriorly. Pro- and mesotarsomeres 1–3 distinctly broadened, with adhesive discs on their ventral side; claws simple.

***Ventral part:*** Finely microreticulated, with sparsely distributed, very small punctures. Prosternum obtusely keeled medially. Prosternal process strongly convex, apex bluntly pointed; distinctly bordered laterally. Lateral parts of metaventrite tongue-shaped, very slender. Metacoxal lines close, deep, almost complete and absent only close to metaventrite, and evenly and slightly diverging anteriorly. Metacoxae without striae, but abdominal ventrites 1–4 with numerous longitudinal striae.

***Male genitalia:*** Median lobe consisting of a few sclerites, well separated apically (Fig. 12A, B, C). Shape of paramere narrowly triangular, with very dense, strong, long setae along dorsal margin (Fig. 12D).

##### Female.

Similar to male in habitus. Protibia simple, not angled basally and only slightly broadened distally; pro- and mesotarsomeres not broadened, without adhesive setae.

##### Measurements.

TL = 7.2–8.0 mm; TL-H = 6.5–7.15 mm; MW = 3.4–3.5 mm.

##### Variability.

All specimens studied are rather uniform but can vary in body length. In approximately half of the studied specimens, stria 2 on elytra is not interrupted and reduced to a few elongated short striae. There is a slight variation in the extension of the ferruginous basal band on elytra.

##### Differential diagnosis.

The species can be separated from all other Australian *Copelatus* with more than six striae on elytron by the broad distance between the elytral suture and stria 1 (Watts 1978) and the shape of the median lobe. *Copelatus clarki* is the largest species of the genus in Australia.

##### Distribution.

The species is widely distributed in the northern half of Australia. Records are from north-western Australia (Kimberley region), Northern Territories and Queensland south to Brisbane and Stradbroke Island (Fig. 22).

In this study, the species is recorded for the first time from southern New Guinea (Indonesia: Papua Province, Merauke Regency and Papua New Guinea: Western Province).

##### Habitat.

A widely distributed species in tropical northern Australia, *C. clarki* can be found in almost all lentic habitat types, in open country as well as forested areas (Larson 1993). Most specimens were obtained from isolated pools of seasonal creeks and streams, and pools adjacent to streams in Eucalypt or tropical woodland, filled from leaf and debris (Fig. 31A). During dry periods, the species occurred in high densities in shallow water under dense emergent grasses adjacent to the water’s edge in irrigation reservoirs and dugouts (Larson 1993). On Stradbroke Island, the species was collected in the shallow water of a seasonal *Baumea* sedge swamp (Fig. 31B). The species is also attracted to light.

#### Copelatus daemeli

Taxon classificationAnimaliaColeopteraDytiscidae

Sharp, 1882

6BEF176F-FD65-561D-AD87-516E2B34AD7D

**Figure 3.**
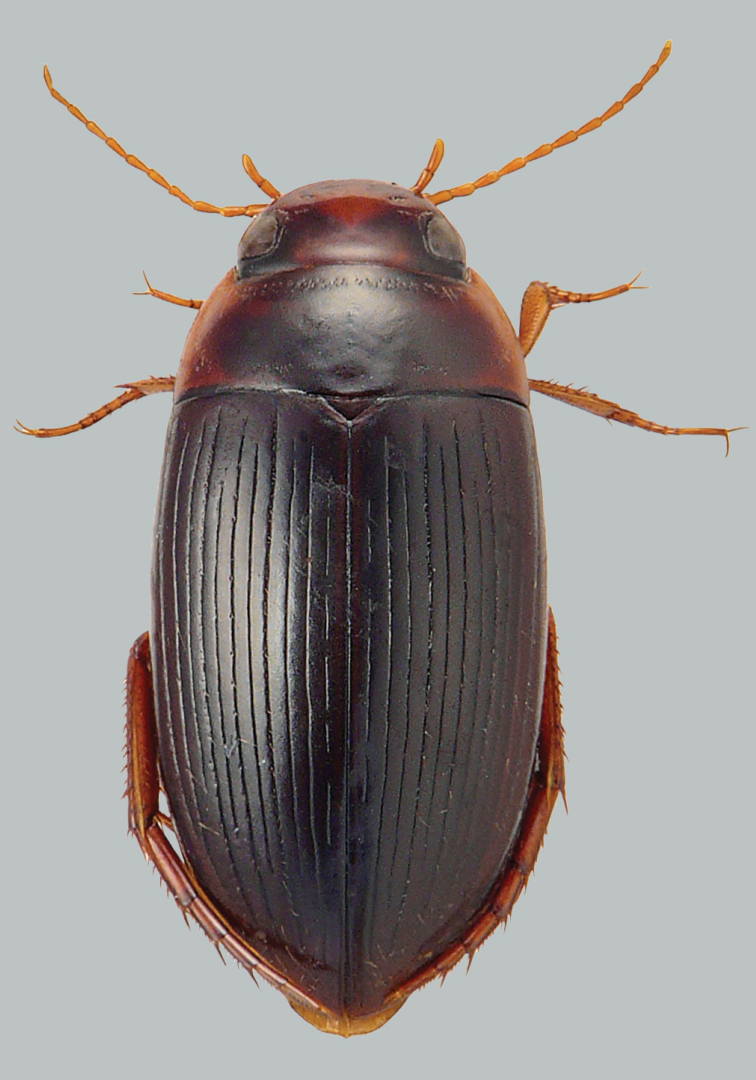
Habitus and colouration of *Copelatus daemeli*, female with 11 fully developed elytral striae. Total length 6.3 mm.

**Figure 4.**
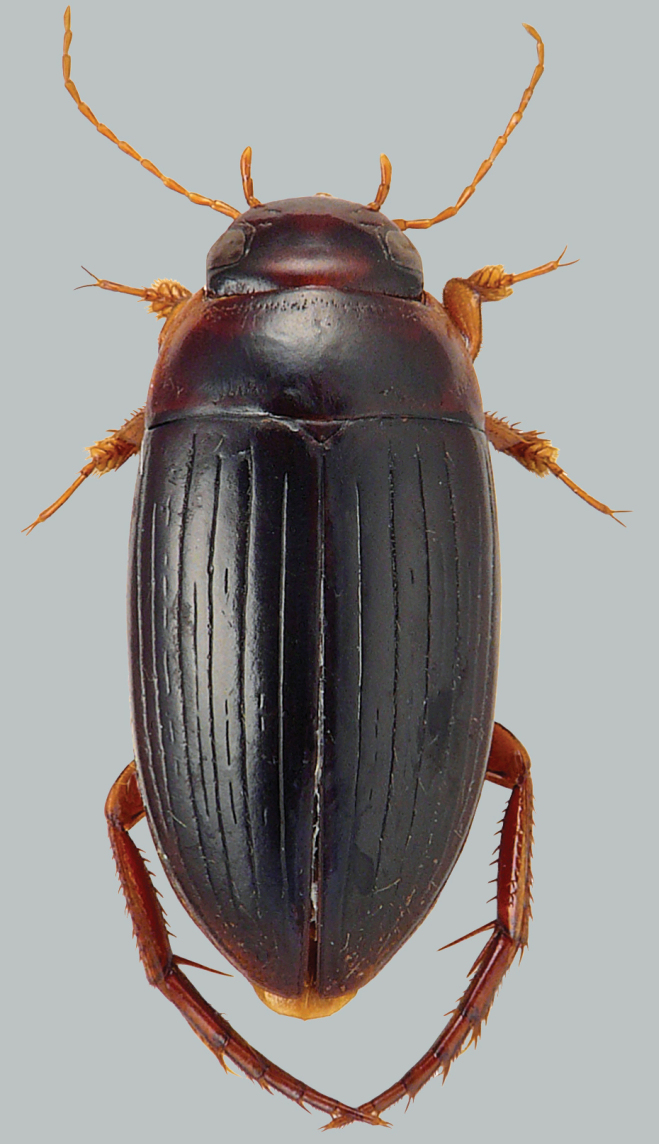
Habitus and colouration of *Copelatus daemeli*, male with reduced elytral striae. Total length 6.2 mm.

Figures 3 , 4 , 13 , 21 , 32

Copelatus daemeli Sharp 1882: 593 (original description); Larson 1993: 52 (habitat information); Watts 1978: 126 (description); Watts 1985: 26 (general distribution); Watts 2002: 42 (checklist); Nilsson and Hájek 2019: 62 (catalogue).

##### Type locality.

“Australia [Queensland] (Cape York)”.

##### Type material.

We were not able to find the type material of *C. daemeli*, neither in Muséum national d’Histoire naturelle, Paris (MNHN) nor in the NHMUK. *Copelatus daemeli* is originally a manuscript name of Wehncke. The depository of many of Wehncke’s types is unknown, but those which were found and studied are mostly stored in the MNHN. The identity of the species is quite clear and it cannot be confused with any other Australian (or New Guinean) species, therefore the designation of a neotype is not necessary and simply refers to undoubted identity of the species in the revision of Watts (1978).

##### Material studied (40 specimens).

***Western Australia:*** 1 ex., “Wyndham-East Kimberley, Mitchell Plateau [-14.6667, 125.7333] 23/9/1982 BV Timms”, “25001777” (SAMA); 1 ex., “AUSTRALIA/WA/ Shire of Wyndham-East Kimberley, Gibb River Road, King Edward River Crossing, 280 m, 15.6.1999, Hendrich leg./Coll./Loc. 10/110” (CLH). ***Northern Territory***: 1 ex., “Australia: NT, Finnis River 10 km W Batchelor, 43 m, 20.VIII.2006, 13.01.278S 130.57.217E, L. & E. Hendrich leg. (NT 2)”, “DNA M. Balke 2523” [green printed label] (ZSM); 1 ex., “Australia: NT, Litchfield NP, Greenant Creek E Tolmer Falls, 51 m, 21.VIII.2006, 13.12.126S 130.42.173E, L. & E. Hendrich leg. (NT 6)”, “DNA M. Balke 1607” [green printed label] (ZSM); 1 ex., “Australia: NT, Kakadu NP, small creek on the road to Gunlom, 101 m, 25.VIII.2006, 13.30.422S 132.26.191E, L. & E. Hendrich leg. (NT 16)”, “DNA M. Balke 1603” [green printed label] (ZSM); 6 exs., “Australia, NT, Litchfield NP, Florence Falls, 12°51'15"S, 132°45'16"E, 63 m, at light, 12.V.2006, leg. Berger & Dostal (5/06)” (CGH); 1 ex., “Australia, NT, Darwin, 12°51'24"S, 131°46'48"E, 52 m, at light, leg. Berger & Dostal (2/06)” (CGW); 8 exs., “Australia NT/ Old Stuart Hwy, Scenic Drive, Robin Falls, Creek, 50 m, 7.7.1999, Hendrich leg. Loc. 37/137” (CLH); 1 ex., “Australia NT/Litchfield N.P., Florence Falls Camping Area, 120 m, 4.11.1996 13°06.705'S, 130°47.220'E L. Hendrich leg./Lok. 16” (CLH); 1 male, “Australia, N.T./Kakadu N.P., Gunlom Camp. Area, pool in Monsoonal Forest, ca. 50 m, 3.11.1996, 13°26.082'S, 132°24.929'E L. Hendrich leg./Lok. 15” (NMW); 1 ex., “Australia, N.T./Kakadu N.P., Mary River District, Gunlom Camping Area, 50 m, 3.11.1996, 13°26.082'S, 132°24.929'E L. Hendrich leg./Lok. 15” (CLH); 1 ex., “Australia, Northern Territory, Tiwi Islands (S), Tiwi Cobourg, Melville Island, [-11.41666985, 131.5166626], 4.2.1968, Matthews, E. leg.” (ANIC); 1 ex., “NT Darwin [-12.4500, 130.8331] 13/5/1963 CHS Watts”, “25-008438” (SAMA); 2 exs., “Australia NT Darwin Holmes Jungle Pk uv lt Dec2/93 S Peck” (ZSM). ***Queensland***: 1 ex., “Australia, QLD, Cape York Peninsula, Lockerbie [-10.8, 142.4667] 31.3.1964, Common, I.F.B. & Upton, M.S. leg.” (ANIC); 1 ex., “Australia, Queensland, Iron Range Cape York Pen., 26. May 1971-2. June 1971, B.K. Cantrell” (QM); 1 ex., “QLD, Townsville [-19.2, 146.68], 6-11/2/ 1998, AJ Watts”, “25-001789” (SAMA); 3 exs., “QLD, Heathlands, Cook [-11.75, 142.58], at light 16/03/1994 Zborowski, P.”, “25-023758-978” (ANIC); 4 exs., “QLD, Heathlands, Cook Pappan Creek [-12.65, 142.01], at light 18/02/1994 Zborowski, P.”, “25-023756-974” (ANIC); 2 exs., “QLD Cook Iron Range [-12.73332977, 143.2832947] 11/05/1971 JG Brooks”, “25-019347-156” (ANIC); 1 ex., “QLD Burster Creek [-10.93333, 142.3833], open forest, at light, 17/10/1992 Weir, T.A. & Zborowski, P.”, “25-019372-244” (ANIC).

##### Description of male.

***Body shape:*** In dorsal view, narrowly elongate, broadest at midlength of elytra. Body outline with small discontinuity between pronotum and elytra. Head relatively broad; anterior margin of clypeus not bordered. Pronotum broadest in middle, lateral margins moderately curved. Base of elytra as broad as pronotal base; lateral margins of elytra moderately curved (Fig. 4).

***Colouration:*** Body dark brown to black, clypeus and sides of pronotum dark ferruginous; appendages testaceous (Fig. 4).

***Dorsal surface sculpture:*** Whole surface shiny (Fig. 4). Head uniformly microreticulated, reticulation weakly impressed with very small meshes. Densely, weakly and minutely punctate; rows of coarse punctures present around inner margin of eyes and in small depression anterolaterally of eyes. Pronotum with lateral beading very thin and indistinct. Microreticulation similar to that of head. Punctation similar to that of head; row of coarse punctures present along anterior margin, basal margin (except for basomedially), and laterally close to sides. Elytra with microreticulation similar to that of head and pronotum, but less impressed. Serial punctures very indistinct, located in striae 4, 6, 8 and 10. Each elytron with ten discal and one well marked submarginal longitudinal stria, alternate striae shorter apically and with a tendency to be interrupted basally. Striae 3 and 5 reduced to a few short grooves and stria 2 broken up basally. Submarginal stria reaching from middle of elytra almost to apical end of stria 10.

***Antennae and legs:*** Antenna with antennomeres long and slender. Protibia modified, angled near base, slightly broadened anteriorly. Pro- and mesotarsomeres 1–3 distinctly broadened, with adhesive discs on their ventral side; claws simple.

***Ventral part:*** Finely microreticulated, with sparsely distributed, very small punctures. Prosternum obtusely keeled medially. Prosternal process quite strong, convex, narrowly flanged and bluntly pointed. Lateral parts of metaventrite tongue-shaped, very slender. Metacoxal lines deep, close apically, evenly and slightly diverging anteriorly. Metacoxae with short sharp striae and abdominal ventrites 1–3 with larger but weaker longitudinal striae.

***Male genitalia:*** Median lobe consisting of a few sclerites, well separated apically (Fig. 13A, B, C). Shape of paramere narrowly triangular, with very dense, strong, long setae along dorsal margin (Fig. 13D).

##### Female.

Similar to male in habitus. Protibia simple, not angled basally and only slightly broadened distally; pro- and mesotarsomeres not broadened, without adhesive setae.

##### Measurements.

TL = 6.2–6.3 mm; TL-H = 5.5–5.6 mm; MW = 2.9–2.95 mm.

##### Variability.

A dimorphic species. Despite the fact that all specimens studied are rather uniform in habitus and colouration, they vary in extension and number of their elytral striae. Several specimens of both sexes, collected at the same spot at the same time with the main form (NT, Robin Falls), have 11 fully developed elytral striae. (Fig. 3).

##### Differential diagnosis.

On the first view *C. daemeli* (especially the form with 11 elytral striae) resembles *C. bakewelli* but differs in the lack of short striae on the pronotum, the larger size, and the less developed and often shortened and reduced elytral striae in most of the specimens. Furthermore, both species can easily be separated by the form of the median lobe.

##### Distribution.

Endemic. The species is distributed from the Kimberley region in Western Australia, over Northern Territory (Melville Island, Kakadu Area and around Darwin) to coastal Queensland (Cape York Peninsula) south to Townsville (Fig. 21). Always rare and collected only in low numbers.

##### Habitat.

The habitat of *C. daemeli* is not well-known. The few specimens have been collected mainly in seasonal habitats, preferably in forested areas. Several specimens were obtained from isolated pools of intermittent creeks and streams, and pools adjacent to streams in eucalypt or tropical woodland (Fig. 32A, B). In Queensland Larson (1993) found a few specimens in a small, silty drying water hole in the otherwise dry bed of a small intermittent stream. Few specimens were taken from a slow flowing spring-fed stream. The species is also attracted to light.

#### Copelatus irregularis

Taxon classificationAnimaliaColeopteraDytiscidae

W.J. Macleay, 1871

DD105FE9-1782-58F5-8159-8A383B009095

**Figure 5.**
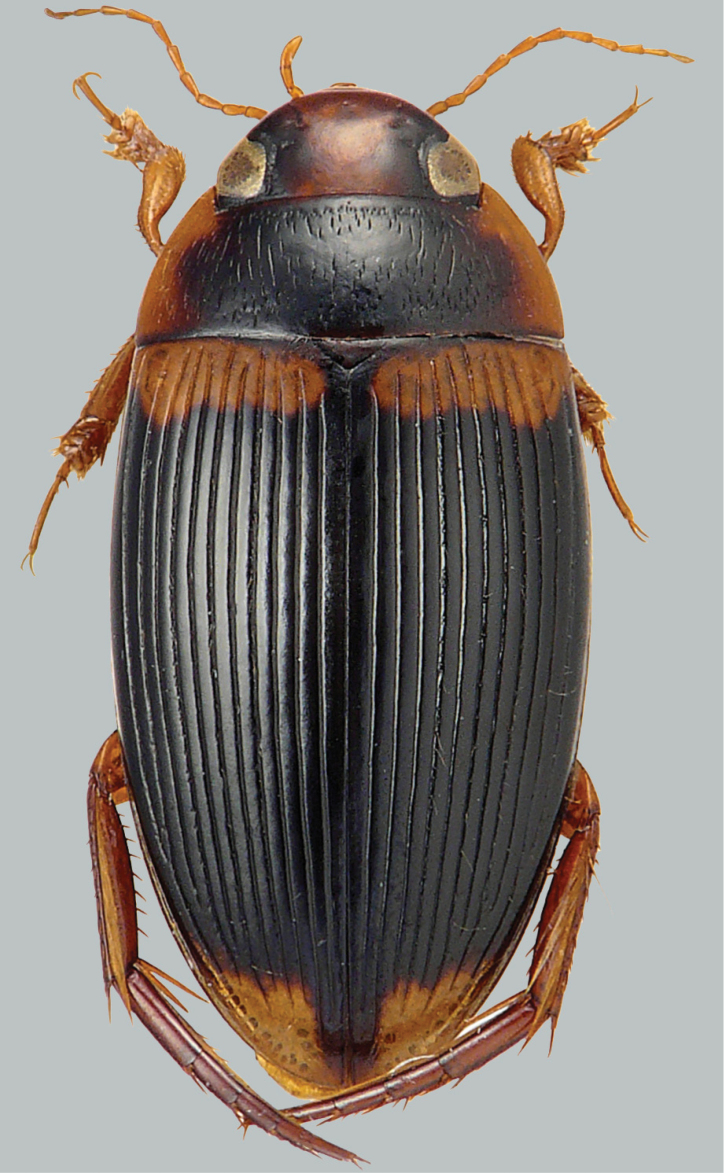
Habitus and colouration of *Copelatus irregularis*, male. Total length 7.7 mm.

Figures 5 , 14 , 23 , 31 , 34

Copelatus irregularis W.J. Macleay 1871: 126 (original description); Watts 1978: 126 (description); Watts 1985: 26 (general distribution); Larson 1993: 52 (habitat information); Larson 1997: 275 (habitat information); Hendrich 2003: 51 (new records); Watts 2002: 42 (checklist); Nilsson and Hájek 2019: 62 (catalogue).

##### Type locality.

“[Australia, Queensland] Lizard Island”.

##### Type material.

ANIC, studied but the exact data not noted.

##### Material studied (221 specimens).

***Western Australia:*** 8 exs., “WA, E-Pilbara, Weeli Wolli Springs, NW of Newman [555 m], 22°54.886'S, 119°12.661'E, 2005, C.H.S. Watts leg.”, “DNA Balke 2655”, “DNA Balke 2656” [green printed labels] (ZSM); 3 exs., “AUSTRALIA/WA/Shire of Wyndham – East Kimberley, Gibb River Raod, Barnett River Gorge, 16°32'15.17"S, 126°7'30.43"E, 450 m, 19.6.1999, Hendrich leg. Loc. 17/117” (CLH); 7 exs., “WA Palm Springs 9/09/2001, JSC” (DPAW); 4 exs., “WA Warrie Springs 8/09/2001 MDS” (DPAW); 4 exs., “AUSTRALIA WA, Mt Fanny 08/07/1969, Giles 50 mi 180, 25°47'S, 128°35'E, Giuliani D.D. leg.” (WAM); 3 exs., “AUSTRALIA WA, Synnot Range 01/09/1969, 16°25'S, 125°28'E, Giuliani D.D. leg.” (WAM); 1 ex., “AUSTRALIA/WA, Gill Pinnacle, 10/07/1969, 24°54'S, 128°47'E, Giuliani D.D. leg.” (WAM); 2 exs., “AUSTRALIA/WA, Charnley River, 25 mi E Beverley Springs, 09/09/1969, 16°15'S, 125°26'E, Giuliani D.D. leg.” (WAM); 4 exs., “AUSTRALIA/ WA, Nullagine [-21.8833, 120.1166] 19.–20.1.1974, Douglas A.M. & M.J. leg.” (WAM); 2 exs., “AUSTRALIA/WA, Marandoo Camp, at light, 22°38'S, 118°06'E, 5.–19.5.1980, Houston, T.F. et al. leg.” (WAM); 1 ex., “AUSTRALIA/WA, Lyons River, Mt Augustus, at light, 24°19'12"S, 116°49'48"E, 2.–3-IX.1980, Howard, C.A. & Houston, T.F. leg.” (WAM); 1 ex., “Western Australia, Pilbara, Ashburton (S), Millstream, Deep Reach [-21.58333, 117.0667], 8.11.1970, Britton, E.B.” (ANIC); 1 ex., “Australia, WA, Pilbara, Hamersley Range, 17 km S Auski Roadhouse, Fig. Tree Crossing 400 m, 28.8.2002, 22°32'S, 118°44'E, Hendrich leg. (Loc. WA 9/173)” (CLH); 2 exs., “Australia, WA, Pilbara, Hamersley Range, Karijini N.P., Dales Gorge [Fortescue Falls and Circular Pool], 400 m, 28.8.2002, 22°29'S, 118°35'E, Hendrich leg. (Loc. WA 10/174)” (CLH); 2 exs., “Australia, WA, Pilbara, Hamersley Range, Karijini N.P., Kalamina Gorge, 450 m, 29.8.2002, 22°25'S, 118°23'E, Hendrich leg. (Loc. WA 11/175)” (CLH); 1 ex., “Australia, WA, Pilbara, Hamersley Range, Karijini N.P., Knox Gorge, 450 m, 29.8.2002, 22°21'S, 118°18'E, Hendrich leg. (Loc. WA 12/176)” (CLH). 1 ex., “W AUSTRALIA, E-Pilbara, Weeli Wolli Springs, 22°54.886'S, 119°12.661'E, 555 m, xi.2001, M. Häckel leg.” (NMPC). ***Northern Territory***: 12 exs., “Australia NT, Trephina Gorge, East MacDonnell Ranges [-23.529, 134.375], October 1972, leg. M. Baehr” (CLH, ZSM); 1 ex., “Australia, NT, Litchfield NP, Florence Falls, 12°51'15"S, 132°45'16"E, 63 m, at light, 12.V.2006, leg. Berger & Dostal (5/06)” (CGW); 2 exs., “Australia, NT, 80 km W Roper Bar, 14°54'19"S, 133°57'28"E, 78 m, at light, 14.V.2006, leg. Berger & Dostal (7/06)” (CGW); 1 ex., “Australia, NT, 40 km v. Port Roper, 14°54'04.8"S, 135°03'24.7"E, 134 m, 15.V.2006, leg. Berger & Dostal” (CGW); 1 male, “Australia, N.T./Kakadu N.P., Jim Jim District, Barramundie Gorge, Maguk, 50 m, 31.10.1996, 13°18.823'S, 132°26.198'E. L. Hendrich leg./ Lok. 9” (NMW); 8 males, 4 females, “Australia, N.T./Kakadu N.P., Gunlom Camp. Area, pool in Monsoonal Forest, ca. 50 m, 3.11.1996, 13°26.082'S, 132°24.929'E L. Hendrich leg./Lok. 15” (NMW); 1 male “Thorey 1867” [partly hw], “Nov: Holl: bor:” [hw], “Coll. Mus. Vindob.” (NHW). 2 exs., “Australia: NT, Litchfield NP, TJAYNERA FALLS, 13°15'S, 130°44'E, 63 m, S. Jákl leg., 20–27.XI.2008” (NMPC); 1 ex., “Australia: NT, Victoria Highway, 110 km to KUNUNURRA, 15°57'S, 129°33'E, 76 m, S. Jákl leg., 30.XI.2008” (NMPC); 1 ex., “Australia: NT, Nitmiluk NP, Edit Falls, 14°10'S, 132°06'E, 37 m, S. Jákl leg., 3.XII.2008” (NMPC); 1 ex., “Australia: NT, 70 km SW of Mataranka, 15°1'S, 132°50'E, 190 m, S. Jákl leg., 22.–23.XII.2008” (NMPC); 2 exs., “Australia NT, 25 km S of Katherine, 168 m, 14°31'S, 132°25'E, 16.–17.1.2009, Sv. Bílý leg.” (NMPC). ***Queensland***: 2 exs., “AUSTRALIA, QLD. Mulgrave R, nr The Fisheries Nov. 9/90 Larson” (ZSM); 3 exs., “AUSTRALIA QLD. McLeod R nr base Windsor Tableland Nov. 12/90 Larson” (ZSM); 4 exs., “Australia S QLD, N Stradbroke Island, creek @Trans-island Rd. 1 km SE Brown Lake, 18.X.2014, 27°30'8.43"S, 153°26'10.78"E, Lars Hendrich leg. (QLD2/14)” (NMW); 1 ex., “QL, 35–47 km SWW Mt. Gamet, 31.I.-4.II.2012, leg. S. Prebsl” (CGW); 11 exs., “Australia, QL, Ravenshoe, 90 km W Innisfail, 900 m, 20.I.1993, leg. G. Wewalka (16)” (CGW); 18 exs., “Australia, QL, Greenvale, 150 km NW Charters Towers, 600 m, 19.I.1993, leg. G. Wewalka (12–14)” (CGW); 6 exs., “Australia QLD/30 km NNW Mareeba, near Mitchell Lake, 9.11. 1996 Hendrich leg./Lok. 20” (CLH); 6 exs., „Australia: S QLD, 40 km E Bundaberg, Tusky Creek, 9 m, 26.IX.2006, 24.39.139S 152.01.477E, L. & E. Hendrich leg. (QLD 52)” (ZSM); 6 exs., “Australia: C QLD, 18 km S Calen, Mt. Charlton-Calen Road, Boulder Creek, 42 m, 23.IX.2006, 21.00.365S 148.43.231E, L. & E. Hendrich leg. (QLD 45)” (ZSM); 3 exs., “Australia: C QLD, 10 km S Mizani, Lake Kinchant, seapage, 48 m, 24.IX.2006, 21.11.580S 148.53.522E, L. & E. Hendrich leg. (QLD 46)” (ZSM); 11 exs., “Australia: C QLD, Paluma Road 4 km W Bruce Hwy, Maiden Hair Fern Crk., 270 m, 20.IX.2006, 19.00.162S 146.17.070E, L. & E. Hendrich leg. (QLD 40)”, 4 specimens with “DNA Balke 1863”, “DNA Balke 1864”, “DNA Balke 2140”, “DNA Balke 2141” [green printed labels] (CLH, ZSM); 2 exs., “Australia: S QLD, Winfield, Winfield Road, forest pool, 21 m, 26.IX.2006, 24.34.084S 152.00.513E, L. & E. Hendrich leg. (QLD 54)” (CLH); 1 ex., “Queensland, Pt. Denison [Port Denison, now Bowen -20.05, 148.25]” (ANIC); 1 ex., “Queensland, North Burnett (R) Gayndah, [-25.63333333, 151.6]” (ANIC); 1 ex., “Queensland Tablelands, N side of Lake Tinaroo, 9.11.1966 [-17.16667, 145.55], Britton, E.B.” (ANIC). 1 ex., “Queensland, Tablelands, Mt. Lewis, tin working site, 3.12.1968 [-16.58333015, 145.2832947], Britton, E.B. & Misko, S.” (ANIC); 1 ex., “Queensland, Townsville (C), Mt. Spec, 5.1.1965, -18.95, 146.1833333, Brooks, J.G.” (ANIC); 1 ex., “Queensland Rockhampton (R) Pistol Gap, Byfield, 10.1.1970 [-22.83333333, 150.6666667], Britton, Holloway & Misko” (ANIC); 1 ex., “Queensland, 9 miles W of Paluma, Charters Towers (R), 15.4.1969 [-19.1, 146.0667], Common, I.F.B. & Upton, M.S.” (ANIC); 1 ex., “North Queensland, Magnetic Island [-19.13333, 146.8333], Lea, A.M.” (ANIC); 1 ex., “Queensland Tablelands (R) Mary Creek [-16.55, 145.2], 4.12.1968 Britton, E.B. & Misko, S.” (ANIC); 1 ex., “Queensland, Townsville (general), 26.3.1968 [-19.26667023, 146.8166656] Ferrar, P.” (ANIC); 1 ex., “Queensland, Cardstone, 16.12.1965 [-17.76666667, 145.5833333] Hyde, K.” (ANIC); 1 ex., “Queensland, Townsville (general) [-19.26667023, 146.8166656], 21.12.1967, Ferrar, P.” (ANIC); 1 ex., “Queensland Tablelands (R), Mary Creek, 5.12.1968 [-16.55, 145.2] Britton, E.B. & Misko, S.” (ANIC); 1 ex., “QLD, Magnetic Island [-19.13, 146.83], A.M. Lea”, “K.215158” (AMS); 1 ex., “Queensland [-22.82407, 147.63635] K.K. Spence”, “K.215159” (AMS); 1 ex., “Queensland [-23.37805, 150.51361] T.G. Sloane”, “K.215160” (AMS); 1 ex., “Queensland Magnetic Island [-19.13, 146.83] A.M. Lea”, “K.215161” (AMS); 1 ex., “Queensland Hayman Island [-20.0508, 148.88783] Jan 1935, F.A. McNeil”, “K.215162” (AMS); 1 ex., “Australia Queensland [-22.82407, 147.63635], “K.215163” (AMS); 1 ex., “Australia Queensland, The Rock Pool, Carnarvon NP [-25.06756, 148.246259] 26 Apr 2006, at MV lamp, D.R. Britton, J.R. Weiner, “K.363072” (AMS); 1 ex., “Australia, Queensland, Tewah Creek via Tin Can Bay, 17. October 1970, T. Weir” (QM); 1 ex., “Australia, Queensland, Baldy Mt. Road, 6 miles SW Atherton, 1100 m, 27. December 1972, B.K.Cantrell” (QM); 1 ex., “Australia, Queensland, Bald Hills Station 4 km N of Isabella Falls, 15°15' 145°00', 29. December 1984, mv lamp, G. & A. Daniels” (QM); 2 exs., “Australia, Queensland, Kenilworth State Forest, via Kenilworth, 20. October 1972, B.K. & J.A. Cantrall” (QM); 4 exs. “Australia, Queensland, Davies Creek via Mareeba, 27. Nov. 1981, R.I. Storey” (QDPIB); 2 exs., “Australia Queensland, Walkamin, 20. March 1984, light trap, J.D. Brown” (QDPIB); 1 ex., “Australia, Queensland Danbulla S.F. via Yungaburra, 13. February 1992, at light, Storey, De Faveri & Huwer” (QDPIB); 1 ex., “Australia, Queensland, 10. Dec. 1985, 15 km WNW of South Johnstone, light trap, Fay & Halfpapp” (QDPIB); 1 ex., “Australia, Queensland, 9. IX. 1985, 15 km WNW of South Johnstone, light trap, Fay & Halfpapp” (QDPIB); 4 exs., “Australia, Queensland, 23 km E of Mareeba, 29. January 1989, at light, R.I. Storey” (QDPIB); 2 exs., “Australia, Queensland, Tolga, 10. December 1982, light trap, J.D. Brown” (QDPIB); 1 ex., “Australia, Queensland”, Paluma, 9.–13. January 1989, light trap, rainforest, R.I. Storey (QDPIB); 1 ex., “Australia, Queensland, Windsor Tableland, 16. December 1984, at light, R.I. Storey (QDPIB); 1 ex., “Australia, Queensland, Garradunga, 17. December 1992, J. Hasenpusch” (QDPIB); 1 ex., “Australia, Queensland, Mt. Mulligen Plateau, 700 m, 15.–19. IV. 1985, at light, K.H. Halfpapp” (QDPIB); 1 ex., “Australia, Queensland, Yam Island 24–27 March 1988, at light, Fay, Halfpapp” (QDPIB); 1 ex., “Australia, Queensland, Pinnarendi Station 60 km W of Mt. Garnet, 7. February 1989, D. Heiner” (QDPIB); 3 exs., “N Queensland, 15.1.2000, Davis Creek, Sv. Bílý leg.” (NMPC); 1 ex., “N Queensland, 29.1.–3.2.2000, The Lynd Junction, Sv. Bílý leg.” (NMPC); 6 exs., “N Queensland, 5.2.2000, Ravenshoe, Sv. Bílý leg.” (NMPC); 3 exs., “N Queensland, 13.2.2000, Undara, Sv. Bílý leg.” (NMPC).

##### Doubtful record.

1 ex., “Tasmania, Gladstone, Dorset, A.M. Lea” (SAMA), probably mislabelled.

##### Description of male.

***Body shape:*** In dorsal view, oblong oval, broadest at midlength of elytra, moderately convex. Body outline almost continuous, with slight discontinuity between pronotum and elytra. Head relatively broad; anterior margin of clypeus not bordered. Pronotum broadest just before posterior angles, lateral margins moderately curved. Base of elytra as broad as pronotal base; lateral margins of elytra moderately curved (Fig. 5).

***Colouration:*** Body black, most of clypeus, sides of pronotum, base and tip of elytra, appendages and much of ventral surface testaceous (Fig. 5).

***Dorsal surface sculpture:*** Whole surface shiny (Fig. 5). Head uniformly microreticulated, reticulation composed of moderately deeply impressed isodiametric very small meshes. Punctation composed of very small punctures sparsely spread on surface; rows of deep and coarse punctures present around inner margin of eyes and in small depression anterolaterally of eyes. Pronotum with some moderately strong, short striae, more dense and weak laterally; lateral beading of pronotum very thin and indistinct. Microreticulation and punctation similar to that of head; row of coarse setigerous punctures present along anterior margin, basal margin (except basomedially), and laterally close to sides. Elytra with microreticulation similar to that of head and pronotum, but less impressed. Punctation consisting of very fine sparse punctures. Each elytron with 11 strongly impressed discal and one submarginal longitudinal striae, intervals subequal, alternate striae tending to be shorter apically; submarginal stria reaching from little behind middle of elytron almost to end of stria 10. Serial punctures on elytron untraceable.

***Antennae and legs:*** Antenna with antennomeres long and slender. Protibia modified, distinctly broadened anteriorly (2/3^rd^) and strongly narrowed basally (1/3^rd^). Pro- and mesotarsomeres 1–3 distinctly broadened, with adhesive discs on their ventral side; claws simple.

***Ventral part:*** Finely microreticulated, with intermixed, sparsely distributed, very small punctures. Prosternal process rather flat, distinctly bordered laterally, weakly and bluntly pointed. Lateral parts of metaventrite tongue-shaped, very slender. Metacoxal lines close, well-marked and moderately divergent anteriorly. Metacoxae with some short and deep striae, abdominal ventrites 1–3 with numerous longitudinal striae.

***Male genitalia:*** Median lobe consisting of a few sclerites, well separated apically (Fig. 14A, B, C). Shape of paramere narrowly triangular, with very dense, strong, long setae along dorsal margin (Fig. 14D).

##### Female.

Similar to male in habitus. Pro- and mesotarsomeres not broadened, without adhesive setae. Pronotal striae fine and dense.

##### Measurements.

TL = 6.8–7.8 mm; TL-H = 6.0–6.9 mm; MW = 3.2–3.4 mm.

##### Variability.

All specimens studied are rather uniform in shape and size but vary in the extension of the testaceous elytral markings.

##### Differential diagnosis.

The species is close to *C. bakewelli* but can be easily separated by the larger size, the dorsal colouration, and the form of the median lobe.

##### Distribution.

The species is widely distributed in the northern half of Australia. Records are from the Northern Territory (inland to the East MacDonnell Ranges), north-western Australia (Kimberley region and the Pilbara), and Queensland south to Brisbane (Fig. 23).

According to the literature, *C. irregularis* occurs also in New Guinea, cited in Guignot (1956: 55) and Zimmermann (1920: 140); Papua in Guéorguiev (1968: 9); Papua New Guinea: Central Province: Redscar Bay in Régimbart (1899: 306) and Guéorguiev and Rocchi (1993: 158). However, all these records are in need of confirmation.

##### Habitat.

This is a widely distributed species and one of the most common *Copelatus* in tropical northern Australia. It can be found in almost all habitat types, preferably in forested areas. Most specimens were obtained from shallow water, amongst leaves and plant debris, of isolated pools of seasonal creeks and streams, and pools adjacent to streams in Eucalypt or tropical woodland. Also, a few specimens were taken from slow flowing spring-fed streams. In the Pilbara (Hendrich 2002), *C. irregularis* was found in different isolated and half-shaded rocky and sandy pools (10–20 m², up to 1.5 m depth) of an intermittent stream. The bottom of most habitats consisted of sand and stones, with a thin layer of mud and plant debris (Figs 31A, 34A). *Copelatus irregularis* is also attracted to light.

#### Copelatus marginatus

Taxon classificationAnimaliaColeopteraDytiscidae

Sharp, 1882

8B4B21BB-58A6-5727-A8F1-B41547F93D7B

Figures 6 , 15 , 25

Copelatus marginatus Sharp 1882: 579 (original description); Watts 1978: 124 (description); Watts 1985: 26 (general distribution); Larson 1993: 53 (habitat description); Watts 2002: 42 (checklist); Wewalka et al. 2010: 50–51 (description, new records); Nilsson and Hájek 2019: 58 (catalogue).

##### Type locality.

“Australia, [Queensland], Rockhampton”.

##### Type material.

***Lectotype*** (designated by Watts: 1978: 124): Male, “Lectotype” [printed label with violet frame]; “Rockhampton 675.” [printed label]; “Sharp Coll 1905-313.” [printed label]; “Type 675 Copelatus marginatus Rockhampton” [hw label] (NHMUK). ***Paralectotypes***: 1 male, 5 females, “Paralectotype” [printed label with blue frame]; “Rockhampton 675.” [printed label]; “Sharp Coll 1905-313.” [printed label]; “Type 675 Copelatus marginatus Co-type” [hw label] (NHMUK).

##### Additional material studied (244 specimens).

***Western Australia:*** 1 ex., “Australia07, WA 50, Parrys Lagoon, Marigu Billabong, 25 km SE Wyndham, 77 m, 15.32.98S 128.15.59E, M. Baehr” (ZSM); 1 ex., “AUSTRALIA/WA, Koolan Island [16°09'S, 123°45'E], VIII–IX 1967, Milton O. leg.” (WAM); 1 ex., “AUSTRALIA/WA, Derby [-17.3166, 123.6333], 2.1962, Beamish, G. leg.” (WAM); 1 ex., idem but „10.4.1964” (WAM). ***Northern Territory***: 1 ex., “Australia, N.T. Binis Track 10.IV.2011 LF; H = 427 m 20°51'51.4"S, 135°12'01.6"E leg. Michael Langer” (ZSM); 1 ex., “Australia, NT, 80 km W Roper Bar, 14°54'19"S, 133°57'28"E, 78 m, at light, 14.V.2006, leg. Berger & Dostal (7/06)” (CGW); 1 ex., “Australia, NT, Kakadu NP, Muirella, 40 m, at light, 10.V.2006, leg. Berger & Dostal (3/06)” (CGW); 1 ex., “Katherine (T) Vestjens, 1.12.1967 [-14.51667, 132.3667], W.J.M. Tindal” (ANIC); 1 ex., “Katherine [-14.46667, 132.2667], 9.2.1968, Watson, J.A.L.” (ANIC); 1 ex., “Howard Springs 27.01.1968 [-12.45, 131.05] Watson, J.A.L.” (ANIC); 1 ex., “Northern Territory Katherine [-14.46667, 132.2667], 6.2.1968, Matthews, E.” (ANIC); 1 ex., “Northern Territory, Lee Point, Darwin [-12.33333333, 130.9], 7.3.1967, Upton, M.S.” (ANIC); 1 ex., “Northern Territory, Roper Gulf (S), Mataranka [-14.93333333, 133.0666667], 1.3.1967, Upton, M.S.” (ANIC); 1 ex., “NT Davenport Murchison Ranges, Barkly (S), 15 miles N of Tennant Ck. [-19.4, 134.1833333], 27.2.1967, Upton, M.S.” (ANIC); 1 ex., “NT Brock Creek, Burnside [Brocks Creek] [-13.46667, 131.4167], 27.3.1929, Campbell, T.G.” (ANIC); 1 ex., “Howard Springs, Litchfield (M) [-12.45, 131.05], 27.1.1968, Matthews, E.” (ANIC); 1 ex., “Australia N.T. Kununurra m 50 23.VIII.1996 P.M. Giachino legit” (CLH); 1 ex., “Australia N.T. Umbrawarra Gorge Campground, ca. 40 km S Pine Creek, 27.IV.–18.V.2009, S 13°58'01.4"S, 131°41'57.2"E, 163 m, NF, leg. M. Langer” (CLH); 2 exs.,“Australia: N.T./ ca. 30 km S Daly Waters und ca. 90 km N Elliott am Stuart Hwy 04.IV.2011 LF H = 269 m 16°46'38.8"S, 133°25'53.8"E leg. Michael Langer” (CLH); 11 exs., “Australia: NT, Litchfield NP, TJAYNERA FALLS, 13°15'S, 130°44'E, 63 m, S. Jákl leg., 20–27.XI.2008” (NMPC); 5 exs., “AUSTRALIA: NT, 25 km S of KATHERINE, rd to Kununurra, 14°44'S, 132°01'E, 100 m, S. Jákl leg., 17.–31.XII.2008” (NMPC); 12 exs., “AUSTRALIA: NT, 25 km S of KATHERINE, nr Cutta Cutta caves, 14°31'S, 132°25'E, 168 m, S. Jákl leg., 23.–31.XII.2008” (NMPC); 1 ex., “Australia: NT, Nitmiluk NP, Edit Falls, 14°10'S, 132°06'E, 37 m, S. Jákl leg., 3.XII.2008” (NMPC); 2 exs., “AUSTR. NT, Mataranka, 14°53'S, 132°01'E, 18.12.08, 148 m, Sv. Bílý leg.” (NMPC); 70 exs., “Australia: NT, 70 km SW of Mataranka, 15°19'S, 132°50'E, 190 m, S. Jákl leg., 22.–23.XII.2008” (NMPC); 1 ex., “AUSTRALIA: NT, West Macdonnell Range NP, SIMPSON GAP, 23°40'S, 133°43'E, 600 m, S. Jákl leg., 3.–5.I.2009” (NMPC). ***Queensland***: 1 ex., “Cardstone [-17.76666667, 145.5833333], 16.12.1965, Hyde, K.” (ANIC); 1 ex., “Mary Creek [-16.55, 145.2], 4.12.1968, Britton, E.B. & Misko, S.” (ANIC); 1 ex., “Cairns [-16.91667, 145.7667] Taylor, F.H.” (ANIC); 1 ex., “Queensland, Whitsunday, Mimosa Motel, 3 m. S Bowen [-20.01667, 148.25], 23.3.1967, Brooks, J.G.” (ANIC); 1 ex., “Townsville [-19.26667023, 146.8166656], Taylor, F.H.” (ANIC); 36 exs., “Australia/Queensland 30 km E Normanton riverside at light, 1.1996, S. Lamond leg. Coll. Hendrich” (CLH); 31 exs., “Australia/ Queensland 20 km E of Normanton February 1996, at light, Steven Lamond leg. Coll. L.Hendrich” (CLH); 4 exs., “Bowen N.Q. 23/III/1962 A. & M. Walford-Huggins” (CLH); 10 exs., “Australia, QL, Rollingstone, 20 km NW Townsville, 20 m, 17.I.1993, leg. G. Wewalka (7)” (CGW); 3 exs., “Australia, QL, Dalrymple, 30 km N Charters Towers, 300 m, 18.I.1993, leg. G. Wewalka (10, 11)” (CGW); 1 ex., “QL, 15 km S Ogmore, 24.I.2012, leg. S. Prepsl” (CGW); 3 exs., “Australia, Queensland, Horn Islet, Sir Edward Pellew Group, 15. February 1968-21. February 1964, B. Cantrell” (QM); 1 ex., “Australia, Queensland, Iron Range, Cape York 23. April 1966, G. Monteith” (QM); 2 exs., “Australia, Queensland, CYP Batavia Downs, 12.40S, 142.40E, 3. March 1993-10. March 1993, at light, I. Cunningham” (QDPIB); 6 exs., “Australia, Queensland, Tolga, 26. Jan.–31. Jan. 1981, at light, N. Gough” (QDPIB); 1 ex., “Australia, Queensland, Burketown 31. Jan. 1992, at light, B.M. Waterhouse, J.F. Grimshaw” (QDPIB); 1 ex., “Australia, Queensland Danbulla S.F. via Yungaburra 13. February 1992 at light, Storey, De Faveri & Huwer” (QDPIB); 1 ex., “Australia, Queensland, Normanton, 4. September 1982, at light, R.I. Storey” (QDPIB); 1 ex., “Australia Queensland Kauri Creek, Para Grass, 30. August 1995, B. Hebert” (QDPIB); 1 ex., “Australia, Queensland, Ingham, 6. March 1984 at light, K.H. Halfpapp” (QDPIB); 4 exs., “AUSTRALIA Qld. Windsor Tableland Feb 6-8/91 Larson & Storey” (ZSM); 2 exs., “N Queensland, 14.1.2000, Kuranda, Sv. Bílý leg.” (NMPC); 1 ex., “N Queensland, 9.2.2000, Kuranda, Sv. Bílý leg.” (NMPC); 1 ex., “N Queensland, 7.2.2000, Mt. Carbine, Sv. Bílý leg.” (NMPC); 1 ex., “N Queensland, 13.2.2000, Undara, Sv. Bílý leg.” (NMPC).

##### Description of male.

**Figure 6.**
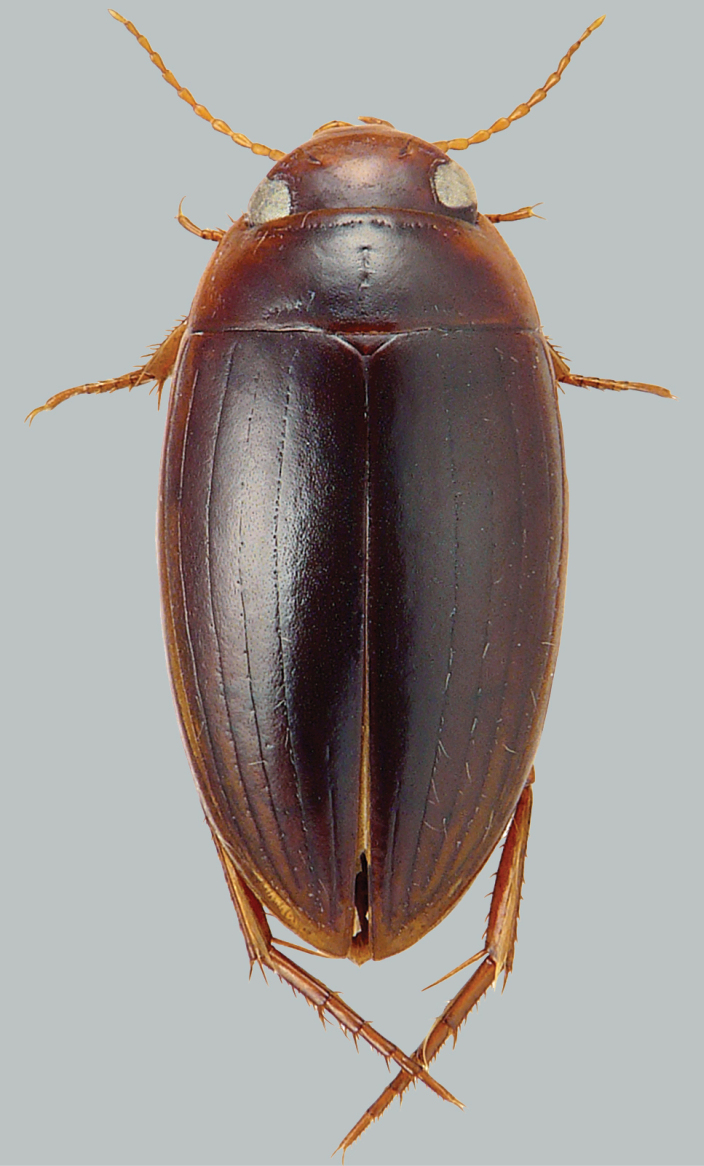
Habitus and colouration of *Copelatus marginatus*, female. Total length 5.8 mm.

***Body shape:*** In dorsal view oval, almost ovoid, broadest in basal third of elytra, moderately convex. Body outline without discontinuity between pronotum and elytra. Head relatively broad; anterior margin of clypeus truncate. Pronotum broadest between posterior angles, lateral margins moderately curved. Base of elytra as broad as pronotal base; lateral margins of elytra moderately curved (Fig. 6).

***Colouration*.** Body dark red-brown to black, clypeus anteriorly, sides of pronotum broadly and elytra laterally (including epipleura) paler, ventral side and appendages testaceous (Fig. 6).

***Dorsal surface sculpture:*** Whole surface almost matt (Fig. 6). Head uniformly microreticulated, reticulation composed of moderately deeply impressed meshes. Punctation composed of coarse setigerous punctures, and very small punctures spreading sparsely on surface; rows of coarse punctures present around inner margin of eyes and in small depression anterolaterally of eyes. Pronotum with lateral beading very thin and indistinct. Microreticulation and punctation similar to that of head; row of coarse setigerous punctures present along anterior margin, basal margin (except for basomedially), and laterally close to sides. Elytra with microreticulation similar to that of head and pronotum, but less impressed. Serial punctures on elytra rather weak. On each elytron six moderately impressed thin discal and one submarginal longitudinal striae, progressively closer towards sides; stria 1 (sutural stria) reduced to apical fourth, striae 2 and 4 complete, striae 3 and 5 a little shorter basally. Submarginal stria short, expanding from behind middle of elytron to end of stria 6.

***Antennae and legs:*** Antenna with antennomeres long and slender. Protibia modified, distinctly broadened anteriorly. Pro- and mesotarsomeres 1–3 distinctly broadened, with adhesive discs on their ventral side; claws simple.

***Ventral part:*** Finely microreticulated, with intermixed, sparsely distributed, very small punctures. Prosternal process shortly lanceolate, apex obtuse; distinctly bordered laterally. Lateral parts of metaventrite tongue-shaped, slender. Metacoxal lines close, evenly and moderately diverging anteriorly, ending short of metasternum. Metacoxae and abdominal ventrites 1–3 with numerous longitudinal or oblique striae.

***Male genitalia:*** Median lobe narrow, simple, tapering and slightly curved downwards apically in lateral view (Fig. 15A, B, C). Shape of paramere broadly triangular, with weak, relatively short setae, mainly along dorsal margin of subdistal part (Fig. 15D).

##### Female.

Similar to male in habitus. Protibia simple, not angled basally and only slightly broadened distally; pro- and mesotarsomeres not broadened, without adhesive setae. Pronotum basolaterally with numerous short longitudinal strioles. Elytra between striae and side with numerous short longitudinal strioles.

##### Measurements.

TL = 5.7–6.0 mm; TL-H = 5.3–5.6 mm; MW = 2.7–2.8 mm.

##### Variability.

All specimens studied are rather uniform and vary only in body length. In some specimens, the submarginal stria is reduced to few elongate grooves.

##### Differential diagnosis.

The species is close to *C. tenebrosus* but can be easily separated by the larger size, much shorter inner striae of elytra, and the form of the median lobe.

##### Distribution.

The species occurs in tropical and subtropical northern and central Australia, along the east coast south to Brisbane (WA, NT, QLD) (Fig. 25). Additionally, *C. marginatus* was recorded from New Caledonia (South Province) and West Samoa by Wewalka et al. (2010). Zimmermann (1927: 17) reported the species from Tonga (two specimens from Tonga: Nukualofa, 22.xi.1925). He wrote also that the presence of the species in Samoa is confirmed by material of Dr K. Friederichs (Friederichs 1922: 148: Samoa, five specimens). Later, *C. marginatus* was reported from Samoa (Upolu), Fiji (Viti Levu and Vanua Levu) and New Guinea (Dorey, now Manokwari, Indonesia: West Papua Province) by Balfour-Browne (1945: 106 and 113) which was repeated by some later authors (Guéorguiev 1968: 21; Guéorguiev and Rocchi 1993: 159; Wewalka et al. 2010: 51). The records from Fiji and New Guinea are in need of confirmation.

##### Habitat.

Most specimens were collected during or just after the rainy season when the beetles can be collected in seasonal flood meadows along rivers and creeks, shallow roadside ditches and swampy areas. At that time of the year, *C. marginatus* is often attracted in larger numbers to light (e.g., Normanton). In north-eastern Queensland, the species was collected in a small roadside pool and in isolated pools in seasonal fingertip tributary streams, all in closed forest (Larson 1993).

#### Copelatus martinbaehri sp. nov.

Taxon classificationAnimaliaColeopteraDytiscidae

4BC8C219-282D-5B35-AA34-375993E0145A

http://zoobank.org/294C8A13-B08A-4815-B6B1-944CEED737EF

Figures 7 , 16 , 28

##### Type locality.

Moreguina (St Stephen’s Mission), Central Province, Papua New Guinea Central, 50 m, 10.018104S, 148.467793E.

##### Type material.

***Holotype:*** Male, “Papua New Guinea Central, Moreguina, 18.viii.2008 Posman (PNG184)” [printed label]; “Holotype *Copelatus martinbaehri* sp.n. Hendrich, Shaverdo, Hajek & Balke des. 2019 [red printed label] (ZSM). ***Paratypes (23 specimens)*: *Australia***: 7 exs., “12.445S, 143.14E QLD 3 km ENE Mt. Tozer 28June–4July 1986 D.H.Colless Malaise Trap” “ANIC Database No. 25 019356” [printed label]; “Aust. Nat. Ins. Coll.” [green printed label] (ANIC, ZSM); 1 male, “N. Queensland IRON RANGE Gordon's CK. [Creek, -12.715780, 143.302092] 10.5.71 at light leg: J.G. Brooks” [hw on both label sides], “COLL. HENDRICH BERLIN” (NMW); 1 male, 1 female, “Gordon's CK [Creek, -12.715780, 143.302092] Iron RA [Range] N.Q. [North Queensland] 100' [100 m] 10.5.71 J.G. Brooks “at light” (CLH); 1 female, “Iron RA N.Q. 4.5.71 J.G. Brooks “at light” (CLH); 2 females, “Iron Range, Cape York Pen., N.-Qld 26 May–2 June 1971 B.K. Cantrell” (QM); 2 males, 2 females, “Iron Range, Cape York Pen. N.Q. 13.–20.V.1975 K.J. Houston At light” (QDPC, ZSM). ***Papua New Guinea***: 1 male, 1 female, same data as holotype, one male additionally with a green label “DNA M.Balke 3803” (ZSM). 2 females, “Papua New Guinea: Central, Moreguina, 16.viii.2008 Posman (PNG183)” [printed label] (ZSM). 1 male, “Papua New Guinea: Northern Kokoda, 410 m, i.2008, 53.4[?]81S 147 43.648E, Posman, (PNG 174)” (ZSM). All paratypes with our red printed labels.

##### Description of male holotype.

**Figure 7.**
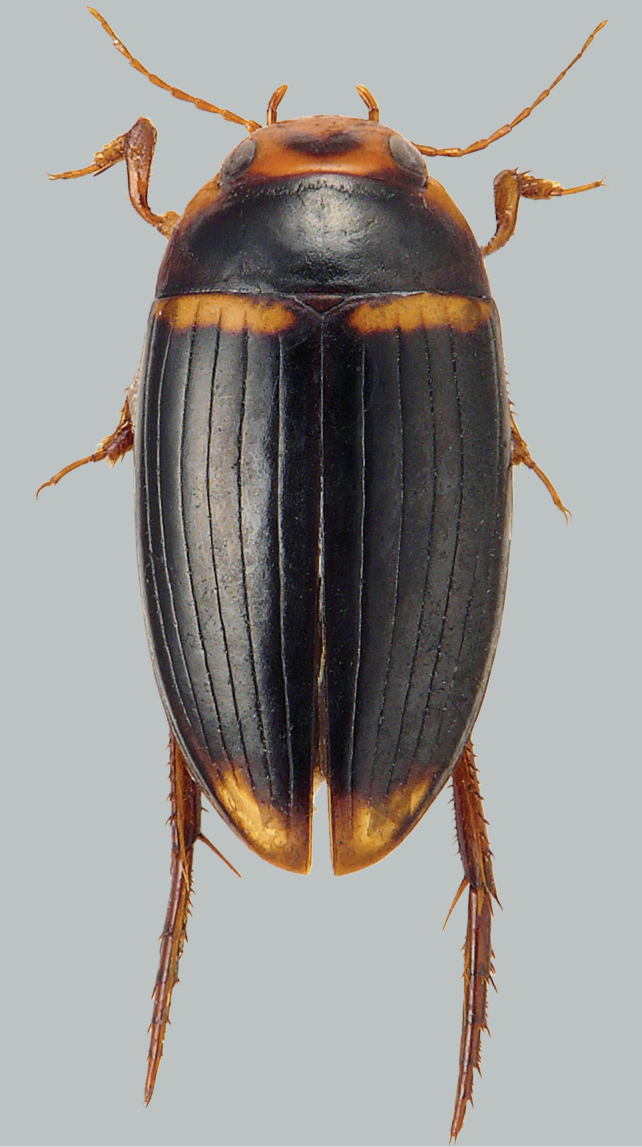
Habitus and colouration of *Copelatus martinbaehri* sp. nov., paratype (Mount Tozer), male. Total length 6.7 mm.

***Body shape:*** In dorsal view, oblong-oval, broadest in basal third of elytra, moderately convex. Body outline without discontinuity between pronotum and elytra. Head relatively broad; anterior margin of clypeus not bordered. Pronotum broadest between posterior angles, lateral margins moderately curved. Base of elytra as broad as pronotal base; lateral margins of elytra moderately curved (Fig. 7).

***Colouration:*** Body black, most of clypeus, anterior angles of pronotum, base and tip of elytra, appendages and much of ventral surface testaceous.

***Dorsal surface sculpture:*** Whole surface shiny (Fig. 7). Head uniformly microreticulated, reticulation composed of moderately deeply impressed isodiametric very small meshes. Punctation composed of very small punctures spread sparsely on surface; rows of deep and coarse punctures present around inner margin of eyes and in small depression anterolaterally of eyes. Pronotum with some weak and short striae laterally. Microreticulation and punctation similar to that of head; row of coarse setigerous punctures present along anterior margin, basal margin (except for basomedially), and laterally close to sides. Elytra with microreticulation similar to that of head and pronotum, but less impressed. Punctation consisting of very fine sparse punctures. Each elytron with six strongly impressed discal and one submarginal longitudinal striae, intervals subequal, striae 1 and 5 tending to be shorter basally; submarginal stria reaching from little behind midlength of elytron almost to end of stria 6. Serial punctures on elytron untraceable.

***Antennae and legs:*** Antenna with antennomeres long and slender. Protibia modified, distinctly broadened anteriorly (2/3^rd^) and strongly narrowed basally (1/3^rd^). Pro- and mesotarsomeres 1–3 slightly broadened, with adhesive discs on their ventral side; claws simple.

***Ventral part:*** Finely microreticulated, with intermixed, sparsely distributed, very small punctures. Prosternal process rather flat, distinctly bordered laterally, weakly and bluntly pointed. Lateral parts of metaventrite tongue-shaped, very slender. Metacoxal lines close, well-marked and moderately divergent anteriorly. Metacoxae with long and deep striae, abdominal ventrite I with numerous striae and ventrites 2–3 with a few longitudinal striae.

***Male genitalia:*** Median lobe sickle-shaped, narrow, simple; in ventral view, appearing somewhat flattened; in lateral view, apically tapering and strongly curved downwards (Fig. 16B, C). Shape of paramere broadly triangular, with weak, relatively short setae, mainly along dorsal margin of subdistal part (Fig. 16D).

##### Female.

Similar to male in habitus. Protibia not modified. Pro- and mesotarsomeres not broadened, without adhesive setae.

##### Measurements.

***Holotype:*** TL = 6.35 mm; TL-H = 5.85 mm; MW = 3.1 mm. ***Paratypes***: TL = 6.2–6.75 mm; TL-H = 5.85–6.1 mm; MW = 3.1–3.2 mm.

##### Variability.

All specimens studied are rather uniform in shape and size but vary a bit in the extension of the testaceous elytral markings.

##### Differential diagnosis.

Based on the characteristic sickle-shaped median lobe, the new species belongs to a difficult complex of species distributed in the Sunda Islands and New Guinea, including *C. geniculatus* Sharp, 1882, *C. gentilis* Sharp, 1882, *C. lineatus* (Guérin-Méneville, 1838) and *C. subterraneus* Guéorguiev, 1978 (of the *C. irinus* species group) and several additional undescribed species, both from the *C. irinus* group (i.e., with six dorsal striae on elytra) and the *C. trilobatus* group (with 11 dorsal striae). All those species are rather uniform in body shape and colouration; they differ in elytral striation (which may be, however, variable) and less so in the shape of the median lobe in lateral view, especially in the width of the medial part and in length and curvature of the apical part. *Copelatus martinbaehri* sp. nov. differs from the other species of this complex by the shape of the median lobe, which has the central part in lateral view broader, but without distinct tubercle on the ventral side; additionally, the apical part is shorter and almost straight (the angle between central and apical part of median lobe in lateral view is nearly rectangular).

Within other Australian species with six elytral striae, *Copelatus martinbaehri* sp. nov. can be easily distinguished by its larger size (*C. tenebrosus* is always less than 5 mm), more elongate habitus (*C. portior* more ovoid, oval), elytral colouration (*C. marginatus* and *C. tenebrosus* with almost black dorsal surface), and the shape of the median lobe.

##### Etymology.

This species is dedicated in honour of our late colleague Dr Martin Baehr (*10.3.1943, †17.4.2019, Munich, Germany), coleopterist, arachnologist, and others as well as the most knowledgeable authority for Australian ground beetles. The specific epithet is a substantive in the genitive case.

##### Distribution.

Northern Queensland (Iron Range National Park at Cape York Peninsula) and south-eastern Papua New Guinea (Central Province) (Fig. 28).

##### Habitat.

Unknown. Most probably, the new species is an inhabitant of temporary lowland rainforest pools. The type specimens were collected in a Malaise Trap and at light.

#### Copelatus nigrolineatus

Taxon classificationAnimaliaColeopteraDytiscidae

Sharp, 1882

087BF126-E5A4-5390-BB17-28742D32DD9F

**Figure 8.**
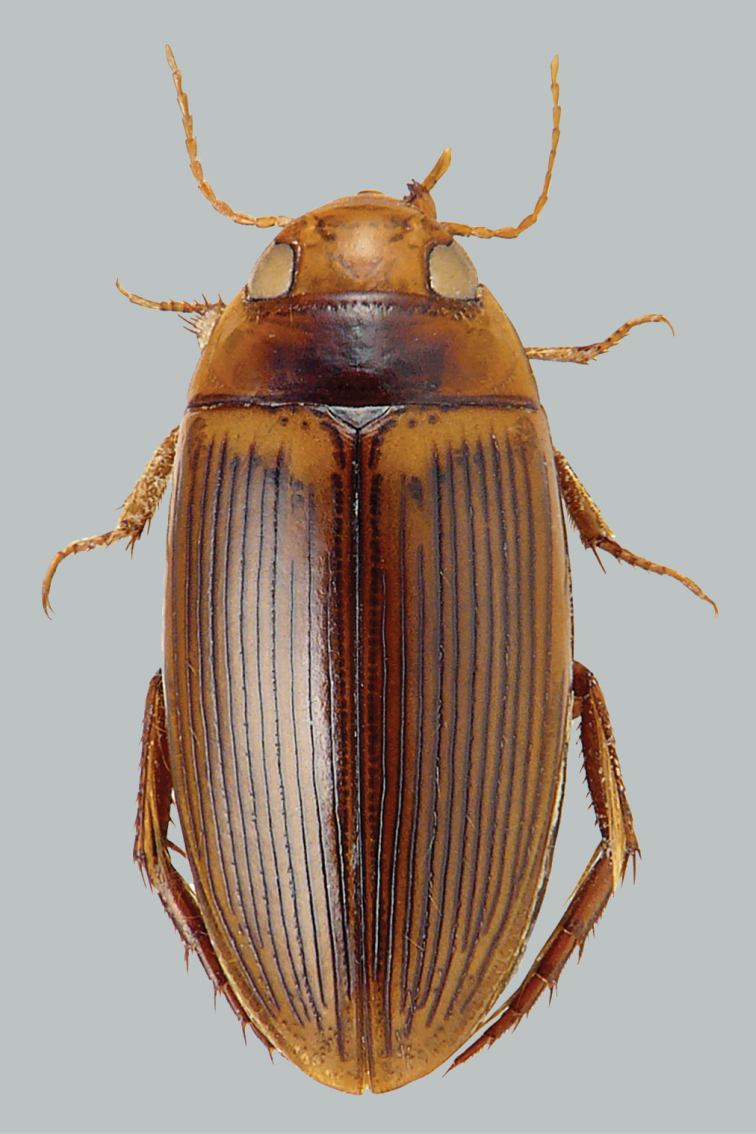
Habitus and colouration of *Copelatus nigrolineatus*, female. Total length 5.7 mm.

Figures 8 , 17 , 26 , 34 , 35

Copelatus nigrolineatus Sharp 1882: 577 (original description); Watts 1978: 122 (description); Watts 1985: 26 (general distribution); Larson 1993: 53 (habitat information); Larson 1997: 272 (habitat information); Hendrich 2003: 51 (new records); Watts 2002: 42 (checklist); Nilsson and Hájek 2019: 62 (catalogue).

##### Type locality.

“Australia, (Champion Bay, Carpentaria, Port Denison, Port Boweii)”.

##### Type material.

***Lectotype*** (designated by Watts 1978: 122): Male, “Lectotype” [round label with violet frame], “11 on each elytron” [hw label], “N.W. Australia. du Bulay” [hw label], “Sharp Coll. 1905-313.” [printed label], “Type 677 Copelatus nigrolineatus n.sp. Australia” [hw label], “Copelatus nigrolineatus Sharp Det. C. Watts 1974” [hw label] (NHMUK). ***Paralecotypes***: 1 male and 3 females, “Paralectotype” [round printed label with blue frame], “Cotype” [roundish label with yellow frame], “Port Denison 677” [hw label], “Sharp Coll. 1905-313” [printed label], “Copelatus nigrolineatus Sharp Cotype” [hw label] (NHMUK).

##### Additional material studied (415 specimens).

***Western Australia:*** 43 exs., “AUSTRALIA/WA/Shire of Wyndham – East Kimberley, Great Northern Hwy, 50 km S Wyndham, Black Flag Creek, 15°52'1.22"S, 128°21'49.97"E, 50 m, 13.6.1999, Hendrich leg. (Loc. 4/104)” (CLH, ZSM); 10 exs., “AUSTRALIA/WA/Shire of Wyndham – East Kimberley, Gibb River Road, Durack River Crossing, 15°56'28.73"S, 127°13'9.01"E, 250 m, 13.6.1999, Hendrich leg. (Loc. 6/106)” (CLH); 7 exs., “AUSTRALIA/WA/Shire of Wyndham - East Kimberley, Gibb River Road, Drysdale River Crossing, 15°41'2.91"S, 126°22'42.53"E, 380 m, 14.6.1999, Hendrich leg. Loc. 9/109” (CLH); 3 exs., “AUSTRALIA/WA/Shire of Wyndham – East Kimberley, Gibb River Road, King Edward River Crossing, 14°54'10.13"S, 126°12'2.85"E, 280 m, 15.6.1999, Hendrich leg. Loc. 10/110” (CLH); 5 exs., “AUSTRALIA/WA/Shire of Wyndham – East Kimberley, Mitchell Plateau, Surveyors Pool, 14°40'26.54"S, 125°43'56.79"E, 150 m, 17.6.1999, Hendrich leg. Loc. 12/112” (CLH); 19 exs., “AUSTRALIA/WA/Shire of Wyndham – East Kimberley, Gibb River Road, 10 km E Hann River, Snake Creek, 16°30'50.92"S, 126°21'19.53"E, 470 m, 19.6.1999, Hendrich leg. Loc. 16/116” (CLH); 3 exs., “AUSTRALIA/WA/Shire of Wyndham - East Kimberley, Gibb River Raod, Barnett River Gorge, 16°32'15.17"S, 126°7'30.43"E, 450 m, 19.6.1999, Hendrich leg. Loc. 17/117” (CLH); 5 exs., “AUSTRALIA/WA/Shire of Derby – West Kimberley, Gibb River Road, King Leopold Range, 500 m, 5 km NW Mount Bell, intermit. creek, 17°9'43.80"S, 125°18'33.00"E, 23.6.1999, Hendrich leg. Loc. 22/122” (CLH); 6 exs., “AUSTRALIA/WA/Shire of Derby-West Kimberley, Gibb River Road/ Fairfield Leopold Road, Lennard River Bridge, 17°9'48.49"S, 125°13'36.53"E, 50 m, 24.6.1999, Hendrich leg. Loc. 23/123” (ZSM); 6 exs., “AUSTRALIA/ WA/Shire of Halls Creek, Old Halls Creek, Elvire River, 18°14'50.04"S, 127°46'45.39"E, 310 m, 27.6.1999, Hendrich leg. Loc. 28/128” (CLH); 3 exs., “AUSTRALIA/WA, Charnley River, 25 mi E Beverley Springs, 16°15'S, 125°26'E, 9.9.1969, Giuliani, D.D. leg.” (WAM); 1 ex., “Kimberley Research Station, Kununurra nr. Wyndham [-15.46667, 128.1], 27.11.1956” (ANIC); 1 ex., “Western Australia, Pilbara, Millstream [-21.58333, 117.0667], 3.11.1970, Britton, E.B.” (ANIC); 1 ex., “WA Muccangarra Pool 19/10/2008” (DPAW); 7 exs., “WA Mungajee Pool 14/10/2008” (DPAW); 1 ex., “Australia, WA, Pilbara, Hamersley Range, 17 km S Auski Roadhouse, Fig. Tree Crossing 400 m, 28.8.2002, 22°32'S, 118°44'E, Hendrich leg. (Loc. WA 9/173)” (CLH); 1 ex., “Australia, WA, Pilbara, Hamersley Range, Karijini N.P., Dales Gorge [Fortescue Falls and Circular Pool], 400 m, 28.8.2002, 22°29'S, 118°35'E, Hendrich leg. (Loc. WA 10/174)” (CLH); 3 exs., “Australia, WA, Pilbara, Hamersley Range, Karijini N.P., Kalamina Gorge, 450 m, 29.8.2002, 22°25'S, 118°23'E, Hendrich leg. (Loc. WA 11/175)”, two specimens with “DNA Balke 3360”, “DNA Balke 3361” [green printed label] (CLH); 1 ex., “Australia, WA, Pilbara, Hamersley Range, Karijini N.P., Knox Gorge, 450 m, 29.8.2002, 22°21'S, 118°18'E, Hendrich leg. (Loc. WA 12/176)” (CLH); 2 exs., “Australia, WA, Pilbara, De Grey River, River Crossing Hwy. No. 1, 72 km E of Port Hedland, 20 m, 24.8.2002, 20°10'S, 119°11'E, Hendrich leg. (Loc. WA 1/165)” (CLH); 15 exs., “Australia, WA, Pilbara, Yule River, River Crossing Camping Area at Hwy. No. 1, 53 km SW of Port Hedland, 20 m, 24.8.2002, 20°41'S, 118°17'E, Hendrich leg. (Loc. WA 2/166)” (CLH); 1 ex., „Australia, WA, Pilbara, Millstream Chichester N.P., McKenzie Springs, 200 m, 25.8.2002, 21°18'S, 117°12'E, Hendrich leg. (Loc. WA 3/167)” (CLH); 28 exs., “Australia, WA, Pilbara, Millstream Chichester N.P., Gregory Gorge, Palm Pool at River Crossing, 26.8.2002, 21°33'S, 117°03'E, Hendrich leg. (Loc. WA 5/169)” (CLH); 3 exs., “Australia, WA, Pilbara, Millstream Chichester N.P., Fortescue River side branch, SE Visitor Centre, 26.8.2002, 21°37'S, 117°07'E, Hendrich leg. (Loc. WA 6/170)” (CLH); 2 exs., “AUSTRALIA WA, Wyndham, Parry Creek Farm, 11.X.2011, attracted to light at night, J. & S. Nunn (NMPC); 8 exs., “AU Western Australia, E-Pilbara, Weeli Wolli Spr., NW of Newman, 555 m, 22°54.886'S, 119°12.661'E, 19.–22.xi.2011, Sv. Bílý leg.” (NMPC); 1 ex., “AUSTRALIA, WEST AUSTRALIA, HALLS CREEK env., 18°11.48'S, 127°38.42'E, 26.i.2014, J.Horák leg.” (NMPC); 1 ex., “WA, E-Pilbara, Weeli Wolli Springs, NW of Newman, 555 m, 22°54.886'S, 119°12.66'E, 2005, C.H.S. Watts leg.”, DNA Balke 2654”, [green printed label] (ZSM). ***Northern Territory***: 3 exs., “Australia: NT, Anniversary Creek, 12 km S Adelaide River Scenic Route, 43 m, 22.VIII.2006, 13.19.252S, 131.08.271E, L. & E. Hendrich leg. (NT 7)”, three specimens with: “DNA Balke 2173”, “DNA Balke 2174”, “DNA Balke 2175” [green printed labels] (ZSM); 1 ex., “Australia: NT, Nitmiluk NP, Edith Falls, Upper Pool, 123 m, 23.VIII.2006, 14.10.573S, 132.11.537E, L. & E. Hendrich leg. (NT 12)”, “DNA Balke 2165” [green printed label] (ZSM); 1 ex., “Australia: NT, Kakadu Hwy, Bowerbird Creek, 5 km W Mary River Roadh., small puddle, 20 m, 24.VIII.2006, 13.38.142S, 132.10.345E, L. & E. Hendrich leg. (NT 15a)”, “DNA Balke 1660” [green printed label] (ZSM); 1 ex., “Australia, N.T. Waterhole Ranges Owen Springs Reserve, 15.–17.IV.2011 LF; H = 600 m 24°00'33.6"S, 133°24'48.6"E leg. Michael Langer” (ZSM); 25 exs., “Australia, NT: Pungalina Homestead, 16°43'16"S, 137°24'55"E 26 Jun–8Jul 2012 T.A.Weir, N.Gunter, S.Pinzon Navarro at light, open forest by crk” (ANIC); 3 exs., “NT, 80 km W Roper Bar, 14°54'19"S, 133°57'28"E, 78 m, at light, 14.V.2006, leg. Berger & Dostal (7/06)” (CGW); 2 exs., “NT, 40 km v. Port Roper, 14°54'04.8"S, 135°03'24.7"E, 134 m, at light, 14.V.2006, leg. Berger & Dostal” (CGW); 1 ex., “NT, Katherine [-14.46667, 132.2667], 7.2.1968, Watson, J.A.L.” (ANIC); 1 ex., idem, “9.2.1968” (ANIC); 1 ex., “NT Tindal [-14.51667, 132.3667], 1.12.1967, Vestjens, W.J.M.” (ANIC); 1 ex., “Northern Territory Auvergne, Victoria River Bank [-15.63333, 130.4833], 1968, Parker, F.” (ANIC); 1 ex., “Northern Territory, Katherine [-14.46667, 132.2667], 6.2.1968, Matthews, E.” (ANIC); 1 ex., “Northern Territory Magela Ck, nr. rum pipe [Mudginberri Homestead] [-12.7, 132.95], 7.1.1987, Dostine, P.” (ANIC); 1 ex., “Northern Territory, Darwin Coastal, 200 m nth Magela Creek [-11.7, 132.8667], 29.7.1984, Dostine, P.” (ANIC); 2 exs., “Australia: NT, Litchfield NP, TJAYNERA FALLS, 13°15'S, 130°44'E, 63 m, S. Jákl leg., 20–27.XI.2008” (NMPC); 1 ex., “AUSTRALIA: NT, 40 km SW of KATHERINE, rd to Kununurra, 14°44'S, 132°01'E, 100 m, S. Jákl leg., 29.XI.2008” (NMPC); 1 ex., “AUSTRALIA: NT, 25 km S of KATHERINE, nr Cutta Cutta caves, 14°31'S, 132°25'E, 168 m, S. Jákl leg., 23.–31.XII.2008” (NMPC); 1 ex., “Australia: NT, Nitmiluk NP, Edit Falls, 14°10'S, 132°06'E, 37 m, S. Jákl leg., 3.XII.2008” (NMPC); 4 exs., “Australia: NT, 70 km SW of Mataranka, 15°19'S, 132°50'E, 190 m, S. Jákl leg., 22.–23.XII.2008” (NMPC); 5 exs., “AUSTR. NT, Douglas Hot Springs, 12.12.[20]08, 13°45'S, 131°26'E, 35 m, Sv. Bílý leg.” (NMPC); 5 exs., “AUSTRALIA NT, 10 km S of Banka Banka, 18°52'S, 134°04'E, 316 m, 20.12.2008, Sv. Bílý leg.” (NMPC); 1 ex., “AUSTRALIA: NT, BANKA BANKA env., Road to Tennant Creek, 14°53'S, 132°01'E, 316 m, S. Jákl leg., 12–13.I.2009” (NMPC); 1 ex., “AUSTR. NT, 5.1.[20]09, McDonell NP, Serpent Gorge, S.Bílý leg. 715 m, 24°45'S, 132°59'E” (NMPC); 3 exs., “AUSTRALIA NT, Adelaide River township, 25–28 IX 2011, Attracted to light at night. J. Nunn” (NMPC). ***Queensland***: 1 ex., “AUSTRALIA: Qld. 5 km W Mt. Molloy dugout Sept 29, 1990 D. Larson” (ZSM); 2 exs., “AUSTRALIA Qld. Rice field 8 km N Mareeba, Sept 20/90 D. Larson” (ZSM); 1 ex., “AUSTRALIA Qld, 30 km s Atherton stock pond Nov. 27/90 Larson (ZSM); 1 ex., “AUSTRALIA QLD. McLeod R nr base Windsor Tableland Nov. 12/90 Larson” (ZSM); 1 ex., “Mossman 11.viii.1975 R.A. Yule Acc 1150/17 to M.V.”, “QDPC 0-172703” (QDPC); 14 exs., “QL, Greenvale, 150 km NW Charters Towers, 150 m, 19.I.1993, leg. G. Wewalka (12–14)” (CGW); 3 exs., “QL, Marbeeba, 700 m, 22.I.1993, leg. G. Wewalka (19)” (CGW); 10 exs., “QL, Dalrymple, 30 km N Charters Towers, 30 m, 18.I.1993, leg. G. Wewalka (10, 11)” (CGW); 1 ex., “QL, Cape York, VI.1993, leg. Uhler” (CGW); 2 exs., “QL, Richmon, 2.VII.1918, F. M.” (CGW); 1 ex., “QL, 35–47 km SWW Mt. Gamet, 31.I-4.II.2012 leg. S. Prebsl” (CGW); 1 ex., “Einasleigh Uplands Mary Creek [Queensland Tablelands (R), -16.55, 145.2], 4.12.1968, Britton, E.B. & Misko, S.” (ANIC); 1 ex., “QLD, Einasleigh Uplands, 7 miles SW of Mt. Garnet [-17.73332977, 145.0500031], 20.4.1969, Common, I.F.B. & Upton, M.S.” (ANIC); 1 ex., “Cassowary Coast (R) Cardstone [-17.76666667, 145.5833333], 9.3.1966 Hyde, K.” (ANIC); 1 ex., “Queensland Cairns (R), Green Hills [Green Hill] [-17.05, 145.8], 19.12.1967, Brooks, J.G.” (ANIC); 1 ex., “Townsville (C), Crystal Ck., 23 mi. SSE of Ingham [-18.96666908, 146.2666626], 9.12.1968, Misko, S. & Britton, E.” (ANIC); 1 ex., “Burdekin (S), Ayr [-19.56667, 147.4], 4.10.1970, Muir, W.B.” (ANIC); 1 ex., “Cairns” [-16.91667, 145.7667] (ANIC); 1 ex., “Russell River at Bellenden Ker Landing [-17.3, 145.9333], 24.10.1981 Earthwatch, Qld Museum” (ANIC); 1 ex., “Townsville [-19.26667023, 146.8166656] Taylor, F.H.” (ANIC); 1 ex., “Qld 7 miles NNE of Ravenshoe [-17.5, 145.5], 23.4.1969, Common, I.F.B. & Upton, M.S.” (ANIC); 1 ex., “Qld Mary Creek [Queensland Tablelands (R) Einasleigh Uplands -16.55, 145.2], 4.12.1968, Britton, E.B. & Misko, S.” (ANIC); 1 ex., idem, “5.12.1969” (ANIC); 9 exs., “Queensland, [Sep. 1928–July 1929] K.K.Spence”, “K.215166–K.215178” (AMS); 2 exs., “Australia: N QLD, 20 km NE Mareeba, Hodzic Road, 361 m, 12.IX.2006, 16.49.556S, 145.27.211E, L. & E. Hendrich leg. (QLD 28)” (CLH); 2 exs., “Australia: C QLD, 17 km S Calen, Mt. Charlton-Calen Road, creek, 94 m, 23.IX.2006, 21.00.201S, 148.42.546E, L. & E. Hendrich leg. (QLD 44)” (CLH); 6 exs., “Australia: C QLD, 10 km S Mizani, Lake Kinchant, seapage, 48 m, 24.IX.2006, 21.11.580S, 148.53.522E, L. & E. Hendrich leg. (QLD 46)” (ZSM); 2 exs., “Australia: S QLD, 40 km E Bundaberg, Tusky Creek, 9 m, 26.IX.2006, 24.39.139S, 152.01.477E, L. & E. Hendrich leg. (QLD 52)”; 4 exs., “Australia: C QLD, 19 km S Ayr, Bannister Lagoon at Bruce Hwy, swamp, 20 m, 21.IX.2006, 19.33.403S, 147.15.078E, L. & E. Hendrich leg. (QLD 43)” (CLH); 2 exs., “Australia: C QLD, 18 km S Calen, Mt. Charlton-Calen Road, Boulder Creek, 42 m, 23.IX.2006, 21.00.365S, 148.43.231E, L. & E. Hendrich leg. (QLD 45)” (CLH); 14 exs., “Australia, Queensland, Hann River N of Laura, 24. June 1976, J.F. Donaldson” (QDBIP); 1 ex., “Australia, Queensland, Iron Range Cape York Peninsula, 13.–20. May 1975, at light, K.J. Houston” (QDBIP); 1 ex., “Australia, Queensland, McIIwraith Range NE Coen Cape Yorke Pen., 29.VI.–5.VII.1976, J.F. Donaldson” (QDBIP); 1 ex., “Australia, Queensland, 23 km E of Mareeba, 29. January 1989, at light, R.I. Storey” (QDBIP); 1 ex., “Australia, Queensland, Musgrave, 10. Oct. 1982, at light, J.W. Turner” (QDBIP); 1 ex., “Australia, Queensland, Palmer River Crossing Cooktown road, 13.–20. May 1975, at light, K.L. Houston” (QDBIP); 1 ex., “N Queensland, 18.1.2000, Granite Gorge, Sv. Bílý leg.” (NMPC); 17 exs., “N Queensland, 24.1.2000, Gregory Dawns, Sv. Bílý leg.” (NMPC); 4 exs., “N Queensland, 28.1.2000, Porcupine Gorge, Sv. Bílý leg.” (NMPC); 14 exs., “N Queensland, 29.1.–3.2.2000, The Lynd Junction, Sv. Bílý leg.” (NMPC); 1 ex., “N Queensland, 5.2.2000, Ravenshoe, Sv. Bílý leg.” (NMPC); 2 exs., “N Queensland, 7.2.2000, Mt. Carbine, Sv. Bílý leg.” (NMPC); 17 exs., “N Queensland, 13.2.2000, Undara, Sv. Bílý leg.” (NMPC); 5 exs., “Australia Qld, Mt Isa, Moondarra rd, 13 IX 2011, In gravel at edge of Leichaerdt R., J. Nunn” (NMPC). ***New South Wales***: 1 ex., “NSW, Wakool”, “K.363073” (AMS). ***South Australia***: 1 ex., “SA Elliston Salt Lake 23/6 1974 Timm BV”, “25-002059” (SAMA).

##### Description of male.

***Body shape:*** In dorsal view, elongate oval, broadest at midlength of elytra, moderately convex. Body outline without discontinuity between pronotum and elytra. Head relatively broad, anterior margin of clypeus truncate. Pronotum broadest between posterior angles, lateral margins moderately curved. Base of elytra as broad as pronotal base; lateral margins of elytra moderately curved (Fig. 8).

***Colouration:*** Body yellow to pale ferruginous, disc of pronotum and ventral side diffusely darker, elytral striae sharply outlined in black, appendages testaceous. Base of elytra with narrow yellowish marking (Fig. 8).

***Dorsal surface sculpture:*** Whole surface shiny (Fig. 8). Head uniformly microreticulated, reticulation composed of moderately deeply impressed isodiametric meshes. Punctation composed of small punctures, smaller than meshes of reticulation; rows of coarse punctures present around inner margin of eyes and in small depression anterolaterally of eyes. Pronotum with lateral beading very thin and indistinct. Microreticulation and punctation similar to that of head; row of coarse setigerous punctures present along anterior margin, basal margin (except for basomedially), and laterally close to sides. Elytra with microreticulation similar to that of head and pronotum, but less impressed. On each elytron 11 sharply incised discal striae present, inner three striae only weakly impressed anteriorly.

***Antennae and legs:*** Antenna with antennomeres long and slender. Protibia modified, slightly broadened anteriorly. Protarsomeres 1–3 distinctly broadened and mesotarsomeres less so, with adhesive discs on their ventral side; claws simple.

***Ventral part:*** Finely microreticulated, with intermixed, sparsely distributed, very small punctures. Meshes isodiametric, except for metacoxae and abdominal ventrites 1–2 (longitudinal), abdominal ventrite 3 (diagonal anteriorly, transverse posteriorly), and abdominal ventrites 4–6 (transverse). Prosternum obtusely keeled medially. Prosternal process shortly lanceolate, apex obtuse; distinctly bordered laterally. Lateral parts of metaventrite tongue-shaped, slender. Metacoxal lines nearly complete, absent only close to metaventrite. Metacoxae and abdominal ventrites 1–3 with numerous longitudinal or oblique striae.

***Male genitalia:*** Median lobe consisting of a few sclerites, well separated apically (Fig. 17A, B, C). Shape of paramere narrowly triangular, with very dense, strong, long setae along dorsal margin (Fig. 17D).

##### Female.

Similar to male in habitus. Protibia simple, not angled basally and only slightly broadened distally; pro- and mesotarsomeres not broadened, without adhesive setae.

##### Measurements.

TL = 5.5–5.8 mm; TL-H = 4.9–5.2 mm; MW = 2.5–2.6 mm.

##### Variability.

All specimens studied are rather uniform and vary only in body length.

##### Differential diagnosis.

*Copelatus nigrolineatus* is distinguished from all other Australian species by the absence of submarginal striae on the elytra, the pale dorsal surface and the form of the median lobe.

##### Distribution.

Endemic. The species is widely distributed in the northern half of Australia. Numerous records are from the Northern Territory, north-western Australia (Kimberley region and the Pilbara), and Queensland south to Brisbane (Fig. 26). The single records from South Australia (“Elliston”, SAMA) and New South Wales (“Wakool”, AMS) are in need of confirmation. Most probably those single specimens were drifted southwards by heavy thunderstorms, followed by flash floods.

##### Habitat.

This widely distributed species is the most common *Copelatus* in the Northern Territory and north-western Australia and was found in almost all habitat types. It occurs in both open country as well as forested areas. Most specimens were obtained from isolated flood-zone pools of seasonal rivers and pools adjacent to streams in Eucalypt or tropical woodland. The species tended to be found more in seepage areas or on mineral substrates than other the Australian *Copelatus* (Larson 1993). Also, a few specimens were taken from slow flowing spring-fed streams (Figs 34B, 35A, B). *Copelatus nigrolineatus* is also attracted to light.

This species is the most common Australian species of the genus in Australian entomological collections.

#### Copelatus portior

Taxon classificationAnimaliaColeopteraDytiscidae

Guignot, 1956

39BB5ACC-1DDF-5562-8B61-8DB88D84C55A

**Figure 9.**
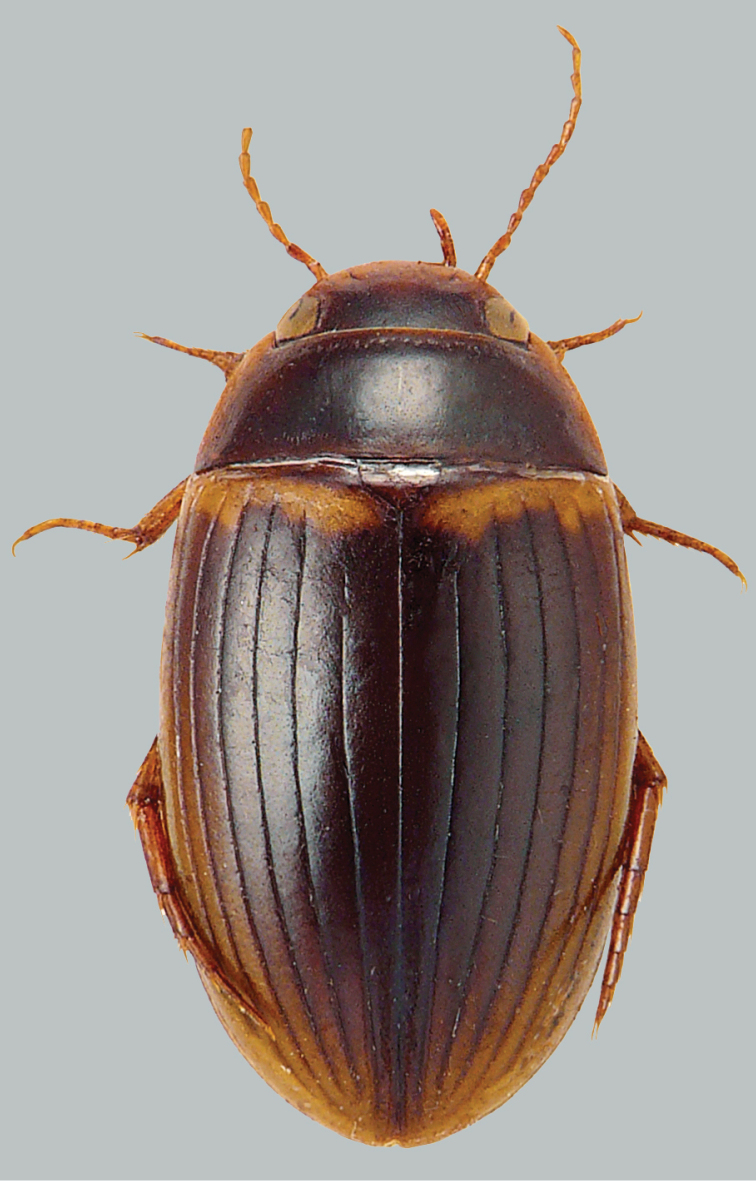
Habitus and colouration of *Copelatus portior*, female. Total length 5.4 mm.

Figures 9 , 18 , 24 , 33

Copelatus gentilis ab. divisus J. Balfour-Browne 1939: 79 (unavailable name).
Copelatus portior Guignot 1956: 53 (original description); Nilsson and Hájek 2019: 59 (catalogue).
Copelatus divisus Watts, 1978: 122 (original description); Watts 1985: 26 (general distribution); Larson 1993: 52 (habitat information); Watts 2002: 42 (checklist); Nilsson and Hájek 2019: 57 (catalogue); syn. nov.

##### Type localities.

*Copelatus divisus*: “New Hebrides [Vanuatu], Malekula Island”. *Copelatus portior*: “Seleo, Berlinhafen” [Papua New Guinea, Sandaun Province, Seleo Island, 03°08'39.2"S, 142°28'56.9"E].

##### Type material of *Copelatus divisus*.

***Holotype:*** Male, “Holotype” [printed label with red frame]; “New Hebrides: NE Malekula. i.1930. L.E. Cheesmann. B.M. 1930-178.” [printed label]; “1768” [hw label]; “Copelatusgentilis var. divisus, var. nov. J. Balfour-Browne det” [hw label] (NHMUK). ***Paratype***: Male, “Paratype” [printed label with yellow frame]; “New Hebrides: NE Malekula. i.1930. L.E. Cheesmann. B.M. 1930-178.” [printed label]; “1768” [hw label]; “Copelatusgentilis var. divisus, var. nov. J. Balfour-Browne det” [hw label] (NHMUK).

##### Type material of *Copelatus portior*.

***Holotype:*** Male, “N. Guinea Biró 96 [printed label]”, “Seleo Berlinhaf. [printed label]”, “♂” [printed label], “Type” [printed, red label with black frame], “Holotypus [printed, red typing] 1955 ♂ [hw] Copelatus portior Guignot” [hw, white label with red frame], “F. Guignot det., 19 [printed] 55 [hw] Copelatus portior n.sp. Holotype ♂ [Guignot’s hw]” (HNHM).

##### Additional material studied (64 specimens).

***Northern Territory:*** 2 exs., “AUSTRALIA NT Darwin, Holmes Jungle Pk uv lght Dec. 2/93 S Peck” (ZSM); 1 ex., “Australia, Northern Territory, Berry Springs [-12.72193, 131.01027] 30/10/1991–04/11/1991, Wells, A. & Webber, Malaise trap, Field Collected – Terrestrial” (AMS). ***Queensland***: 5 exs., “Australia: N QLD, Cape Tribulation Road S of ferry station, forest swamp, 12 m, 15.IX.2006, 16.17.469S, 145.19.122E, L. & E. Hendrich leg. (QLD 35)”, “DNA Balke 1874” [green printed label] (CLH, ZSM); 1 ex., “Australia: C QLD, 18 km S Calen, Mt. Charlton-Calen Road, Boulder Creek, 42 m, 23.IX.2006, 21.00.365S, 148.43.231E, L. & E. Hendrich leg. (QLD 45)”, “DNA Balke 2762” [green printed label] (ZSM); 6 exs., “Australia, QL, 10 km S Tully, S Innisfail, 30 m, 25.I.1993, leg. G. Wewalka (26)” (CGW); 1 ex., “Australia, QL, 15–20 km S Innisfail, 20 m, 24.I.1993, leg. G. Wewalka (24)” (CGW); 1 ex., “Australia, Queensland, Cape York, Lockerbie [-10.8, 142.46679], 3.4.1964, Common, I.F.B. & Upton, M.S. leg.” (ANIC); 1 ex., “Australia, Queensland, Cairns [-16.91667, 145.7667], Taylor, F.H. leg.” (ANIC); 1 ex., “Australia, Queensland, Cassowary Coast (R) 2 miles W of Mission Beach [-17.86667, 146], 18.4.1969, Common, I.F.B. & Upton, M.S. leg.” (ANIC); 1 ex., “Australia, Queensland, Cape York Peninsula, 2 miles S Iron Range [-12.73332977, 143.2832947], 12.5.1971, Brooks, J.G. leg.” (ANIC); 1 ex., “Australia, Cape York Peninsula, Queensland Gordon´s Ck., Iron Ra. [Gordon Creek, Iron Range, -12.71667004, 143.3166962], 10.5.1971, J.G. Brooks” (ANIC); 1 ex., “Gordon's CR [Creek] Iron RA [Range] N.Q. [North Queensland] 100' [100 m] 12.5.71 J.G. Brooks at light” (CLH); 1 ex., “Queensland, 8 km West of Kuranda [-16.807954, 145.583013], 3 Apr 1982, G. O’Reilly”, “K.363070” (AMS); 1 ex., “Australia, Queensland [-16.81985, 145.63693], 26 Apr 1987, F. Sattler”, “K.363071” (AMS); 2 exs., “Australia QLD01/32 Saltbag Ck., 18 km w. Mt. Molloy, 13.4.2001, M. Baehr” (CLH); 1 ex., “Australia NE QLD Conway Range N.P. E from Proserpine”, “at light 17.–23.II.1981 leg. Hangaya, Herozeg & Vojnits” (CLH); 1 ex., “Australia, Queensland, Iron Range Cape York Pen., 26. May 1971-2. June 1971, B.K. Cantrell” (QM); 1 ex., “Australia, Queensland, West Claudie River 4 km SW road junction, 25. June 1982, mv lamp, G. Daniels, M.A. Schneider” (QM); 2 exs., “Australia, Queensland, Walkamin, 26. March 1984, light trap, J.D. Brown” (QDPIB); 1 ex., “Australia, Queensland, Cairns, February 1946” (QDPIB); 1 ex., “Australia Queensland Arriga R.S. via Mareeba, 2.–3. December 1976, at light R.I. Storey” (QDPIB); 1 ex., “Australia, Queensland, Davies Creek via Mareeba, 13. March 1981, R.I. Storey” (QDPIB); 2 exs., “Australia Queensland Cow Bay N of Daintree, 7.–20. February 1984, I. C. Cunningham” (QDPIB); 3 exs., “Australia, Queensland, South Johnstone R.S., 29. Nov. 1991–14. Jan. 1992, Malaise Trap, K.H. Halfpapp” (QDPIB); 1 ex., “Australia, Queensland, Mossman River, 25. November 1984, light trap, J.D. Brown” (QDPIB); 2 exs., “Australia, Queensland, Atherton Area, 14. March 1983, light trap, J.D. Brown” (QDPIB); 1 ex., “Australia Queensland 15 km WNW of South Johnstone 4. March 1986, light trap, Fay, Halfpapp” (QDPIB); 1 ex., “Australia, Queensland, 15 km WNW of South Johnstone, 19. Dec. 1985, light trap, Fay, Halfpapp” (QDPIB); 1 ex., “Australia, Queensland, 15 km WNW of South Johnstone, 26. May 1986, light trap, Fay, Halfpapp” (QDPIB); 1 ex., “Australia, Queensland, Tolga, 23. January 1986, light trap, J.D. Brown” (QDPIB), 1 ex., “QLD, Mackay [-21.1500, 149.1833] 4/4 1963, CHS Watts”, “25-001796” (SAMA); 1 ex., “QLD, Burdekin, Home Hill [-19.6669, 147.4167] 4/7 1963, CHS Watts”, “25-001798” (SAMA); 1 ex., “QLD, Townsville [-19.2, 146.68], 6–11/2/ 1998, AJ Watts”, “25-001803” (SAMA); 4 exs., “AUSTRALIA Qld Ellis Beach 30 km N Cairns J. 11 1991 Dr. Larson” (ZSM); 6 exs., “N Queensland, 14.1.2000, Kuranda, Sv. Bílý leg.” (NMPC); 2 exs., “N Queensland, 9.2.2000, Kuranda, Sv. Bílý leg.” (NMPC); 1 ex., “N Queensland, 15.1.2000, Davis Creek, Sv. Bílý leg.” (NMPC).

##### Description of male.

***Body shape:*** In dorsal view broad, ovoid, broadest part in midlength of elytra. Body outline without discontinuity between pronotum and elytra. Head relatively broad; anterior margin of clypeus not bordered. Pronotum broadest at base, lateral margins moderately curved. Base of elytra as broad as pronotal base; lateral margins of elytra moderately curved (Fig. 9).

***Colouration:*** Body dark brown to black, anterior angles of pronotum, base of elytra, appendages and parts of abdomen testaceous.

***Dorsal surface sculpture:*** Whole surface almost matt (Fig. 9). Head uniformly microreticulated, reticulation strongly impressed with small meshes. Densely, and moderately strongly punctate; rows of coarse punctures present around inner margin of eyes and in small depression anterolaterally of eyes. Pronotum with lateral beading very thin and indistinct. Microreticulation and punctation similar to that of head; row of coarse punctures present along anterior margin, basal margin (except for basomedially), and laterally close to sides. Sides of pronotum with several short, sharp strioles. Elytra with microreticulation similar to that of head and pronotum but lacking serial punctures except for lateral row. Each elytron with six well marked striae and one submarginal stria becoming progressively closer towards sides, striae 1 and 4 continue farthest towards apex and stria 6 the least distance.

***Antennae and legs:*** Antenna with antennomeres long and slender. Protibia slightly modified, slightly broadened anteriorly. Protarsomeres distinctly and mesotarsomeres 1–3 less broad, with adhesive discs on their ventral side; claws simple.

***Ventral part:*** Finely microreticulated, with sparsely distributed, very small punctures. Prosternum obtusely keeled medially. Prosternal process quite strong, convex, narrowly flanged and bluntly pointed. Lateral parts of metaventrite tongue-shaped, very slender. Metacoxal lines very close posteriorly, strongly diverging anteriorly. Metacoxae with well-marked striae and abdominal ventrites 1–4 with larger but weaker longitudinal striae.

***Male genitalia:*** Median lobe anchor-like, narrow, medially bifid: left lobe short, hammer-like, right lobe long, thin, pointed and strongly curved downwards apically in lateral view (Fig. 18A, B, C). Paramere broadly triangular, with weak, relatively short setae mainly along dorsal margin of subdistal part (Fig. 18D).

##### Female.

Similar to male in habitus. Protibia simple, not angled basally and only slightly broadened distally; pro- and mesotarsomeres not broadened, without adhesive setae.

##### Measurements.

TL = 5.0–5.7 mm; TL-H = 4.7–5.3 mm; MW = 2.7–2.85 mm.

##### Variability.

All specimens studied are rather uniform and vary only in body length and dorsal colouration. In some specimens, sides of elytron and areas between striae often lighter. A single female from Holmes Jungle Park in Darwin is on dorsal surface almost black, matt and with coarse microreticulation and numerous strioles on elytra and pronotum.

##### Differential diagnosis.

*Copelatus portior* can be separated from the similar *C. tenebrosus* and *C. martinbaehri* sp. nov. by its broad and ovoid body shape, colouration of pronotum and elytra, and the six well-developed and strong elytral striae. Furthermore, it is the only Australian species with an anchor-like median lobe.

##### Comments on classification.

*Copelatus portior* is closely related to the widespread Oriental species *Copelatus oblitus* Sharp, 1882 with which it shares a characteristic shape of male genitalia with anchor-like, medially bifid median lobe. We compared the holotypes of both *C. portior* and *C. divisus* and have found no differences between these two taxa. Therefore, we consider *Copelatus divisus* Watts, 1978 a junior subjective synonym of *Copelatus portior* Guignot, 1956.

##### Distribution.

The species occurs in the coastal tropical rainforest areas of the Northern Territory and north-eastern Queensland, from northern Peninsula (Lockerbie) to Mackay in the south (Fig. 24). Additionally, *C. portior* is known from northern part of Papua New Guinea: Sandaun Province, Seleo Island, and Madang Province, Friedrich-Wilhelmshafen, now Madang, (Guignot 1956: 53, localities for holo- and allotypes).

##### Habitat.

*Copelatus portior* was mainly collected in puddles and pools in temporary flooded swamp forests (Fig. 33A, B), low gradient streams and drainage ditches at the edge of cane fields (Larson 1993), and in residual pools of shallow, intermittent and smaller creeks. The beetles hide among fallen leave litter and dense emergent grasses. The substrate was generally clay. Larson (1993) collected most of his specimens in open sites where rainforest had been cleared. *Copelatus portior* is commonly attracted at light. The species is regularly collected but never abundant or in larger numbers.

#### Copelatus tenebrosus

Taxon classificationAnimaliaColeopteraDytiscidae

Régimbart, 1880

70D526A4-5F0D-55BD-877C-2D73640C5101

**Figure 10.**
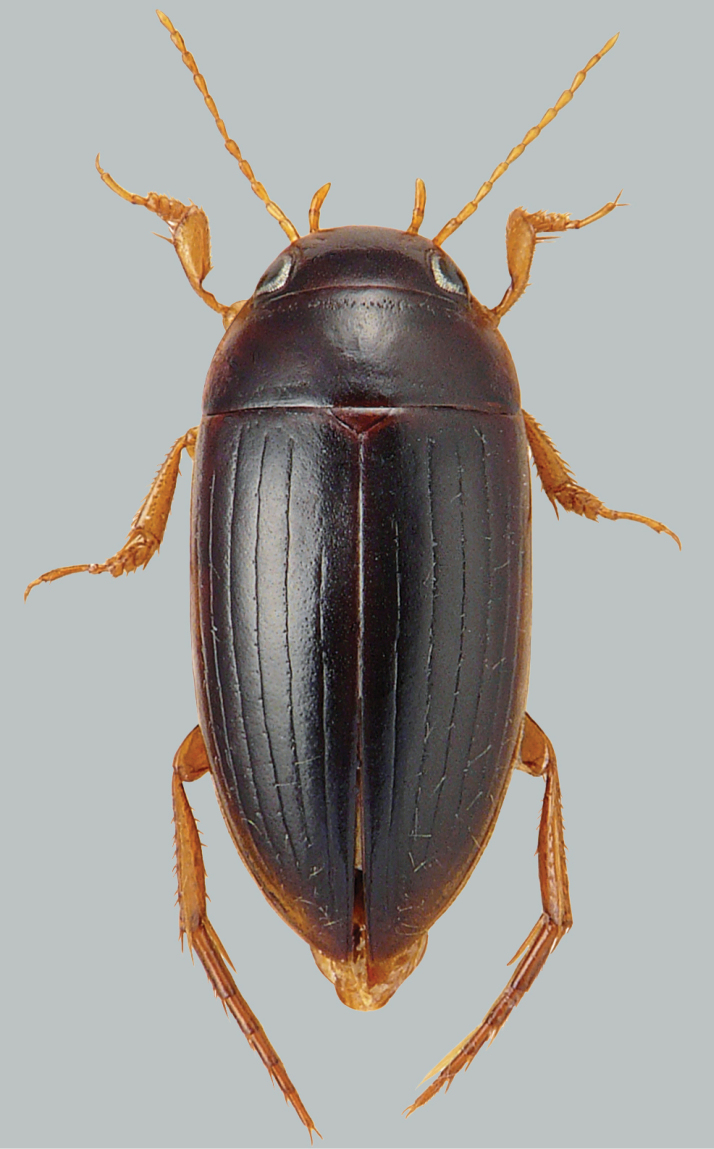
Habitus and colouration of *Copelatus tenebrosus*, male. Total length 4.4 mm.

Figures 10 , 19 , 27 , 33 , 36

Copelatus tenebrosus Régimbart 1880: 210 (original description); Watts 1978: 124; Watts 1985: 26; Larson 1993: 53; Hendrich and Balke 1995: 44; Watts 2002: 33, 42; Hendrich et al. 2004: 118. 1

##### Type locality.

“[Indonesia, Sumatra] Solok, District of Rawas, Soeroelangoen”.

##### Type material.

Not studied. The type specimens should be deposited in the Naturalis Biodiversity Center, Leiden, The Netherlands (former Rijksmuseum van Natuurlijke Historie), but they were not found during a visit of JH. The designation of the lectotype by Watts (1978: 124) based on specimens from “Siam, Bangkok” and deposited in NHMUK is invalid and concerns almost surely the type material of *Copelatus pusillus* Sharp, 1882 (a junior subjective synonym of *C. tenebrosus*); *Copelatus tenebrosus* was described based on specimens from Sumatra.

##### Additional material studied (83 specimens).

***Northern Territory:*** 2 exs., “AUSTRALIA NT Kakadu NP Alligator R. Gungaree Rainforest Dec. 22/93 S&J Peck” (ZSM); 2 exs., “Australia N.T. Howard Springs, 35 m, 17–18.5.2014 ca. 25 km SE Darwin ca. 12°29.82.7"S, 131°01.95.1"E M. Langer leg. (LF) Coll. Hendrich” (ZSM); 1 ex., “Australia NT Burdulba Billabong, 15 km SSW Jabiru, 12 m, 12.46.26S 132.44.86E, 2.–3.XI.2007, at light, M. Baehr leg.” (ZSM); 1 ex., “Australia: NT, Georgetown Billabong, 750 m E Jabiru East, 30 m, 29.VIII.2006, 12.40.716S 132.55.861E, L. & E. Hendrich leg. (NT 20)”, “DNA Balke 2411” [green printed label] (ZSM); 1 ex., “NT, Tindal near Katherine [-14.51667, 132.3667], 1.12.1967, Vestjens, W.J.M.” (ANIC); 1 ex., “NT, Darwin, 12°51'24"S, 131°46'48"E, 52 m, at light, 9.V.2006, leg. Berger & Dostal (2/06)” (CGW); 1 ex., “NT, Kakadu NP, Muirella, 40 m, at light, 10.V.2006, leg. Berger & Dostal (3/06)” (CGW); 1 ex., “NT, Litchfield NP, Florence Falls, 12°51'15"S, 132°45'16"E, 63 m, at light, 12.V.2006, leg. Berger & Dostal (5/06)” (CGW); 1 ex., “Australia, Northern Territory, Muirella Park Kakadu, 18. May 1985, at light, Fay, Halfpapp” (QDPIB); 22 exs., “Australia: NT, Litchfield NP, TJAYNERA FALLS, 13°15'S, 130°44'E, 63 m, S. Jákl leg., 20–27.XI.2008” (NMPC); 3 exs., “Australia: NT, Nitmiluk NP, Edit Falls, 14°10'S, 132°06'E, 37 m, S. Jákl leg., 3.XII.2008” (NMPC); 1 ex., “AUSTR. NT, Kakadu NP, Jim Jim Billabong, 12°56'S, 132°33'E, 5 m, 5.–8.12.[20]08, Sv. Bílý leg.” (NMPC); 2 exs., “AUSTRALIA NT, Kakadu NP, Ubirr, 12°25'S, 132°57'E, 190 m, 9.–10.12.2008, Sv. Bílý leg.” (NMPC); 2 exs., “AUSTR. NT, Douglas Hot Springs, 12.12.[20]08, 13°45'S, 131°26'E, 35 m, Sv. Bílý leg.” (NMPC); 1 ex., “AUSTR. NT, Litchfield NP, 40 km E of Daly, 14.12.[20]08, Sv. Bílý leg.” (NMPC); 3 exs., “AUSTRALIA: NT, 70 km SW of MATARANKA, 15°19'S, 132°50'E, 190 m, S. Jákl leg., 22.–23.XII.2008” (NMPC); 1 ex., “AUSTRALIA: NT, 25 km S of KATHERINE, nr Cutta Cutta caves, 14°31'S, 132°25'E, 168 m, S. Jákl leg., 23.–31.XII.2008” (NMPC). ***Queensland***: 8 exs., “Australia: N QLD, Cape Tribulation Road S of ferry station, forest swamp, 12 m, 15.IX.2006, 16.17.469S, 145.19.122E, L. & E. Hendrich leg. (QLD 35)”, two specimens with “DNA Balke 1875”, “DNA Balke 1876” [green printed label] (CLH, ZSM); 1 ex., “Australia: C QLD, 10 km S Mizani, Lake Kinchant, seapage, 48 m, 24.IX.2006, 21.11.580S, 148.53.522E, L. & E. Hendrich leg. (QLD 46)”, “DNA Balke 1756” [green printed label] (ZSM); 1 ex., “QLD Ingham [-18.65, 146.1666667], 23.2.1960, Harley, K.L.” (ANIC); 1 ex., “QLD Ingham [-18.65, 146.1666667], 27.1.1968, Brooks, J.G.” (ANIC); 1 ex., “QLD, 2 miles W of Mission Beach [-17.86667, 146.1], 18.4.1969, Common, I.F.B. & Upton, M.S.” (ANIC); 1 ex., “Australia Queensland 65 km East of Hughenden [-20.8287, 144.7969], 3 Feb 1981, M.S. Moulds” (AMS); 11 exs., “QL, 10 km S Tully, S Innisfail, 30 m, 25.I.1993, leg. G. Wewalka” (CGW); 2 exs., “Australia, Queensland, The Boulders Babinda, 5. February 1975, at light in rainforest B.K. Cantrell” (QDPIB); 1 ex., “Australia, Queensland, Saibai Island, 09.23S 142.40E, 6. February 1986, at light, Houston, Hamacek” (QDPIB); 1 ex., “Australia, Queensland, Wallaman Falls Area W Ingham, 7. February 1975, at light in rainforest, B.K. Cantrell” (QDPIB); 6 exs., “Australia, Queensland, 15 km WNW of South Johnstone April-May 1988, light trap, Fay, Halfpapp” (QDPIB); 1 ex., “Australia, Queensland (22°29'13"S, 144°25'54"E) [Barcadine] Taylor, Frank H. (Collector)” (AMS); 2 exs., “Australia Qld 15 km N Cairns Jan 11, 1991 D.J. Larson” (ZSM).

##### Description of male.

***Body shape:*** In dorsal view, elongate oval, broadest in basal third of elytra, moderately convex. Body outline with little discontinuity between pronotum and elytra. Head relatively broad; anterior margin of clypeus truncate. Pronotum broadest just before posterior angles, lateral margins moderately curved. Base of elytra slightly broader than pronotal base; lateral margins of elytra moderately curved (Fig. 10).

***Colouration*.** Body dark red-brown to black, clypeus anteriorly, anterior angles of pronotum, sides of elytron towards apex, parts of ventral side and appendages yellowish to dark ferruginous (Fig. 10).

***Dorsal surface sculpture:*** Whole surface shiny (Fig. 10). Head uniformly microreticulated, reticulation composed of moderately deeply impressed meshes. Punctation composed of coarse setigerous punctures, and very small punctures spread sparsely on surface; rows of coarse punctures present around inner margin of eyes and in small depression anterolaterally of eyes. Pronotum with lateral beading very thin and indistinct. Microreticulation and punctation similar to that of head; row of coarse setigerous punctures present along anterior margin, basal margin (except basomedially), and laterally close to sides. Posterior angles of pronotum with quite dense shallow short striae. Elytra with microreticulation similar to that of head and pronotum, but less impressed. On each elytron six well impressed discal and one submarginal longitudinal striae, progressively closer towards sides; stria 1 (sutural stria) shortened at basal third, striae 2–4 almost complete, striae 5 and 6 a little shorter basally. Submarginal stria expanding from behind midlength of elytron to end of stria 5. Serial punctures on elytra, in striae 2–4 and 6, rather weak.

***Antennae and legs:*** Antenna with antennomeres long and slender. Protibia strongly modified, distinctly broadened anteriorly (2/3^rd^) and strongly narrowed basally (1/3^rd^). Pro- and mesotarsomeres 1–3 moderately broadened, with adhesive discs on their ventral side; claws simple.

***Ventral part:*** Strongly microreticulated, with intermixed, sparsely distributed, very small punctures. Prosternal process narrow, distinctly bordered laterally, bluntly pointed at tip. Lateral parts of metaventrite (“metasternal wings”) tongue-shaped, slender. Metacoxal lines very close posteriorly, moderately diverging anteriorly. Metacoxae and abdominal ventrites 1–3 with several longitudinal striae of different lengths.

***Male genitalia:*** Median lobe apically more or less evenly narrow in ventral view, and very strongly curved downwards in lateral view (Fig. 19A, B, C). Shape of paramere broad triangular, with weak, short setae along dorsal margin of subdistal part (Fig. 19D).

##### Female.

Similar to male in habitus. Protibia simple, not angled basally and only slightly broadened distally; pro- and mesotarsomeres not broadened, without adhesive setae.

##### Measurements.

TL = 4.2–4.5 mm; TL-H = 3.8–4.1 mm; MW = 1.9–2.0 mm.

##### Variability.

All Australian specimens studied are rather uniform and vary only in body length.

##### Differential diagnosis.

The species is similar to *C. marginatus* but can be easily separated by the smaller size (smallest species of the genus in Australia), fully developed inner striae of elytra, and the shape of the median lobe.

##### Distribution.

This is the most widespread species in the Indomalayan and Australasian realms. It occurs from Nepal, India, and Sri Lanka over Myanmar, Laos, Vietnam, Thailand, Philippines, Indonesia (Hendrich and Balke 1995), Malaysia, and New Guinea (Hendrich et al. 2004) to coastal northern and eastern Australia, south to Townsville (Fig. 27).

##### Habitat.

The wide distribution of this species owes to the ability of adaptation to manmade habitats like rice or paddy fields and shallow irrigation ditches (Hendrich et al. 2004). In Australia, *C. tenebrosus* inhabits open, treeless and seasonally flooded meadows, billabongs, ponds, paddy fields (Larson 1993), puddles, swamps and roadside ditches with dense vegetation, often with mats of floating grasses (Figs 33A, B, 36A, B). The species is not that common in Australia as it is in many other countries of Southeast Asia (Hendrich and Balke 1995; Hendrich et al. 2004), and its population density in one spot is always very low.

### Checklist of the Australian species of *Copelatus*, in alphabetic order

*Copelatus bakewelli* Balfour-Browne, 1939 Australia: northern parts of WA, NT, QLD

*Copelatus clarki* Sharp, 1882 Australia: WA, NT, QLD; Indonesia: Papua Province; Papua New Guinea: Western Province

*Copelatus daemeli* Sharp, 1882 Australia: northern parts of WA, NT, QLD

*Copelatus irregularis* W.J. Macleay, 1871 Australia: northern parts of WA, NT, QLD

*Copelatus marginatus* Sharp, 1882 Australia: northern parts of WA, NT, QLD; New Caledonia, West Samoa, Samoa, Tonga, Fiji (?), New Guinea (?)

*Copelatus martinbaehri* sp. nov. Australia: QLD (Cape York Peninsula); Papua New Guinea (Central Province)

*Copelatus nigrolineatus* Sharp, 1882 Australia: WA, NT, QLD, SA, NSW (?)

*Copelatus portior* Guignot, 1956 Australia: NT, QLD; Papua New Guinea: Sandaun and Madang provinces

*Copelatus tenebrosus* Régimbart, 1880 Australia: northern parts of NT and QLD; Asia, Southeast Asia and New Guinea

### Key to Australian *Copelatus* (modified after Watts 1978)

**Table:** 

**1**	Elytron with a submarginal stria and six or more additional discal striae	**2**
–	Elytron without submarginal stria, but with 11 discal striae. Body colour yellow to pale ferruginous, elytral striae sharply outlined in black (Fig. 8). Median lobe as in Fig. 17	***C. nigrolineatus***
**2**	Elytron with 11 striae	**3**
–	Elytron with 6–10	**5**
**3**	Smaller species, TL = 5.1–5.25 mm. Elytron uniformly black without any testaceous basal or apical markings on elytra (Fig. 1), apart from paler humeral angle. Pronotal striae weak. Median lobe as in Fig. 11	***C. bakewelli***
–	Larger species, TL = 6.2–7.8 mm.	**4**
**4**	Elytron with testaceous basal and apical markings (Fig. 5). Pronotal striae strong and short. Median lobe as in Fig. 14	***C. irregularis***
–	Elytron dark brown to black (Fig. 3). Pronotal striae absent. Median lobe as in Fig. 13.	***C. daemeli*** 2
**5**	Elytron with 6 striae	**6**
–	Elytron with more than 6 striae	**9**
**6**	Inner stria of elytron short, less than half length of elytron. TL = 5.7–6.0 mm. Median lobe as in Fig. 15	***C. marginatus***
–	Inner stria of elytron long, at least 2/3^rd^ the length of elytron or longer	**7**
**7**	Elytron dark brown to black, without any testaceous basal marking (Fig. 10). Smallest species, TL = 4.2–4.5 mm. Outer stria of elytron short, not reaching shoulder of elytron. Median lobe as in Fig. 19	***C. tenebrosus***
–	Elytron with at least narrow testaceous basal marking (Fig. 9). Larger species, TL = 5.0–6.75 mm	**8**
**8**	Body broad, ovoid. TL = 5.0–5.7 mm. Outer stria of elytron complete. Anterior angles of pronotum and base of elytra narrowly testaceous (Fig. 9). Median lobe as in Fig. 18	***C. portior***
–	Body oblong-oval. TL = 6.2–6.75 mm. Outer stria on elytron almost complete. Anterior angles of pronotum, base and tip of elytra testaceous (Fig. 7). Median lobe as in Fig. 16	***C. martinbaehri* sp. nov.**
**9**	Elytron with 8 striae, no striae inwards from 1^st^ row of serial punctures (Fig. 2). Largest species, TL = 7.2–8.0 mm. Median lobe as in Fig. 12	***C. clarki***
–	Elytron with 10 striae, inner striae partially reduced (Fig. 4). Smaller species, TL = 6.2–6.3 mm. Median lobe as in Fig. 13	***C. daemeli***

**Figure 11.**
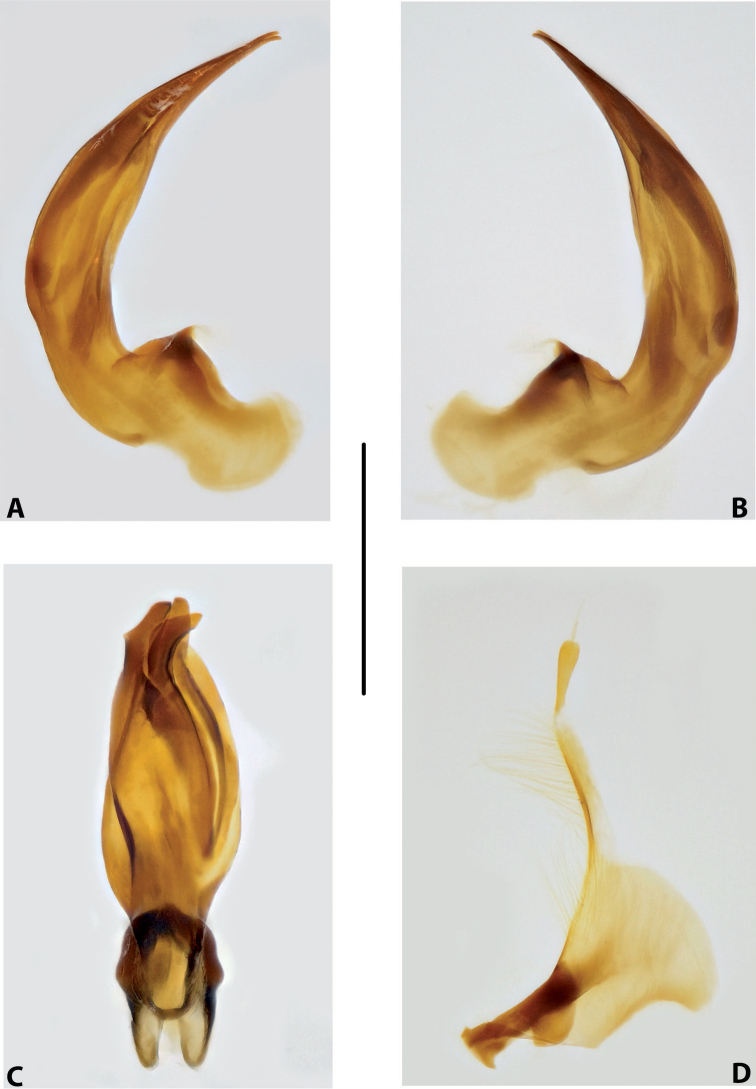
*Copelatus bakewelli***A** median lobe in lateral view, right side **B** median lobe in lateral view, left side **C** median lobe in ventral view **D** left paramere in external view. Scale bar: 0.5 mm.

**Figure 12.**
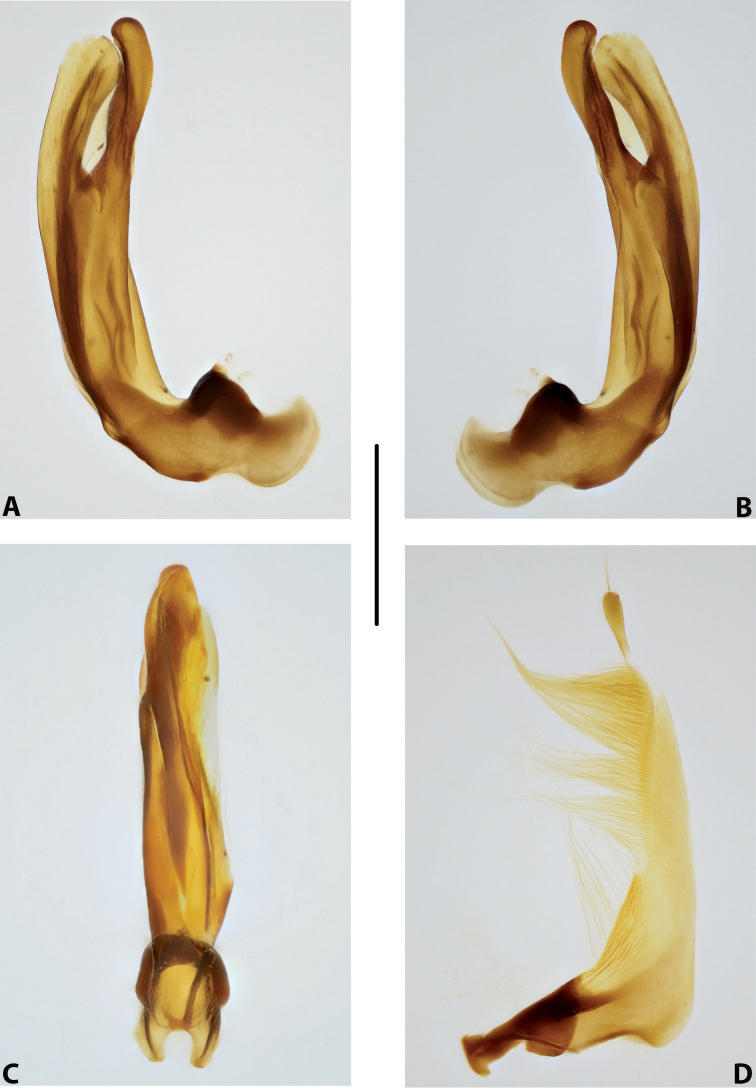
*Copelatus clarki***A** median lobe in lateral view, right side **B** median lobe in lateral view, left side **C** median lobe in ventral view **D** left paramere in external view. Scale bar: 0.5 mm.

**Figure 13.**
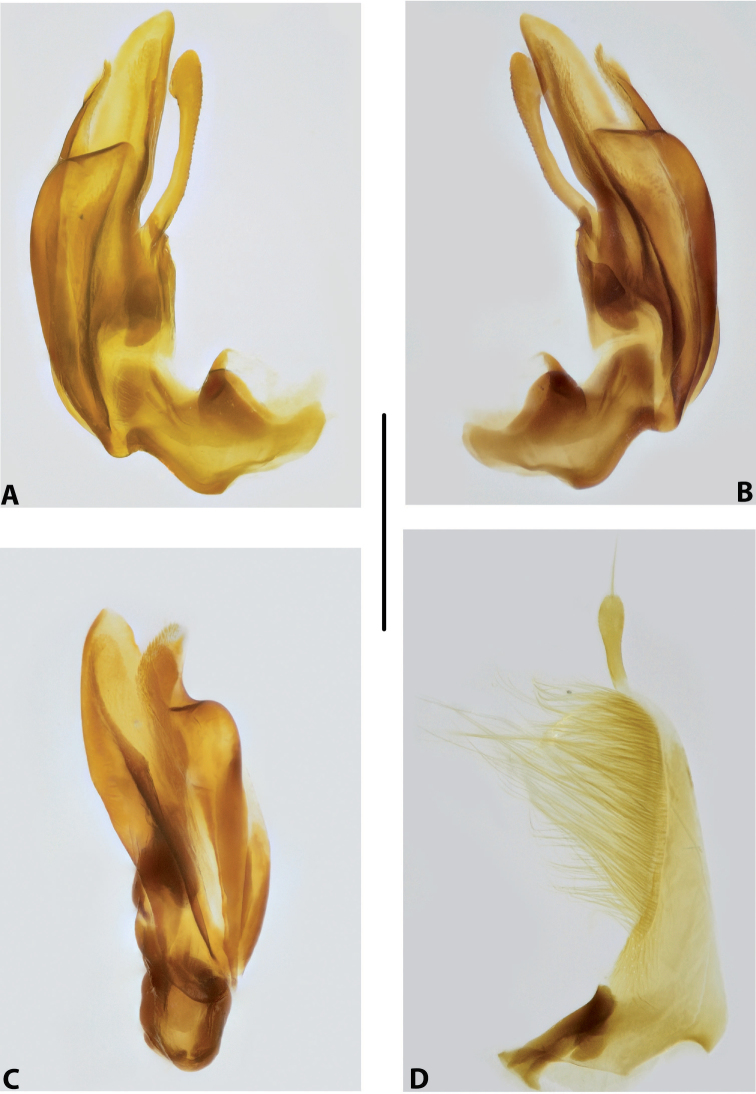
*Copelatus daemeli***A** median lobe in lateral view, right side **B** median lobe in lateral view, left side **C** median lobe in ventral view **D** left paramere in external view. Scale bar: 0.5 mm.

**Figure 14.**
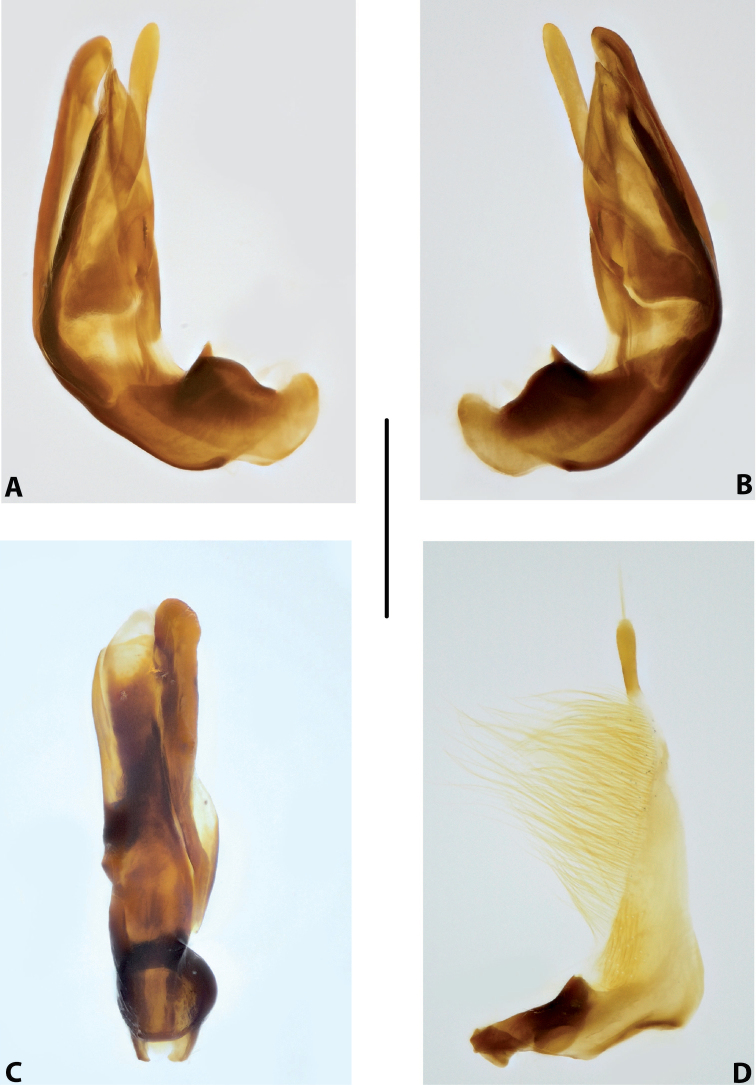
*Copelatus irregularis***A** median lobe in lateral view, right side **B** median lobe in lateral view, left side **C** median lobe in ventral view **D** left paramere in external view. Scale bar: 0.5 mm.

**Figure 15.**
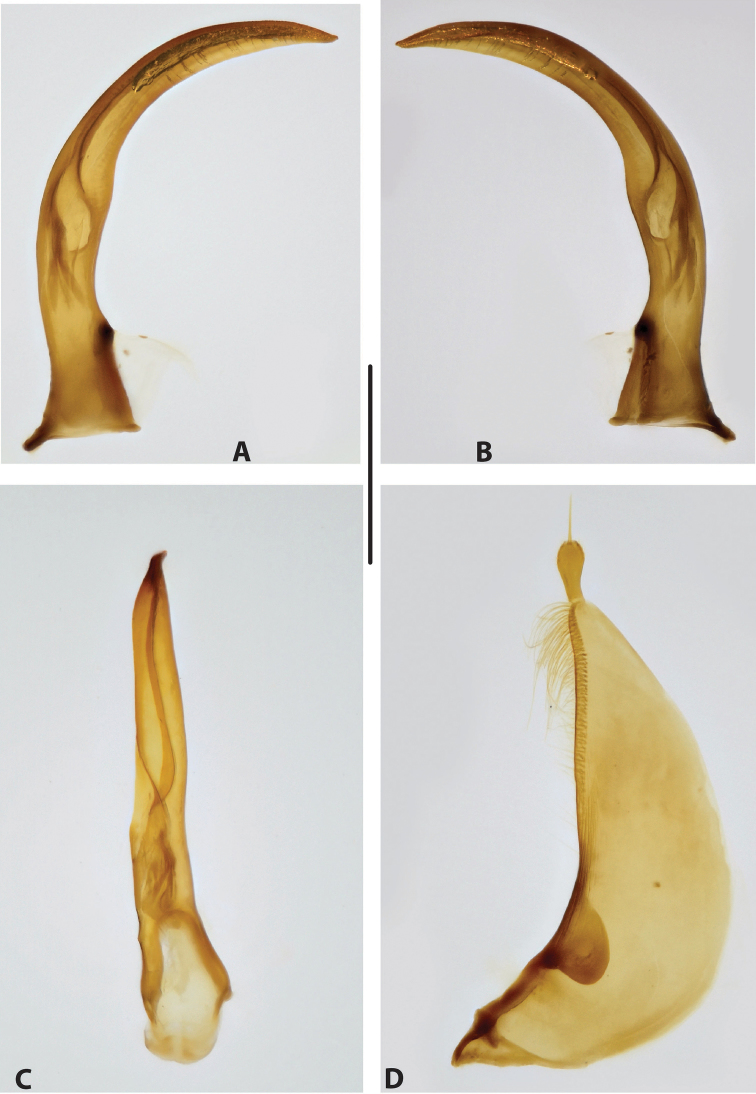
*Copelatus marginatus***A** median lobe in lateral view, right side **B** median lobe in lateral view, left side **C** median lobe in ventral view **D** left paramere in external view. Scale bar: 0.5 mm.

**Figure 16.**
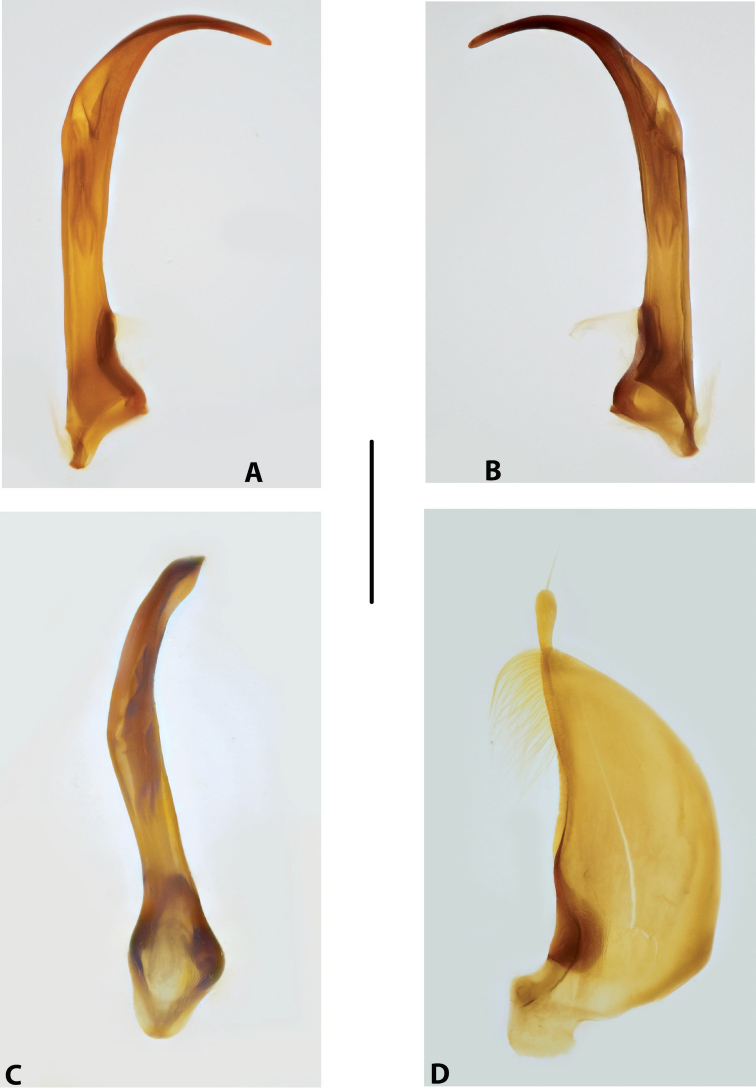
*Copelatus martinbaehri* sp. nov., paratype from Mount Tozer **A** median lobe in lateral view, right side **B** median lobe in lateral view, left side **C** median lobe in ventral view **D** left paramere in external view. Scale bar: 0.5 mm.

**Figure 17.**
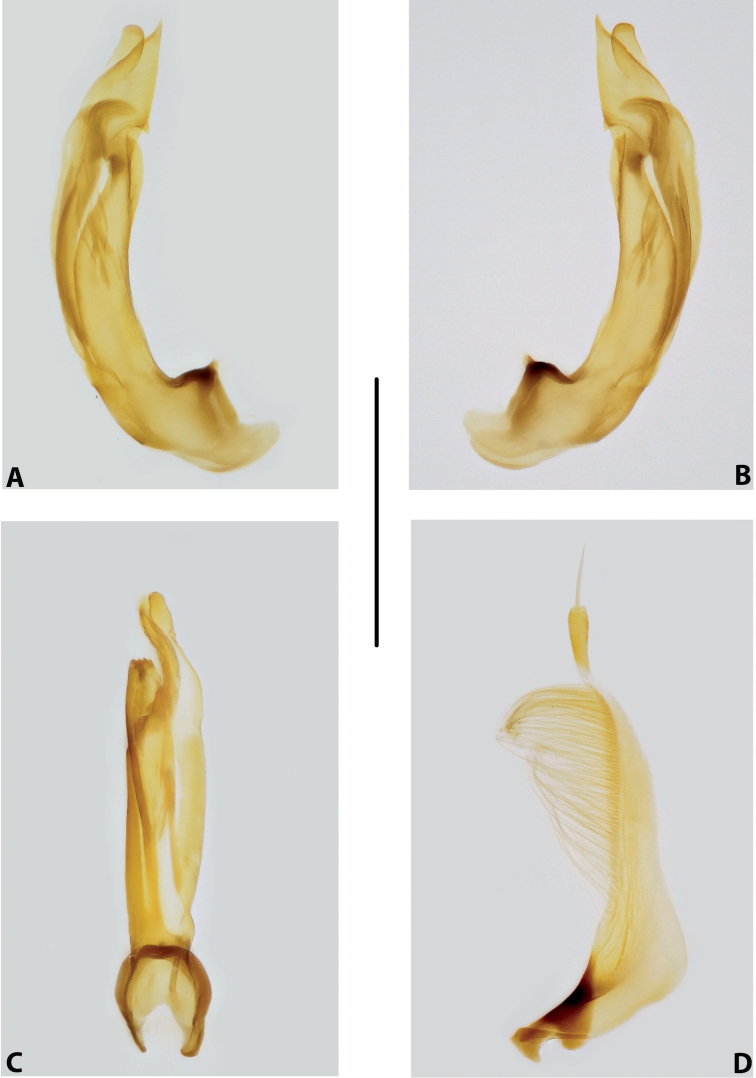
*Copelatus nigrolineatus***A** median lobe in lateral view, right side **B** median lobe in lateral view, left side **C** median lobe in ventral view **D** left paramere in external view. Scale bar: 0.5 mm.

**Figure 18.**
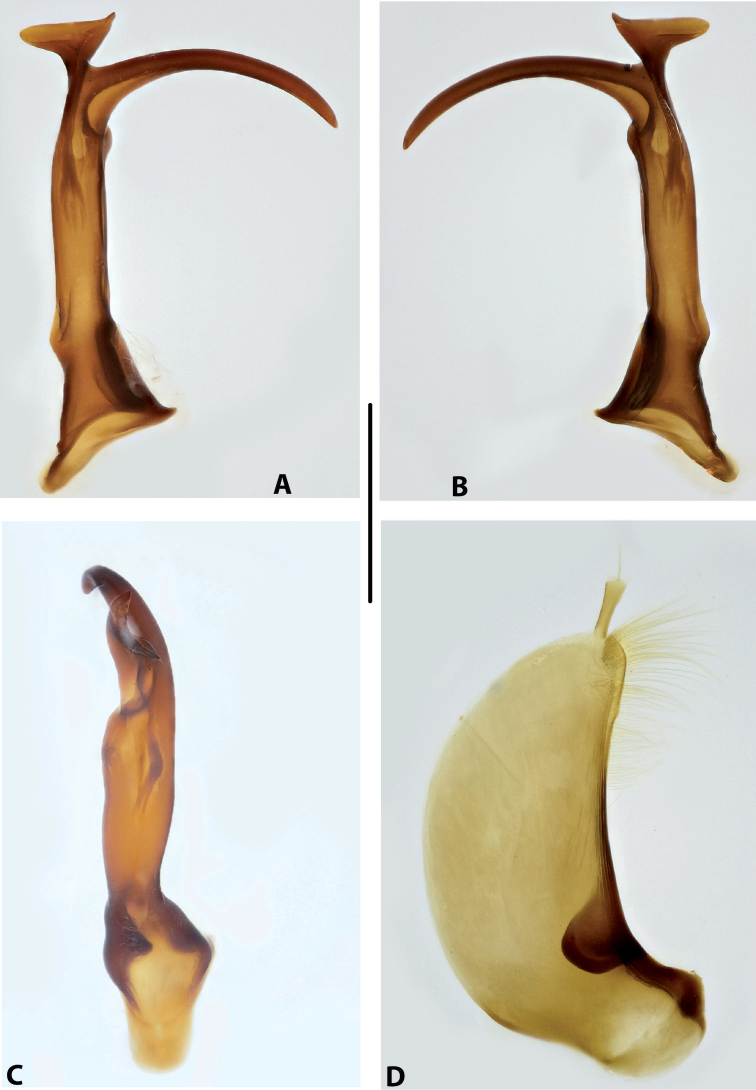
*Copelatus portior***A** median lobe in lateral view, right side **B** median lobe in lateral view, left side **C** median lobe in ventral view **D** left paramere in external view. Scale bar: 0.5 mm.

**Figure 19.**
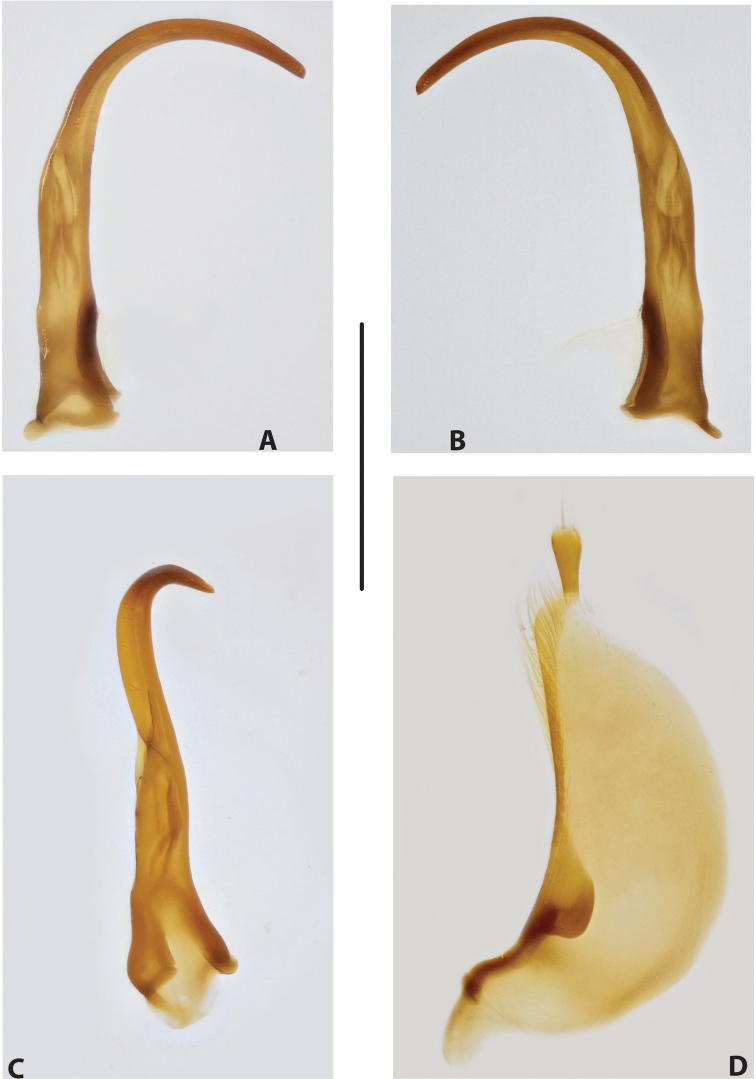
*Copelatus tenebrosus***A** median lobe in lateral view, right side **B** median lobe in lateral view, left side **C** median lobe in ventral view **D** left paramere in external view. Scale bar: 0.5 mm.

**Figures 20, 21.**
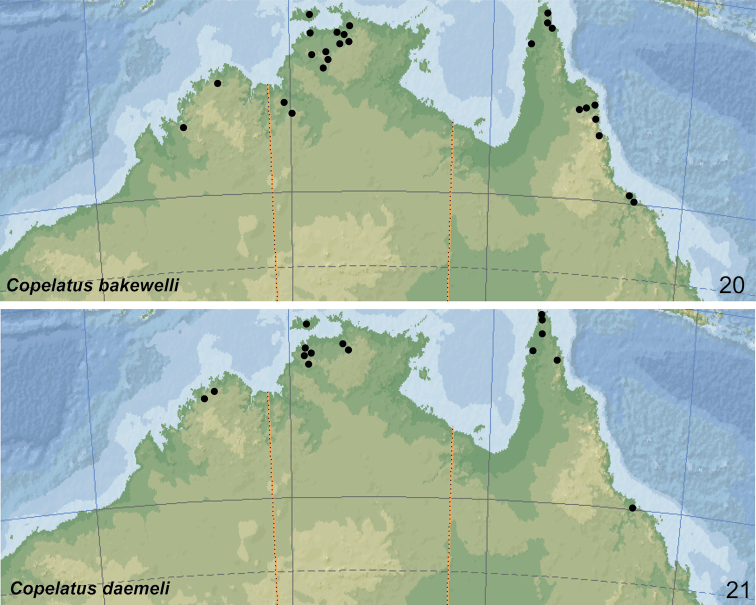
Distribution of **20***Copelatus bakewelli***21***C. daemeli*.

**Figure 22.**
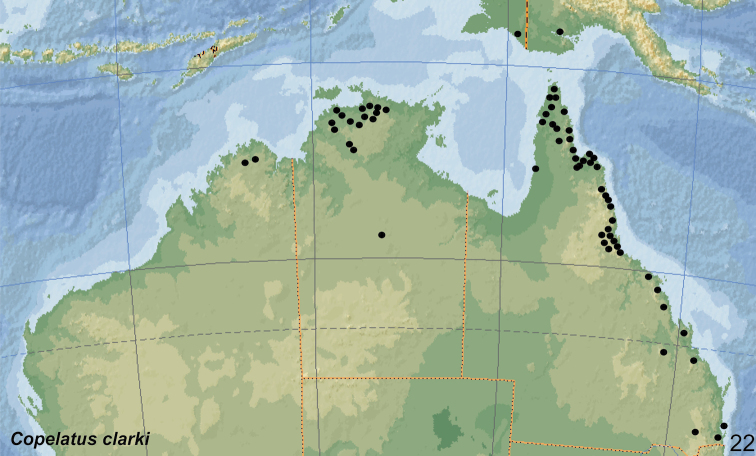
Distribution of *Copelatus clarki*.

**Figure 23.**
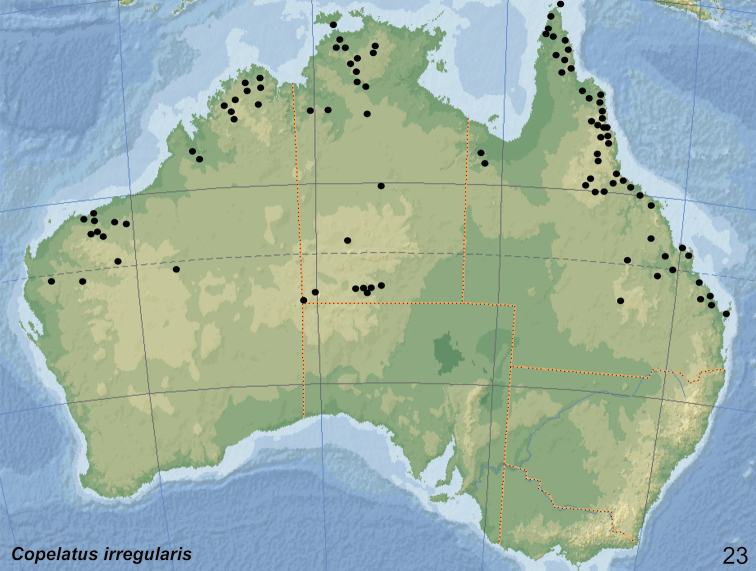
Distribution of *Copelatus irregularis*.

**Figure 24.**
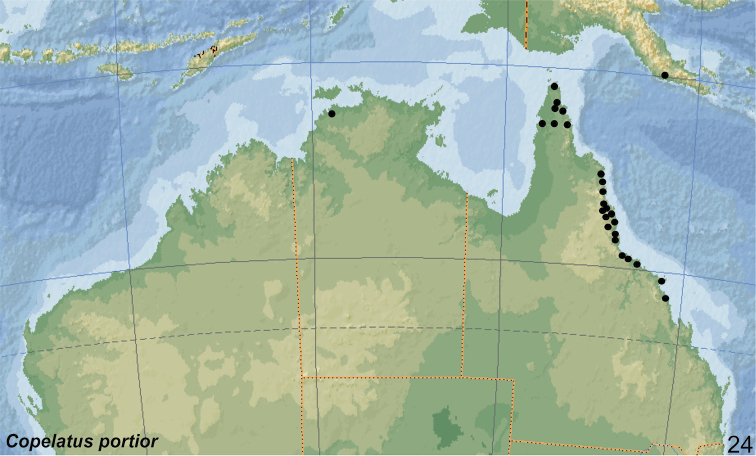
Distribution of *Copelatus portior*.

**Figure 25.**
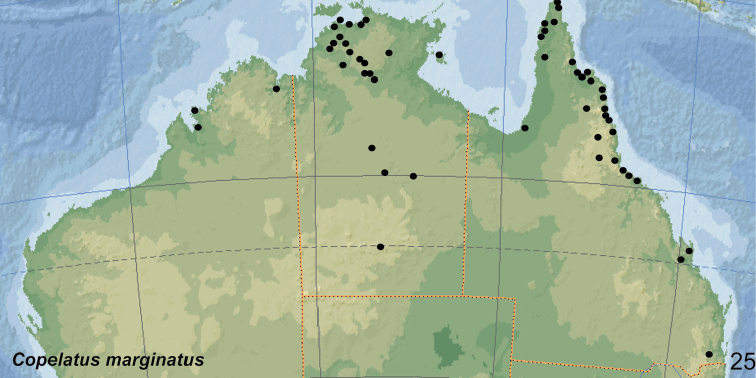
Distribution of *Copelatus marginatus*.

**Figure 26.**
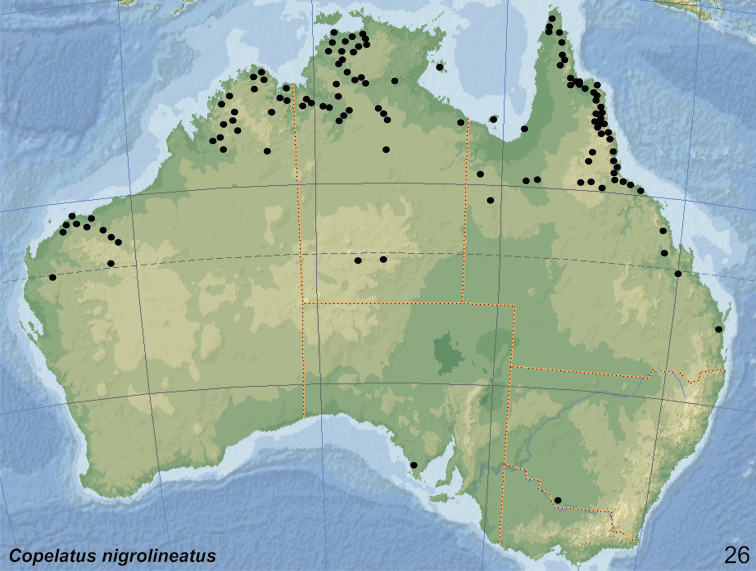
Distribution of *Copelatus nigrolineatus*.

**Figures 27, 28.**
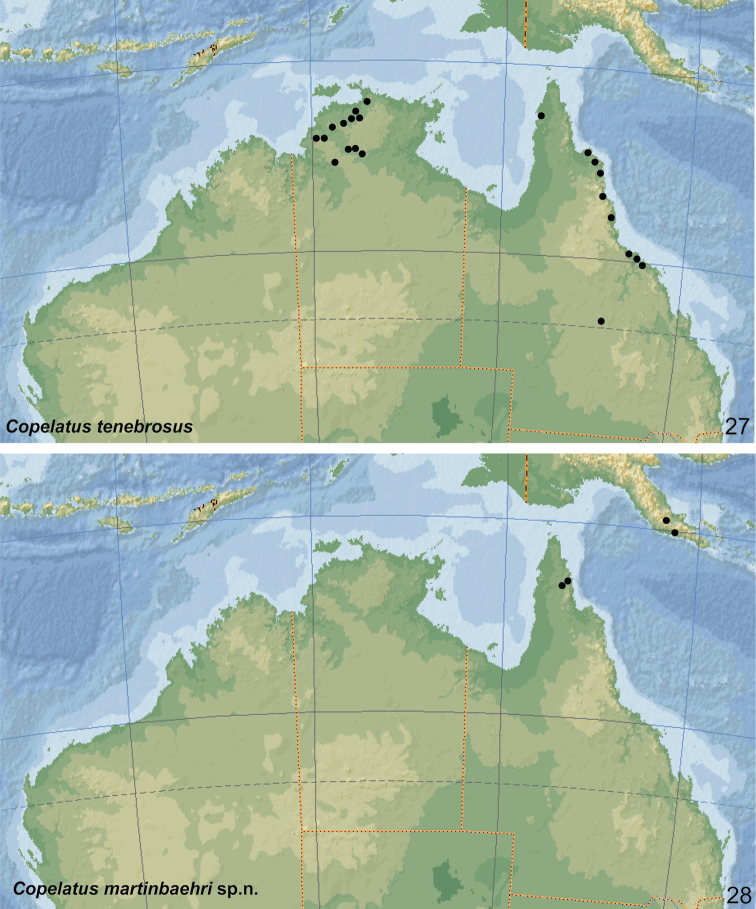
Distribution of **27***Copelatus tenebrosus***28***C. martinbaehri* sp. nov.

**Figure 29.**
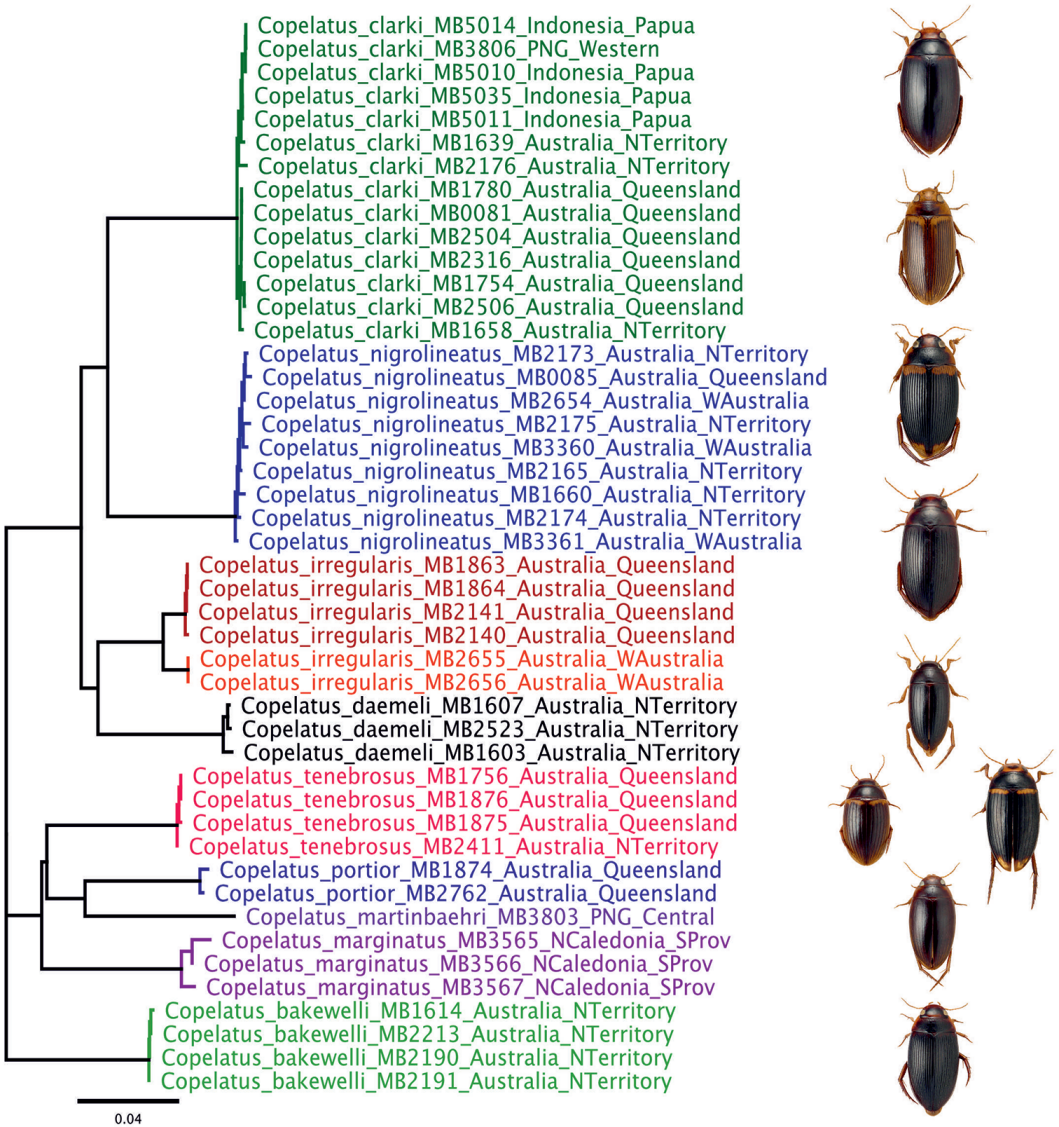
Maximum likelihood tree for Australian *Copelatus*. Neighbour joining tree (p-distances) calculated with Geneious (11.0.4.) software.

**Figure 30.**
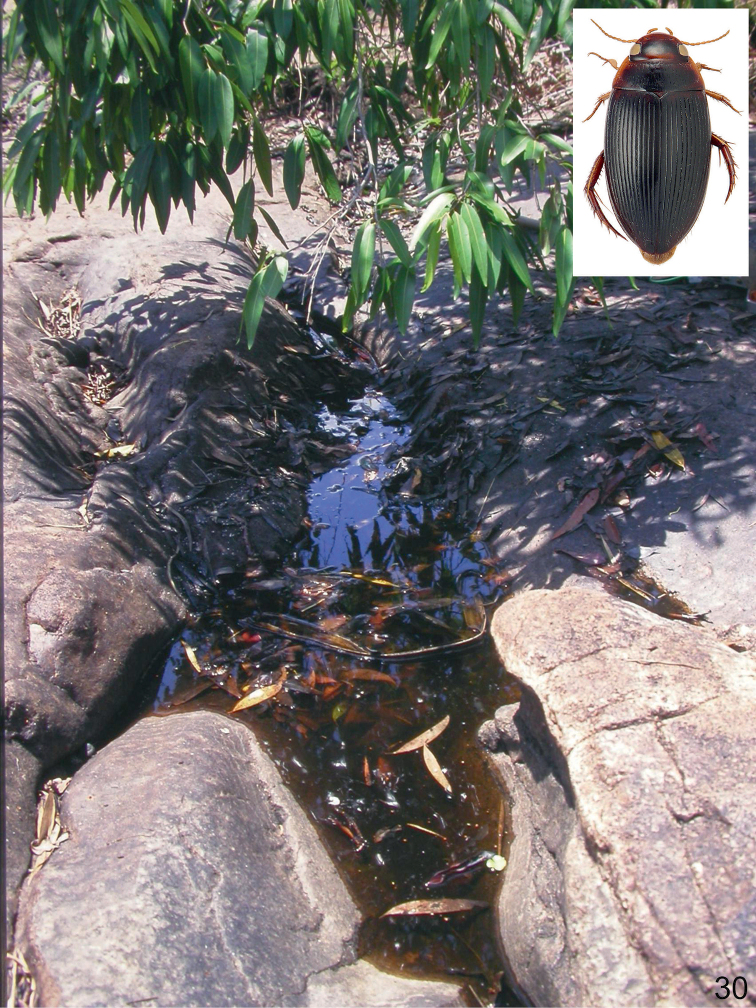
Habitat of *Copelatus bakewelli*, rocky pool, with rotten leaves near Gunlom Waterfall, Northern Territory. Photographs by L. Hendrich.

**Figure 31.**
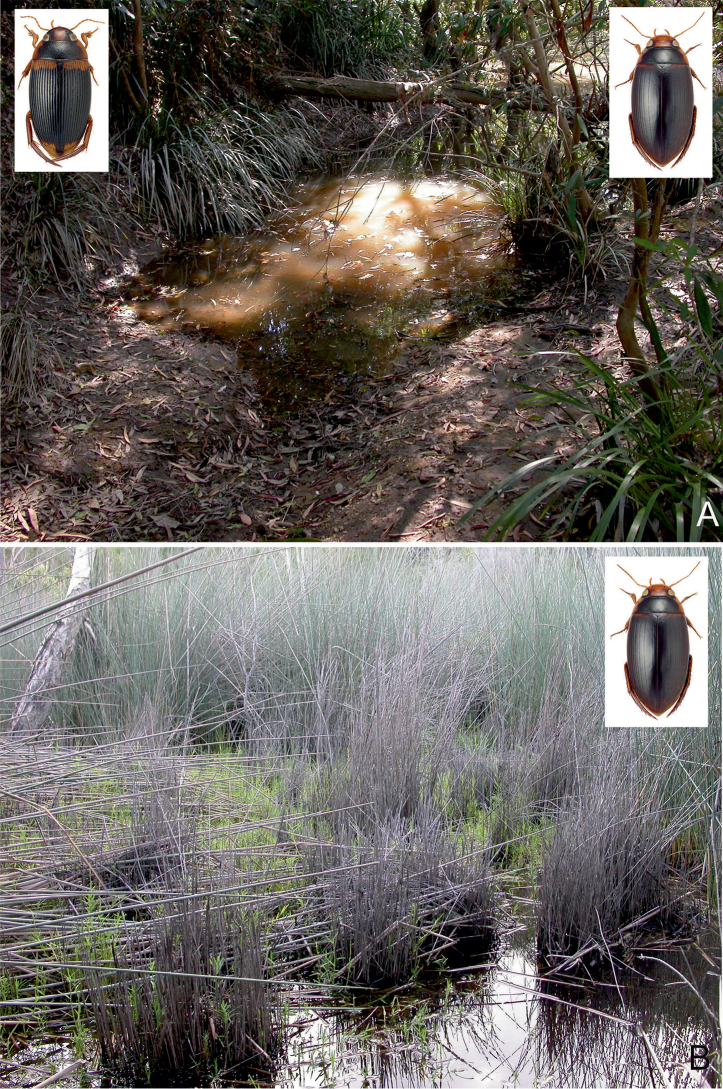
Habitat of *Copelatus irregularis* and *C. clarki***A** residual pool of the stream bed of Oakly Creek, Queensland (Loc. 28) **B** only of *C. clarki*, Stradbroke Island, temporary sedge swamp near Brown Lake, Queensland. Photographs by L. Hendrich.

**Figure 32.**
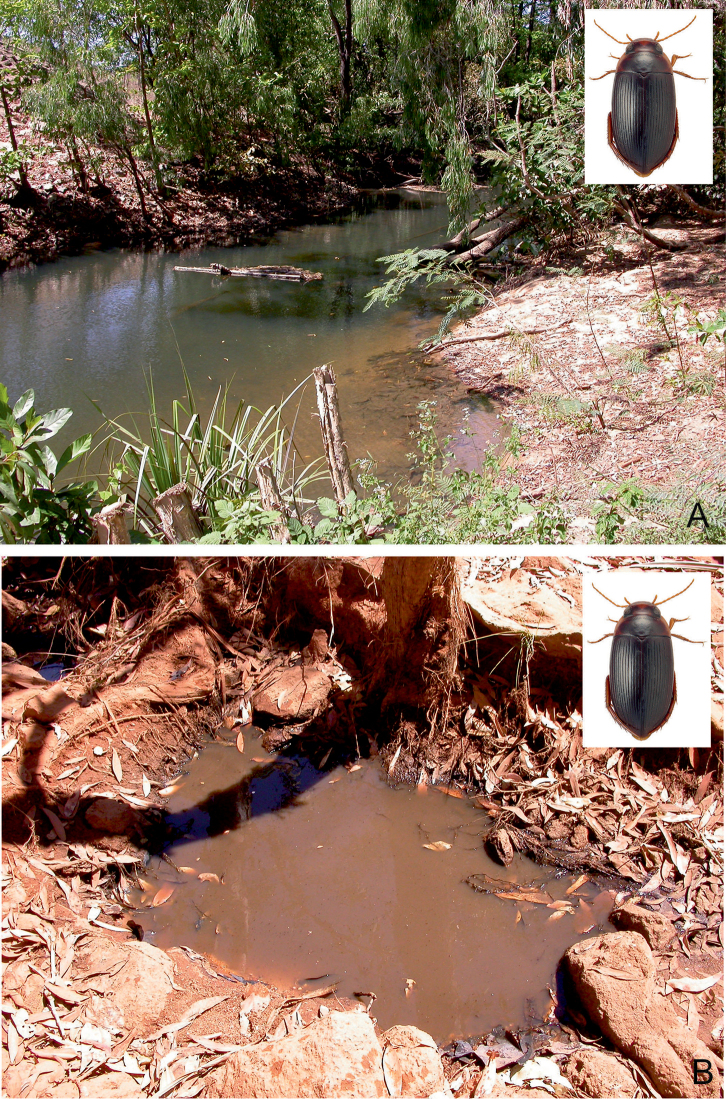
Habitat of *Copelatus daemeli***A** Finnis River, Northern Territory, all beetles were at the edge among rotten leaves and twigs **B** small puddle filled with leaf litter at the road to Gunlom, Kakadu National Park, Northern Territory. Photographs by L. Hendrich.

**Figure 33.**
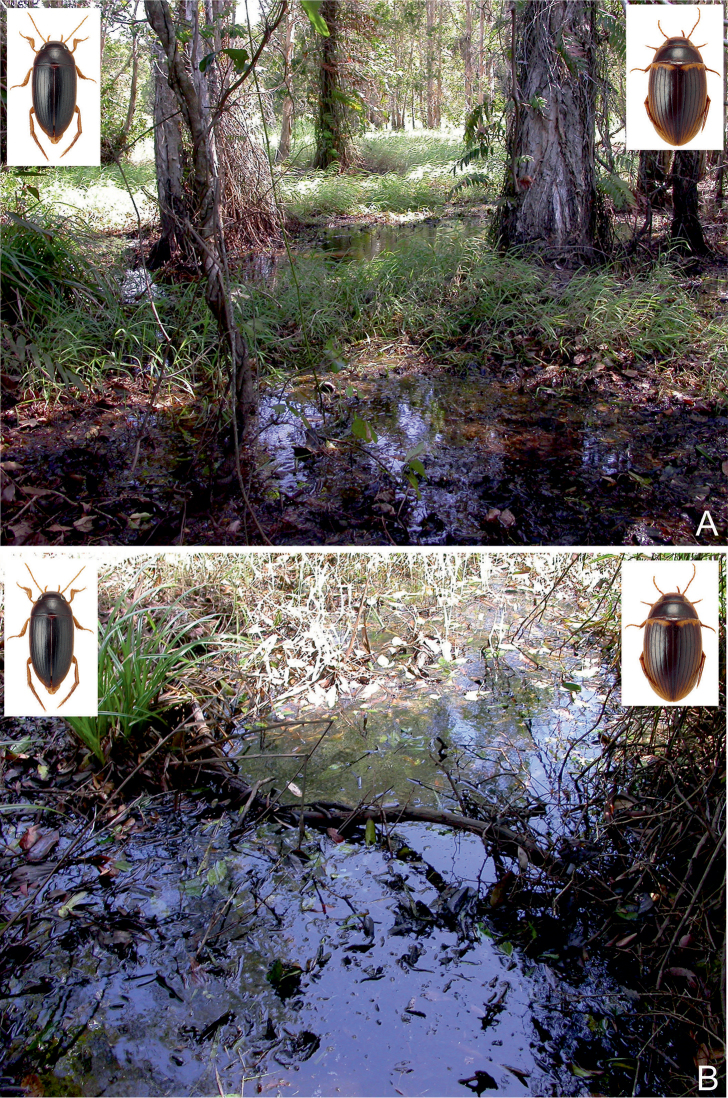
Habitat of *Copelatus tenebrosus* and *C. portior***A** Queensland, swamp forest at Cape Tribulation Road **B** shallow and ephemeral swampy puddles with rotten leaves, roots and twigs, Queensland, Cape Tribulation Road. Photographs by L. Hendrich.

**Figure 34.**
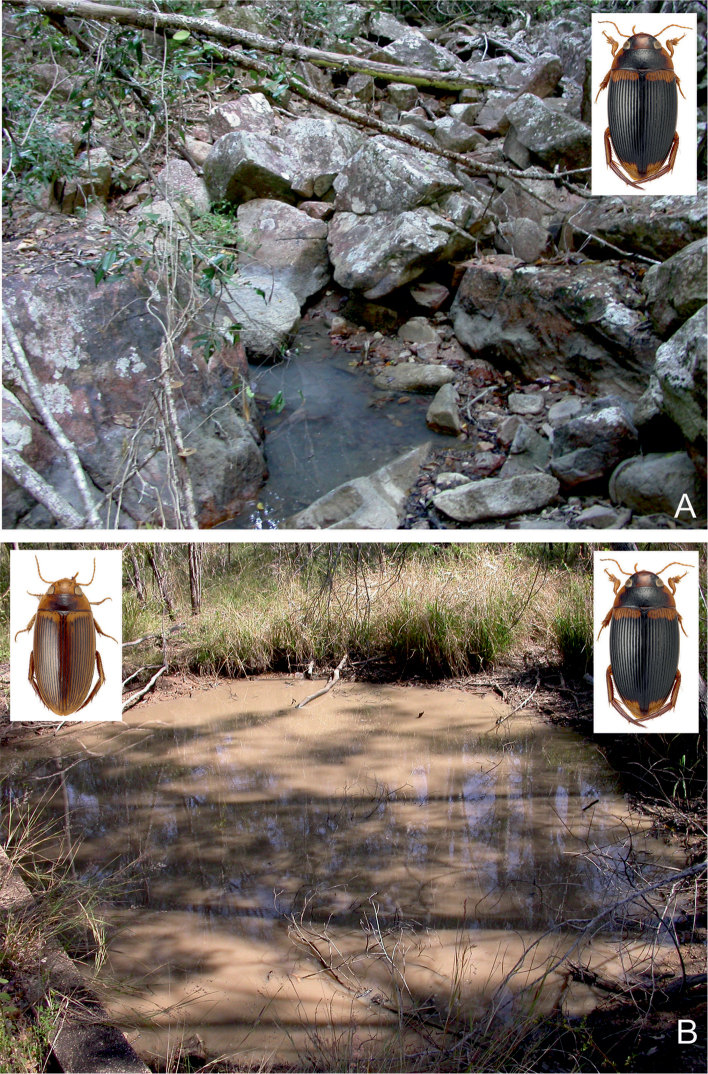
Habitat of *Copelatus irregularis***A** Queensland, Paluma Road 4 km west of Bruce Highway, Maiden Hair Fern Creek, 270 m, shady and rocky residual pools with rotten leaves **B** of *C. irregularis* and *C. nigrolineatus*, Queensland, Winfield, Winfield Road, muddy forest pool, rich in rotten leaves, near the street, 21 m. Photographs by L. Hendrich.

**Figure 35.**
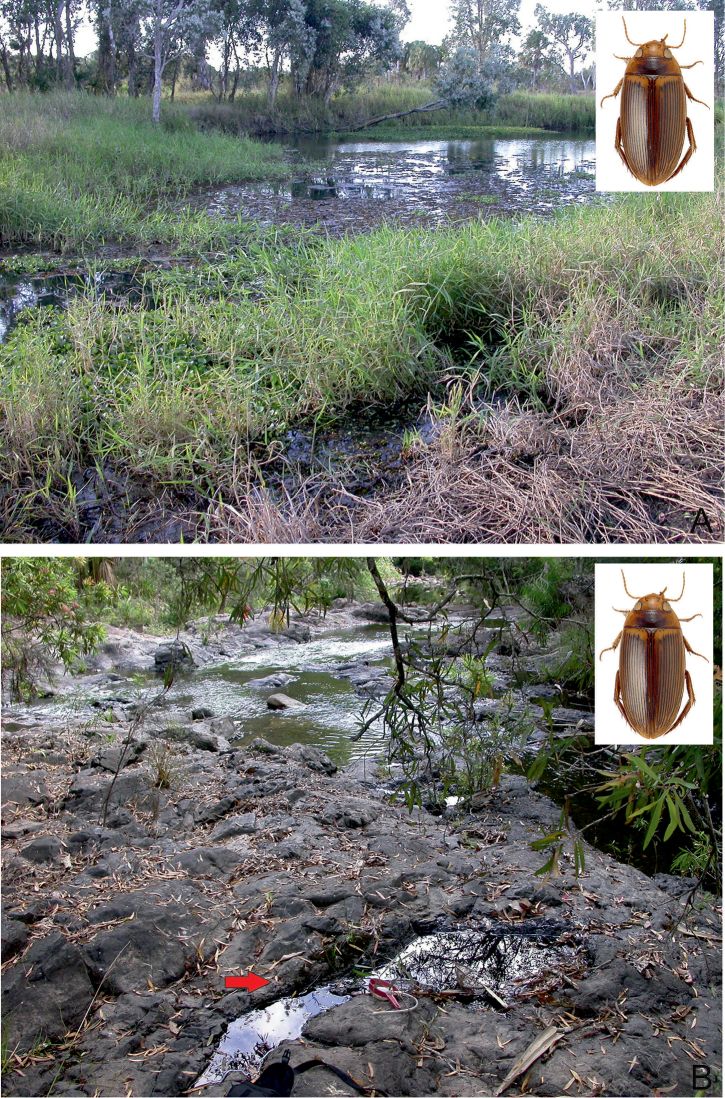
Habitat of *Copelatus nigrolineatus***A** Queensland, 19 km S Ayr, Bannister Lagoon at Bruce Highway, swamp, 20 m, beetles were among mats of floating grasses **B** Queensland, 18 km S Calen, Mt. Charlton-Calen Road, rock pools filled with leaf litter at Boulder Creek, 42 m. Photographs by L. Hendrich.

**Figure 36.**
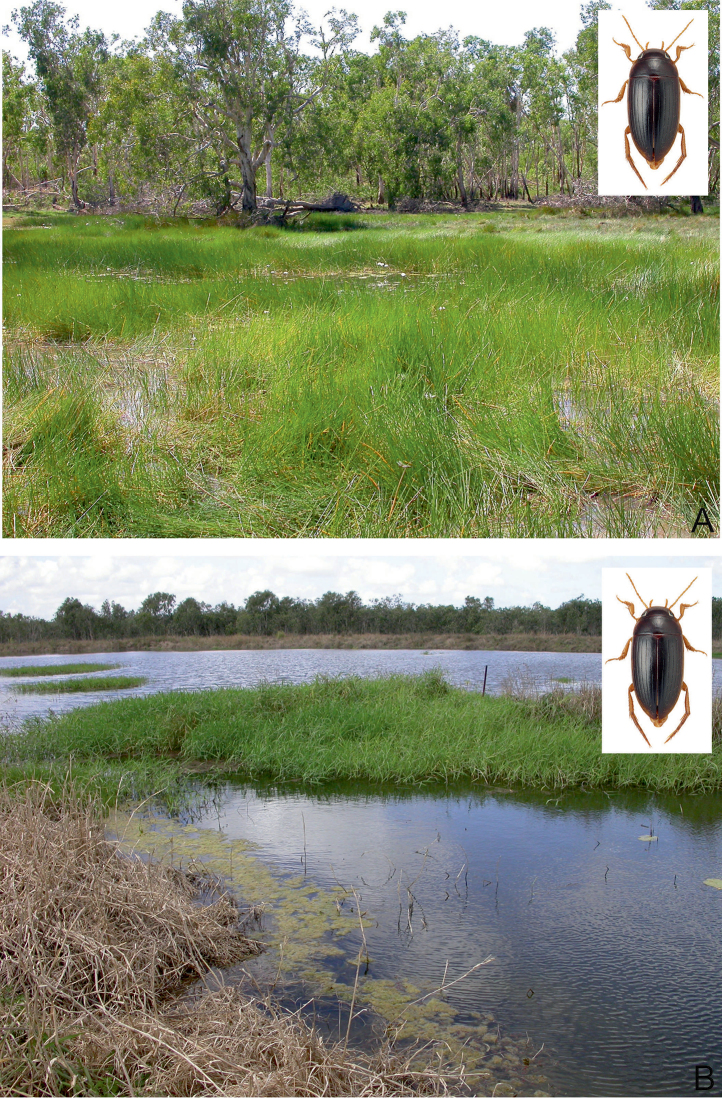
Habitat of *Copelatus tenebrosus***A** Northern Territory, Georgetown Billabong, 750 m east of Jabiru East, 30 m, beetles were in very shallow water among the sedges **B** Queensland, 10 km south of Mizani, Lake Kinchant, beetles were in shallow water among mats of floating grasses, 48 m. Photographs by L. Hendrich.

## Supplementary Material

XML Treatment for Copelatus bakewelli

XML Treatment for Copelatus clarki

XML Treatment for Copelatus daemeli

XML Treatment for Copelatus irregularis

XML Treatment for Copelatus marginatus

XML Treatment for Copelatus martinbaehri

XML Treatment for Copelatus nigrolineatus

XML Treatment for Copelatus portior

XML Treatment for Copelatus tenebrosus

## Footnotes

For full synonymy, see Sheth et al. 2018: 257 and Nilsson and Hájek 2019: 59.

dimorphic species
